# Neuroprotective Effects of Omentin-1 Against Cerebral Hypoxia/Reoxygenation Injury via Activating GAS6/Axl Signaling Pathway in Neuroblastoma Cells

**DOI:** 10.3389/fcell.2021.784035

**Published:** 2022-01-24

**Authors:** Xiaochen Niu, Ye Cheng, Meng Zhang, Luyang Du, Xue Wu, Chenxi Lu, Xiyang Li, Shuai Liu, Aizhen Zhao, Shaofei Zhang, Zhen Wu, Baoping Ding, Wenzhen Shi, Changyu Wang, Yang Yang, Ye Tian

**Affiliations:** ^1^ Xi’an Key Laboratory of Cardiovascular and Cerebrovascular Diseases, Xi’an No.3 Hospital, The Affiliated Hospital of Northwest University, School of Life Sciences and Medicine, Northwest University, Xi’an, China; ^2^ Key Laboratory of Resource Biology and Biotechnology in Western China, Ministry of Education, Faculty of Life Sciences, Northwest University, Xi’an, China

**Keywords:** ischemic stroke, omentin-1, GAS6/TAM, neuroprotection, oxidative stress

## Abstract

Ischemic stroke is characterized by insufficient blood supply to brain tissue and is associated with increased morbidity and mortality in adults worldwide. Growth arrest-specific protein 6 (GAS6) is a vitamin K-dependent protein and is widely expressed in the central nervous system. The biological functions of GAS6 are mediated by the interaction with TAM (Tyro3, Axl and Mertk) receptors, including cell survival and proliferation, immune regulation and apoptosis. Omentin-1, also known as intelectin-1 (ITLN-1), is a novel adipocytokine that is involved in a variety of biological events, such as insulin resistance, endothelial dysfunction, programmed cell death and metabolic disorders. Our previous study has found that omentin-1 act as a novel regulator of vascular and anti-apoptotic response in cerebral ischemia. However, the specific molecular mechanism of omentin-1’s protective effect on cerebral ischemia-reperfusion injury (IRI) is still unclear. First, the toxicity of recombinant human omentin-1 (rh-omentin) was assessed and a safe concentration was chosen for the next experiments. Then, rh-omentin exerted neuroprotection against hypoxia/reoxygenation (H/R) injury in N2a cells, indicated by increased cell viability, decreased LDH, ROS generation, and cell apoptotic rate. Furthermore, the similar protective effect was observed in omentin-1 overexpression cells constructed by lentivirus transfection. Rh-omentin could also inhibit H/R-induced apoptotic molecules, oxidative stress molecules, and GAS6/Axl signaling molecules which as evidence by increased omentin-1, GAS6, Axl, p-Axl, NQO1, HO-1, Nrf2, Bcl2 and decreased Bax expressions. However, GAS6 siRNA could reverse rh-omentin-induced neuroprotection and the levels of these molecules mentioned above. In conclusion, these findings suggest that omentin-1 treatment exerts neuroprotection against H/R injury partly via activating GAS6/Axl signaling at least. Therefore, these finding may favor omentin-1 a potential neuroprotective drug candidate to alleviate ischemia-reperfusion injury in clinic.

## Introduction

Ischemic stroke is characterized by insufficient blood supply to brain tissue and is associated with increased morbidity and mortality in adults worldwide (Mendelson and Prabhakaran, 2021). Timely vascular reconstruction is the standard treatment for cerebral ischemia, which is essential to alleviate ischemia injury and restore brain function. However, reperfusion treatment also induces additional damage to ischemic tissue, defined as cerebral ischemia-reperfusion injury (IRI) in clinic (Spescha et al., 2015). From the perspective of pathogenesis, the occurrence of cerebral IRI can trigger a variety of apoptosis pathways, leading to excessive inflammation response, uncontrollable oxidative stress, DNA fragmentation and mitochondrial dysfunction (Nishimura et al., 2008; Kruyt et al., 2010). Hence, understanding the molecular feature of cerebral IRI and alleviating IR mediated neuronal damage can increase the therapeutic efficiency of IR treatment.

Omentin-1, also known as intelectin-1 (ITLN1), is a novel adipocytokine that is mainly expressed in stromal vascular cells of omental adipose tissue and can also be expressed in airway goblet cells, mesothelial cells, vascular cells, and so on (Rong-Ze et al., 2006). Studies found that omentin-1 is involved in a variety of biological events, such as insulin resistance, endothelial dysfunction, programmed cell death and metabolic disorders (Yin et al., 2017; Leandro et al., 2021). Maruyama et al. confirmed that omentin-1 can promote endothelial cell function and angiogenesis in response to ischemia and inhibit apoptosis in rats with unilateral hind limb surgery (Maruyama et al., 2012). As omentin-1 has been increasingly identified, it has become promising targets for anti-oxidative stress, anti-apoptosis and anti-inflammatory therapies (Kaho et al., 2016; Yin et al., 2017; Naibing et al., 2019). Our previous study has found that omentin-1 act as a novel regulator of vascular and anti-apoptotic response in cerebral ischemia (Gu et al., 2017). However, the specific molecular mechanism of omentin-1’s protective effect on cerebral IRI is still unclear.

Growth arrest-specific protein 6 (GAS6) is a vitamin K-dependent protein, and is widely expressed in the central nervous system (van der Meer et al., 2014; Shafit-Zagardo et al., 2018). Axl is a member of the TAM (Tyro3, Axl and Mertk) family of receptor tyrosine kinases which are all activated by the ligand GAS6 (Graham et al., 2014). Multiple studies have confirmed that GAS6/Axl pathway interacts with various pleiotropic pathways, including the phosphoinositide 3-kinase/Akt (PI3K/Akt) pathway, the mitogen-activated protein kinase/extracellular signal-regulated kinase (MAPK/ERK) pathway, and the signal transducer and activator of transcription 3 (STAT3) pathway, and plays important roles in cell survival, proliferation, migration and mitogenesis (Wu et al., 2017). Notably, the role of GAS6/Axl pathway in organ protection under various pathological conditions, especially brain protection, has been extensively studied (Tong et al., 2017; Wu et al., 2018). However, the protective effect of omentin-1 against cerebral IRI has not been fully evaluated yet and the underlying molecular mechanism of its beneficial effect remains unknown.

Therefore, this study aimed to investigate the protection of omentin-1 in cerebral IRI by establishing a hypoxia/reoxygenation (H/R) model in N2a cells. Moreover, the GAS6/Axl signaling pathway and its downstream targets were investigated to further elucidate the underlying mechanisms of omentin-1’s neuroprotective effect. The findings of the present study may shed new light on the treatment of cerebral IRI.

## Materials and Methods

### Cell Culture and Treatment and the Construction of an Appropriate H/R Injury Model

N2a cells (purchased from ATCC, Rockville, MD, United States) were maintained in Dulbecco’s modified Eagle medium (DMEM) containing 10% fetal bovine serum (FBS) and incubated at 37°C under 5% CO_2_. The culture medium was renewed every 3 days. Differentiated N2a cells obtained by serum deprivation were used in all functional experiments. Neuron markers (Tubulin beta 3, TUBB3; Growth-associated protein 43, GAP43) were used to determine the degree of cell differentiation. Rh-omentin (ProSpec-Tany TechnoGene Ltd., Rehovot, Israel) was dissolved in ddH2O water and diluted with immediately prior to the experiment. The controls were treated with equal volume of ddH_2_O. An anoxic tank (Longyue Company, Shanghai, China) was used to form an anaerobic environment, and the oxygen concentration was less 6–8% (v/v). After 12 h of hypoxia and 6 h of reoxygenation, the degree of injury for the cells was approximately 50%, and this was used as the optimal injury model for subsequent experiments (Supplementary Figures S1A,B). To explore the neuroprotective effect of rh-omentin against H/R-induced neuronal death, N2a cells were seeded in 60-mm culture dishes. First, the cells were treated with different concentrations of rh-omentin (250, 500, 750, 1,000 or 1,250 ng/ml) for 24 h to detect the toxic effect of rh-omentin. Then, the cells were treated with different concentrations of rh-omentin (250, 500, 750, 1,000 or 1,250 ng/ml) for 3 h before exposure to H/R unless otherwise indicated. After the treatments, the cells were harvested for further analysis.

### Cell Viability Assay

The treated cells were mixed with Muse™ Count and Viability Regent for 5 min and then detected by Muse™ Cell Analyzer (Merck KGaA, Darmstadt, Germany). The operations were carried out in strict accordance with the instruction manual of the instrument and kit.

### ROS Production Assay

Dihydroethidium (DHE) staining was applied to measure the generation of ROS. N2a cells were incubated with 10 μM DHE for 20 min at 37°C in the dark. The fluorescent intensity was immediately observed by fluorescence microscopy (Invitrogen EVOS M5000, Thermo Fisher Scientific, Waltham, MA, United States), and the fluorescent signal was quantified using ImageJ Software (National Institutes of Health, Bethesda, MD, United States).

### LDH Release and Apoptosis Assay

500 μl supernatant of N2a cells were taken, and the level of LDH was detected by LDH detection kit (Nanjing Jiancheng Bioengineering Institute, Nanjing, Jiangsu, China). The Muse™ Cell Analyzer was used according to the manufacturer’s recommendations. Briefly, the treated cells were harvested and stained with Muse™ Annexin V & Dead Cell Kit in the dark, and living cells, early apoptotic cells, late apoptotic cells, and necrotic cells were detected. Untreated populations were used to define the levels of apoptosis and the number of dead cells under basic conditions. Each experiment was performed in triplicate.

### Western Blot Analysis

Protein expression was determined by western blot analysis as previously described (Zhu et al., 2017). Cells were harvested after various treatments and then lysed in RIPA buffer for 30 min at 4°C. The lysates were centrifuged at 12,000 rpm for 5 min at 4°C. Total protein concentration was detected by using a bicinchoninic acid (BCA) protein assay kit (Beyotime Institute of Biotechnology, Jiangsu, China), and 20 μg of total protein was resolved by sodium dodecyl sulfate–polyacrylamide gel electrophoresis (SDS-PAGE). The separated proteins were transferred to polyvinylidene difluoride (PVDF) membranes. The membranes were blocked with 5% defatted milk and incubated with antibodies against omentin-1 (1:1,000, ProteinTech Group, Inc., Wuhan, Hubei, China), β-actin (1:1,000, Servicebio Biotechnology Co., Ltd., Wuhan, Hubei, China), GAS6, Axl, p-Axl (1:1,000, Bioss Biotechnology Co., Ltd., Beijing, China), NQO1 (1:500, Santa Cruz Biotechnology, Dallas, TX, United States), HO-1 (1:1,000, Abcam biotechnology, Cambridge, United Kingdom), Bax (1:1,000, Cell Signaling Technology, Inc., Danvers, Massachusetts, United States), Nrf2, Bcl2 (1:1,000, Boster Biological Technology Co., Ltd., Wuhan, Hubei, China) and secondary antibodies (goat anti-mouse IgG, 1:5,000, Cell Signaling Technology, Inc., Danvers, Massachusetts, United States; goat anti-rabbit IgG, 1:5,000, Boster Biological Technology Co., Ltd., CA, United States). The fluorescent signal was detected using a MiNiChemi610 imaging system (SAGECREATION Co., Ltd., Beijing, China), and the signal was quantified using ImageJ software (National Institutes of Health, Bethesda, MD, United States).

### Real-Time Fluorescence Quantitative Polymerase Chain Reaction (qPCR) Assay

The experimental cells were lysed by Trizol Reagent kit and total RNA was extracted. The Evo M-MLV RT kit (Accurate Biotechnology, Changsha, Hunan, China) was used for reverse transcription of RNA into cDNA. The qRT-PCR was performed by a kit containing 2 × SYBR^®^ Green Pro Taq HS Premix II (Accurate Biotechnology, Changsha, Hunan, China). The amplification conditions were as follows: an initial denaturation at 95°C for 3 min, followed by 40 cycles at 95°C for 15 s, 57°C for 15 s, and 72°C for 20 s. Gene expression was quantified relative to β-actin expression using the 2^−ΔΔCt^ method. The sequence of β-actin forward primer: 5′-ATA​AAT​GGA​GCA​CCT​CAA​CCT-3′, reverse primer: 5′-TCT​CCT​TCT​GCG​AGC​GTC​CT-3'. The sequence of Omentin-1 forward primer: 5′-TGG​CCA​GAG​GGA​ATT​TAC​TGC-3′, reverse primer: 5' -CAC​CGA​TGC​AGT​GAT​GTT​CAG- 3'. The sequence of GAS6 forward primer: 5′-CGA​TTA​ACC​CTC​GCC​TGG​AT-3′, reverse primer: 5′-GCG​ATG​TTC​GGG​TGT​AGT​TG-3'. All experiments were performed in triplicate.

### Immunofluorescence Staining

Cell immunofluorescence staining was performed as describe before. Briefly, the cells were cleaned with PBS for three times and fixed with 4% paraformaldehyde at room temperature for 15 min. 0.2%Triton X-100 was added for permeation at room temperature for 10 min, followed by 5% bovine serum albumin for 1 h. Then a primary antibody diluted with 1% bovine serum albumin was added overnight at 4°C. After washing with PBS for three times, the secondary antibody diluted with 1% bovine serum albumin was added and placed at room temperature for 1 h. Finally, DAPI was added and incubated for 10 min. PBS was cleaned for three times and then sealed and photographed. Primary antibodies of Omentin-1, TUBB3, and GAP43 (1:200, ProteinTech Group, Inc., Wuhan, Hubei, China) and their corresponding secondary antibodies used according to the instructions.

### GAS6 Knockdown by Small Interfering RNA (siRNA)

The optimal sequence and concentration of siRNA were selected by cell viability, mRNA and protein expressions (data not shown). The sequences of siRNA targeting murine GAS6 (sense, 5′-GGA​CAC​ACU​UAA​GAC​ACA​UTT-3′; antisense, 5′-AUG​UGU​CUU​AAG​UGU​GUC​CTT-3′) were synthesized by Sangon Biotechnology (Shanghai, China). The siRNA was used to knockdown GAS6 in N2a cells. For transfection, cells were seeded in 60 mm culture dishes to grow to about 60–70% confluence. According to the manufacturer’s recommendations, cells were transfected with siRNA molecules targeting GAS6 by using Lipofectamine 2000 (Invitrogen, Thermo Fisher Scientific, Carlsbad, CA, United States) for at least 4 h in medium without antibiotics, then cells were incubated for 24 h.

### Lentiviral Production and Infection of N2a Cells

The sequence used for PCR amplification of omentin-1 (omentin-1-CDS-F: tga​gtc​gcc​cgg​ggg​aat​tcC​TCG​AGG​CCA​CCA​TGA​CCC​AAC​TGG​GAT​TCC​T; omentin-1-CDS-R: cgt​cat​cgt​ctt​tgt​aat​cTC​TAG​AGC​GAT​AAA​ACA​GAA​GCA​CAG​C) were designed according to its mRNA sequence obtained from the Addgene (http://www.addgene.org/). The double chains were formed and inserted into plenti-UCOE-SFFV vectors (GENECARER Co., Ltd., Xi’an, Shaanxi, China) to construct a plenti-UCOE-SFFV vector expressing targeting omentin-1 and a scrambled negative control plenti-UCOE-SFFV vector. The plenti-UCOE-SFFV-omentin-1 was used for transfected 293T cells using Lipofectamine 2000 (Invitrogen, Life Technologies, Carlsbad, CA, United States) for at least 1 h in medium without antibiotics. Omentin-1 overexpression (omentin-1-OE) cells were generated by lentivirus collected and applied to N2a cells.

### Statistical Analyses

Data were analyzed by using GraphPad Prism 9.0.0 (GraphPad Software Inc., San Diego, CA, United States). All values are presented as the mean ± standard deviation (SD). For the difference analysis of experimental data, t test was used for any two groups, and one-way ANOVA was used for more than two groups. *p* < 0.05 indicated that the data had significant differences.

## Results

### Expression of Omentin-1 in N2a Cells Injured by H/R

**FIGURE 1:**
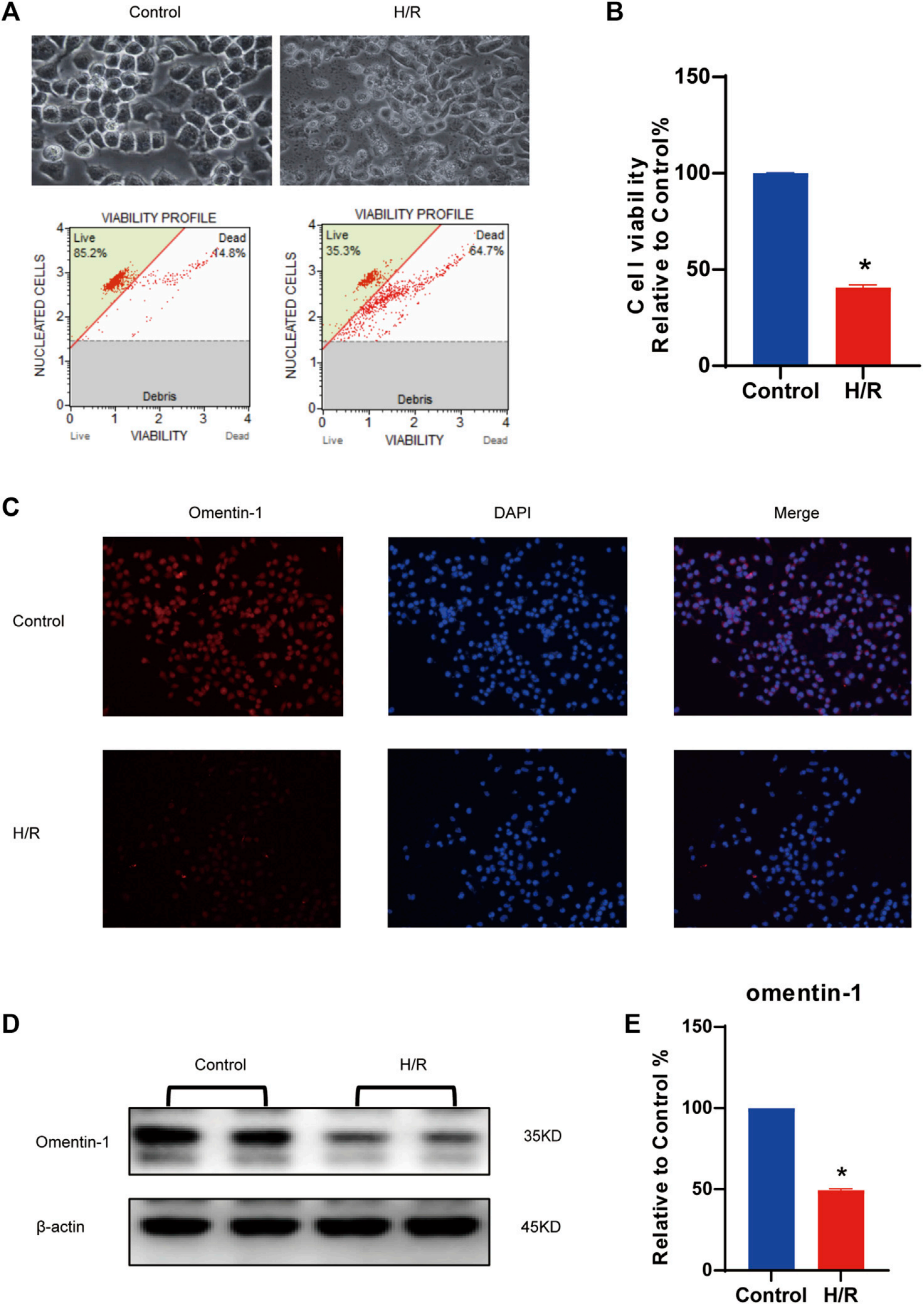
Expression of omentin-1 in N2a cells injured by H/R. **(A)** H/R model was constructed in N2a cells. **(B)** Statistical graph of cell viability. **(C)** Omentin-1 expression in N2a cells injured by H/R detected by immunofluorescence. **(D)** The representative images of omentin-1 detected by Western blot. **(E)** Quantitative analysis of Western blot by normalizing to β-actin. Error bars show the standard deviation for *n* = 6 measurements from representative experiments. *p < 0.05 vs. the Control group.

*In vitro* experiments, a H/R model was established. The results shown that H/R injury could reduce cell viability to about 50%, so this was used as the optimal injury model for subsequent experiments. (vs. the control group, Figures 1A,B, *p* < 0.05). Compared with the control group, H/R injury could significantly decrease omentin-1 expression (Figure 1C, *p* < 0.05). As shown in Figures 1D,E, western blot results shown that H/R suppressed the expression of omentin-1 in N2a cells (vs. the control group, *p* < 0.05). As shown in Supplementary Figures S2A,B, western blot results shown that H/R suppressed the expression of GAS6 in N2a cells (vs. the control group, *p* < 0.05). At mRNA level, omentin-1 and GAS6 were down-regulated in H/R treated N2a cells (vs. the control group, Supplementary Figures. 2C,D, *p* < 0.05). To accurately mimic neurons, neuronal markers in N2a cells were examined. Compared with DMEM containing 10% FBS (DMEM-FBS), serum-free medium (DMEM) increased the expression of GAP43 and TUBB3 (Supplementary Figures 3A,B, *p* < 0.05). However, H/R treatment reduced the expression of GAP43 and TUBB3 (vs. the DMEM group, *p* < 0.05).

### Toxic Effect of Rh-Omentin on N2a Cells

**FIGURE 2:**
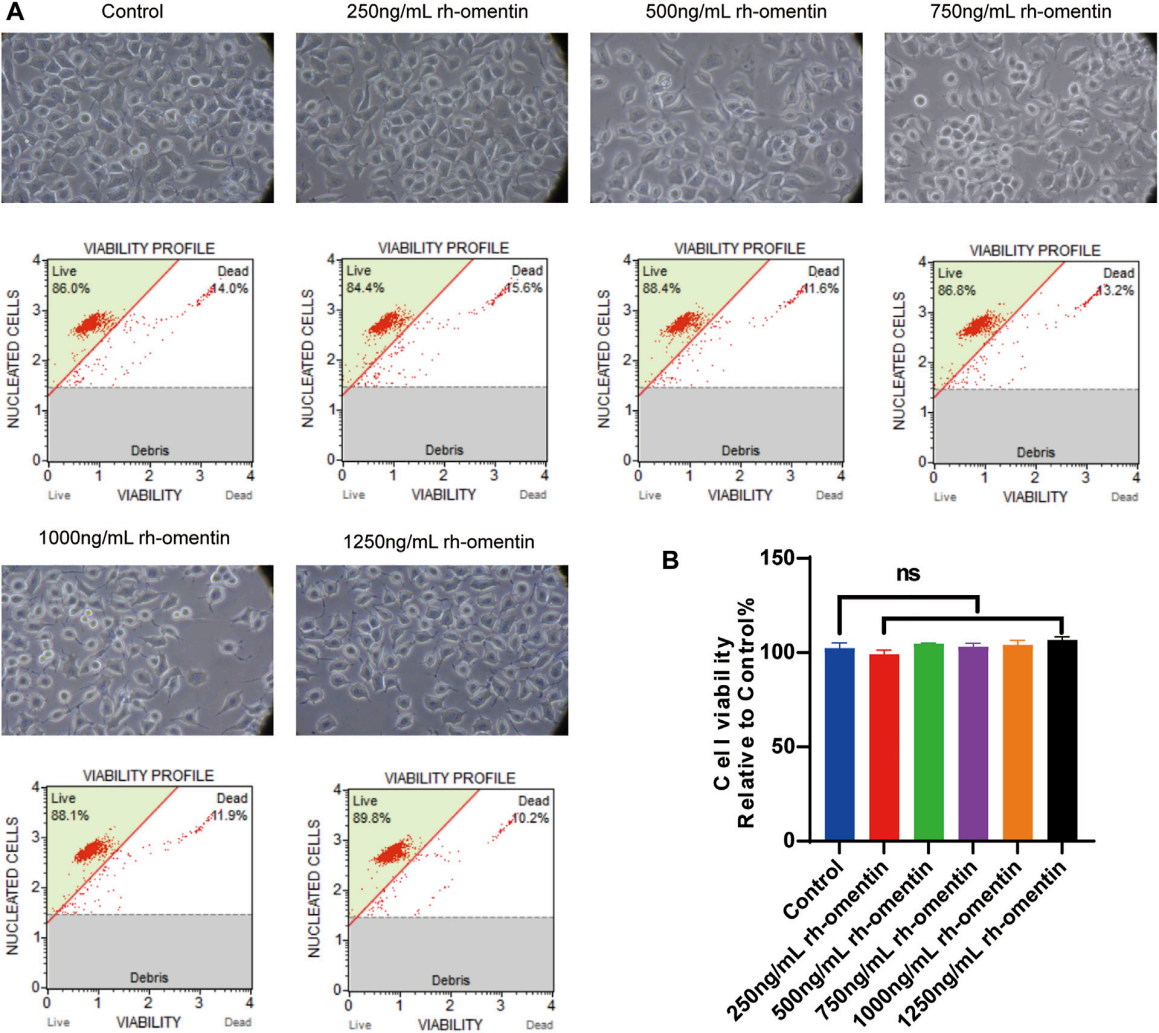
Toxic effect of rh-omentin on N2a cells. **(A)** N2a cells were treated with rh-omentin at the different concentrations (250, 500, 750, 1,000 or 1,250 ng/ml) for 24 h. Cell morphology was observed under an inverted/phase contrast microscope, and images were obtained. **(B)** Statistical graph of cell viability. Error bars show the standard deviation for *n* = 6 measurements from representative experiments. ns, non-signifcant.

Subsequently, the toxic effect of rh-omentin was evaluated. N2a cells were exposed to various concentrations of rh-omentin (250, 500, 750, 1,000, or 1,250 ng/ml) for 24 h. Notably, even the maximum concentration of rh-omentin (1,250 ng/ml) did not decrease cell viability and change cell morphology (Figures 2A,B, vs. the control group, *p* > 0.05).

### Rh-Omentin Protected N2a Cells Against H/R-Induced Injury

**FIGURE 3:**
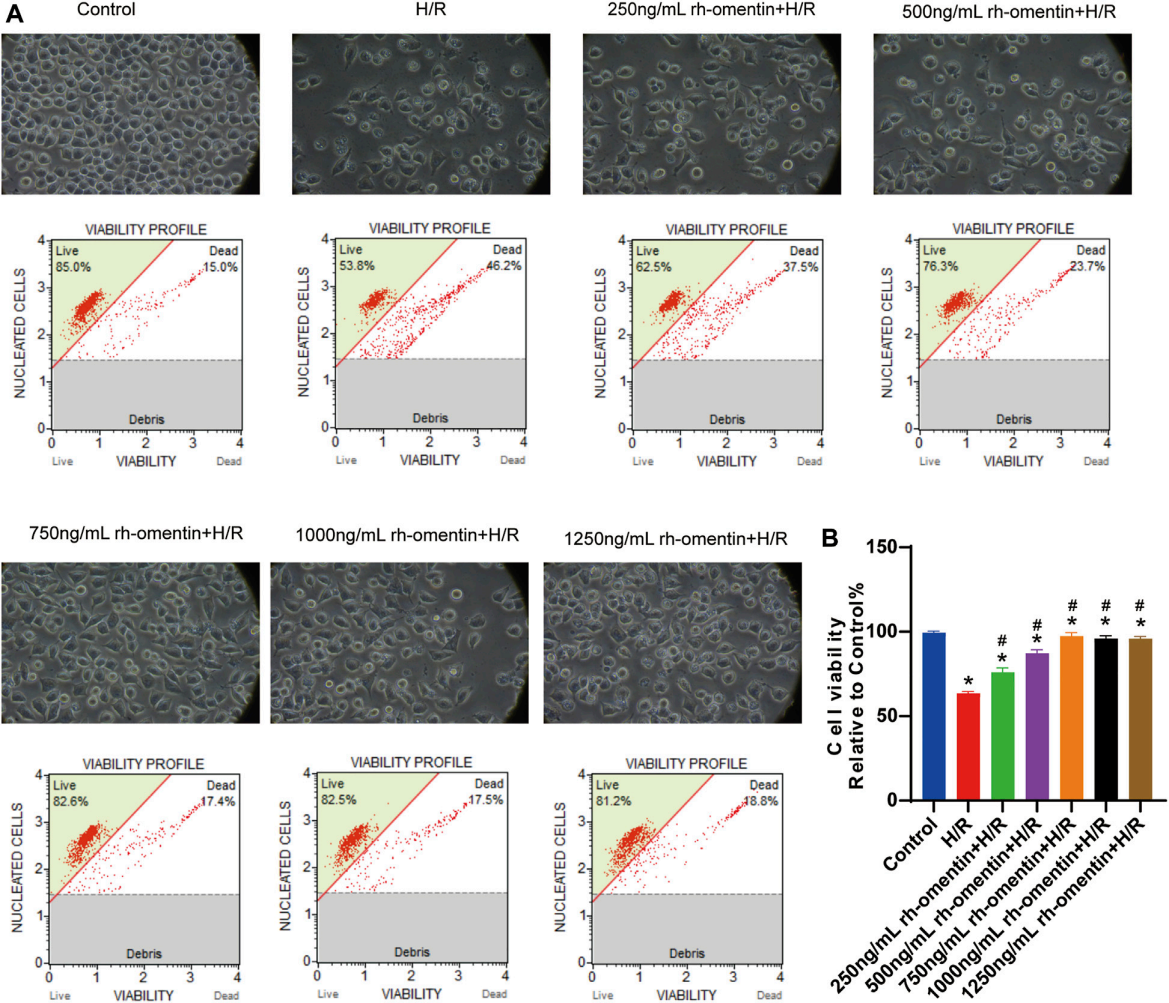
Rh-omentin protected N2a cells against H/R-induced injury. **(A)** N2a cells were treated with rh-omentin at the different concentrations and then exposed to H/R injury, followed by detection of cell viability. **(B)** Statistical graph of cell viability. Error bars show the standard deviation for *n* = 6 measurements from representative experiments. *p < 0.05 vs the Control group; ^#^ *p* < 0.05 vs. the H/R group.

As shown in Figures 3A,B, cell viability decreased significantly to more than 40% (vs. the control group, *p* < 0.05). Additionally, cell shrinkage and debris, and considerable morphological changes were observed. However, pretreatment with the concentrations of rh-omentin for 3 h markedly attenuated H/R-induced N2a cell death (vs. the H/R group, *p* < 0.05), among which 750 ng/ml of rh-omentin showed the most significant improvement. Hence, the dose of 750 ng/ml will be used in the next experiments.

### Rh-Omentin Inhibited H/R-Induced LDH Release, Intracellular ROS Generation and Cellular Apoptosis

**FIGURE 4:**
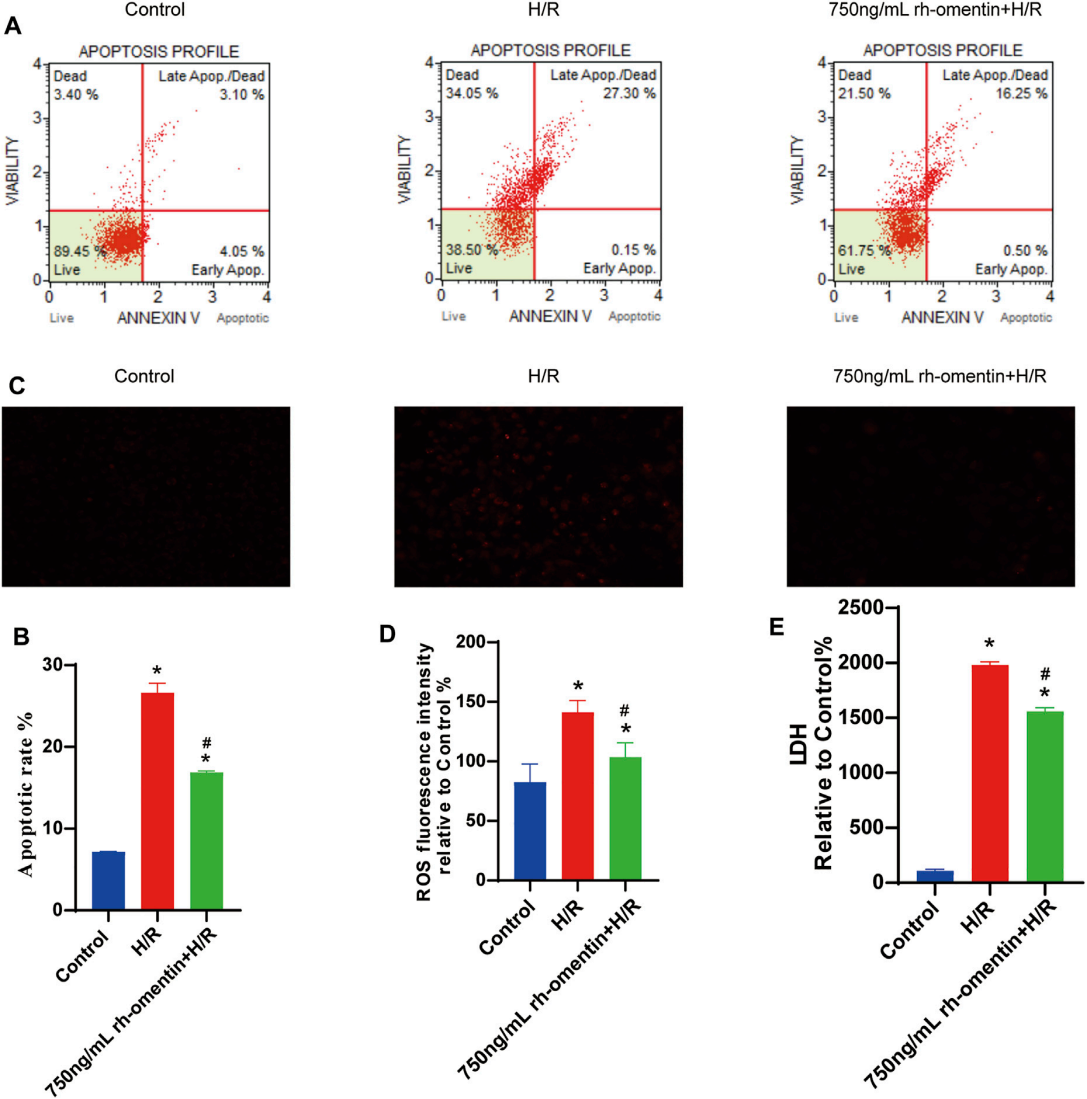
Rh-omentin inhibited H/R-induced LDH release, intracellular ROS generation and cellular apoptosis. **(A)** Apoptotic analysis of H/R-induced injury in N2a cells. **(B)** Statistical graph of apoptotic rates. **(C)** Intracellular ROS levels of H/R-induced injury in N2a cells. **(D)** Statistical graph of intracellular ROS levels. **(E)** LDH release of H/R-induced injury in N2a cells. Error bars show the standard deviation for *n* = 6 measurements from representative experiments. *p < 0.05 vs. the Control group; ^#^ *p* < 0.05 vs. the H/R group.

To monitor the effect of rh-omentin on intracellular ROS levels, the DHE staining assay was performed. H/R treatment obviously increased ROS generation in N2a cells (Figures 4C,D, vs. the control group, *p* < 0.05), while rh-omentin could effectively decreased the intracellular ROS generation (vs. the H/R group, *p* < 0.05). Similarly, compared with the H/R group, cell apoptotic rate and LDH level was significantly reduced in rh-omentin + H/R group (Figures 4A,B,E, *p* < 0.05).

### Effect of Rh-Omentin Treatment on Oxidative Stress Molecules, Apoptotic Molecules, and GAS6/Axl Signaling Molecules in N2a Cells Injured by H/R

**FIGURE 5:**
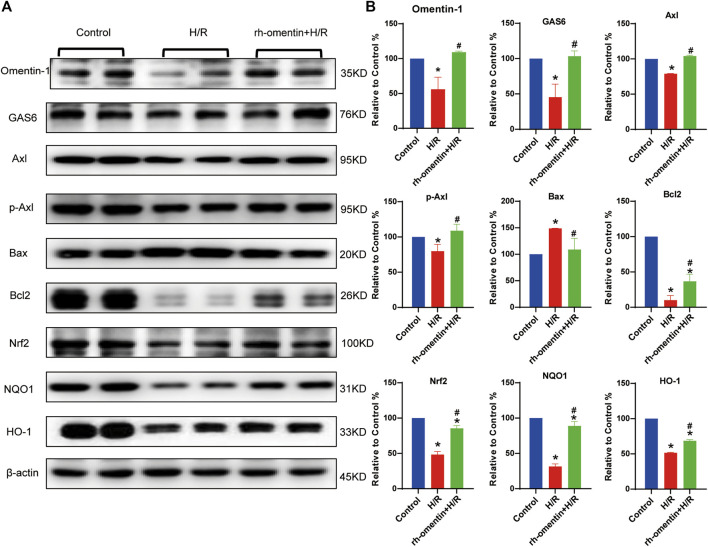
Effect of rh-omentin treatment on oxidative stress molecules, apoptotic molecules, and GAS6/Axl signaling molecules in N2a cells injured by H/R. **(A)** The representative images of Omentin-1, GAS6, Axl, p-Axl, NQO1, HO-1, Nrf2, Bax, and Bcl2 detected by Western blot. **(B)** Quantitative analysis of Western blot by normalizing to β-actin. Error bars show the standard deviation for *n* = 6 measurements from representative experiments. *p < 0.05 vs. the Control group; ^#^ *p* < 0.05 vs. the H/R group.

The expressions of omentin-1, GAS6, Axl, p-Axl, NQO1, HO-1, Nrf2, Bax, and Bcl2, were detected by western blot. Pretreatment with rh-omentin could significantly increase omentin-1, GAS6, Axl, p-Axl, NQO1, HO-1, Nrf2, Bcl2 expressions and decrease Bax expression. These results suggested that rh-omentin might be able to improve oxidative stress and apoptosis, and activate GAS6/Axl signaling pathways in N2a cells injured by H/R (Figures 5A,B, vs. the control group, *p* < 0.05).

### Effect of GAS6 siRNA and Rh-Omentin on the Cell Viability, ROS Generation, Cellular Apoptosis, and LDH Release in N2a Cells Injured by H/R

**FIGURE 6:**
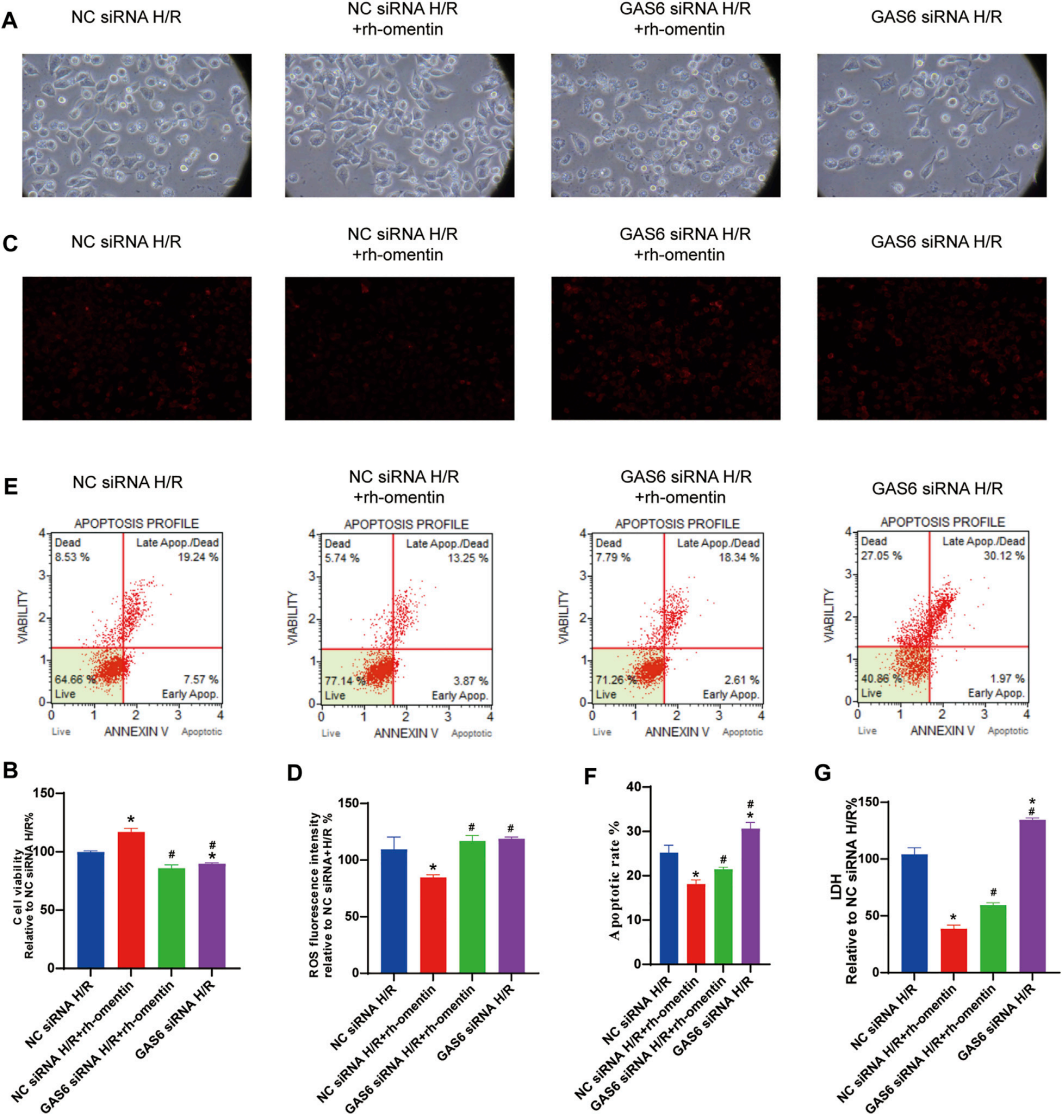
Effect of GAS6 siRNAs and rh-omentin on the cell viability, ROS generation, cellular apoptosis, and LDH release in N2a cells injured by H/R. **(A)** N2a cells was transfected with siRNA targeting GAS6 or a negative control siRNA (NC) for 24 h, exposed to 750 ng/ml rh-omentin for 3 h, and then exposed to H/R injury, followed by detection of cell viability. **(B)** Statistical graph of cell viability. **(C)** Intracellular ROS levels of H/R-induced injury in N2a cells. **(D)** Statistical graph of intracellular ROS levels. **(E)** Apoptotic analysis of H/R-induced injury in N2a cells. **(F)** Statistical graph of apoptotic rates. **(G)** LDH release of H/R-induced injury in N2a cells. Error bars show the standard deviation for *n* = 6 measurements from representative experiments. *p < 0.05 *vs.* the NC siRNA + H/R; ^#^ *p* < 0.05 *vs.* the NC siRNA + rh-omentin + H/R group.

To investigate the role of GAS6/Axl signaling in the protective effect of rh-omentin. The GAS6 siRNA was used in the next experiment. Firstly, the toxicity of siRNA silencing to N2a cells was detected by cell viability. As shown in Supplementary Figures 4A,B, siRNA treatments did not affect cell viability and cell morphology (vs. the control group, *p* < 0.05). Then, the effectiveness of GAS6 siRNA was evaluated by qRT-PCR. As shown in Supplementary Figure 4C, GAS6 siRNA reduced the mRNA level of GAS6 by about 70% (vs. the NC siRNA group, *p* < 0.05). After being treated with GAS6 siRNA for 24 h, N2a cells were treated with rh-omentin for 3 h and then exposed to H/R injury. Compared with the control siRNA + rh-omentin + H/R group, GAS6 siRNA significantly reversed the protective effect of rh-omentin on the cell viability, ROS generation, cellular apoptosis, and LDH release in N2a cells injured by H/R (Figures 6A–G, *p* < 0.05).

### Effect of GAS6 siRNA and Rh-Omentin on GAS6/Axl Signaling in N2a Cells Injured by H/R

**FIGURE 7:**
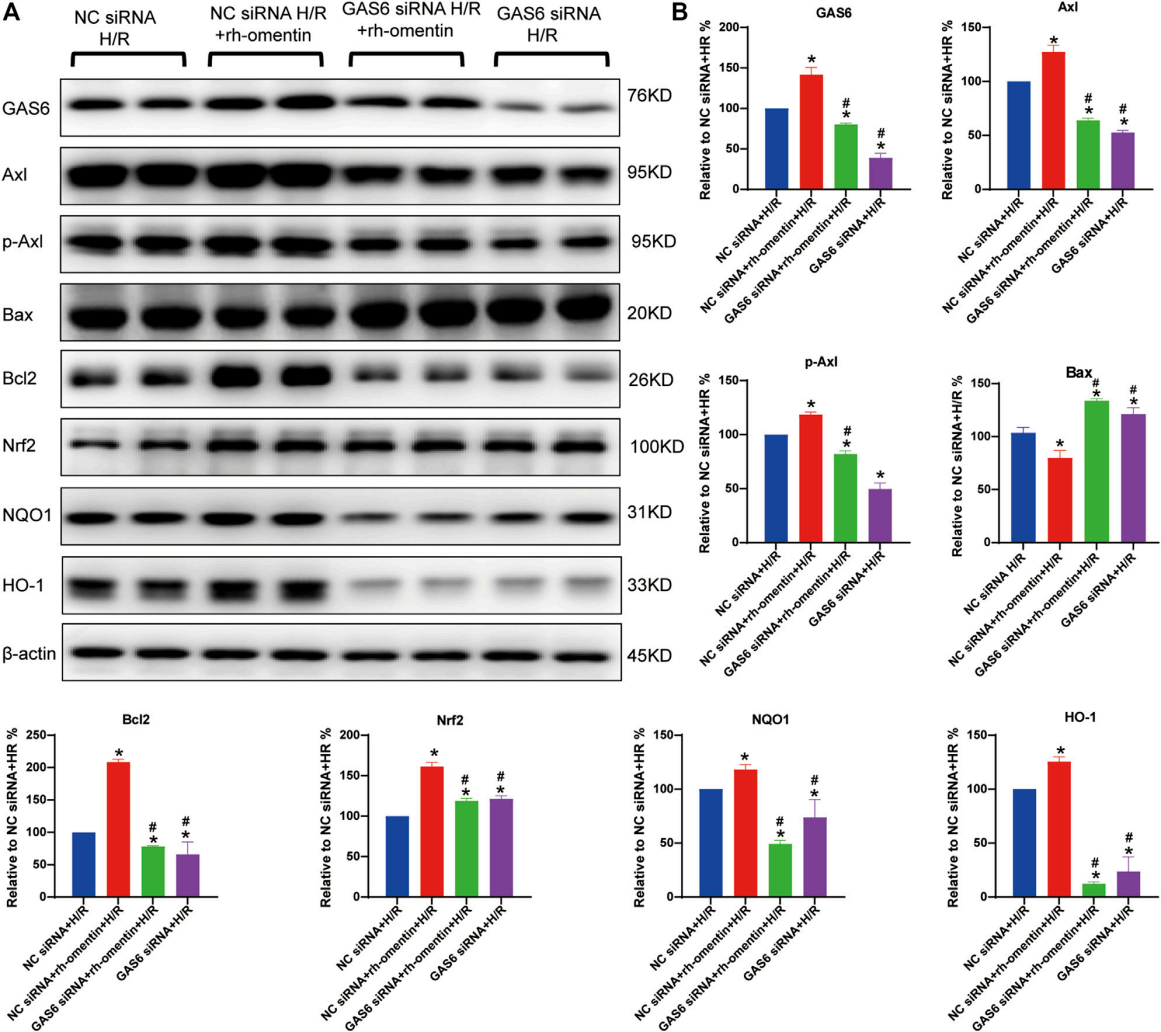
Effect of GAS6 siRNA and rh-omentin on GAS6/Axl signaling in N2a cells injured by H/R. **(A)** The representative images of GAS6, Axl, p-Axl, NQO1, HO-1, Nrf2, Bax, and Bcl2 detected by Western blot. **(B)** Quantitative analysis of Western blot by normalizing to β-actin. Error bars show the standard deviation for *n* = 6 measurements from representative experiments. *p < 0.05 *vs*. the Control siRNA + H/R; ^#^ *p* < 0.05 *vs.* the NC siRNA + rh-omentin + H/R group.

As shown in Figures 7A,B, rh-omentin treatment could obviously upregulate GAS6, Axl, p-Axl, NQO1, HO-1, Nrf2 and Bcl2 expressions and downregulate Bax expression (vs the NC siRNA + H/R group, *p* < 0.05). However, GAS6 siRNA treatment reversed these molecules mentioned above (vs. the NC siRNA + rh-omentin + H/R group, *p* < 0.05).

### Effect of Omentin-1 Overexpression on the Cell Viability, ROS Generation, Cellular Apoptosis, and LDH Release in N2a Cells Injured by H/R

**FIGURE 8:**
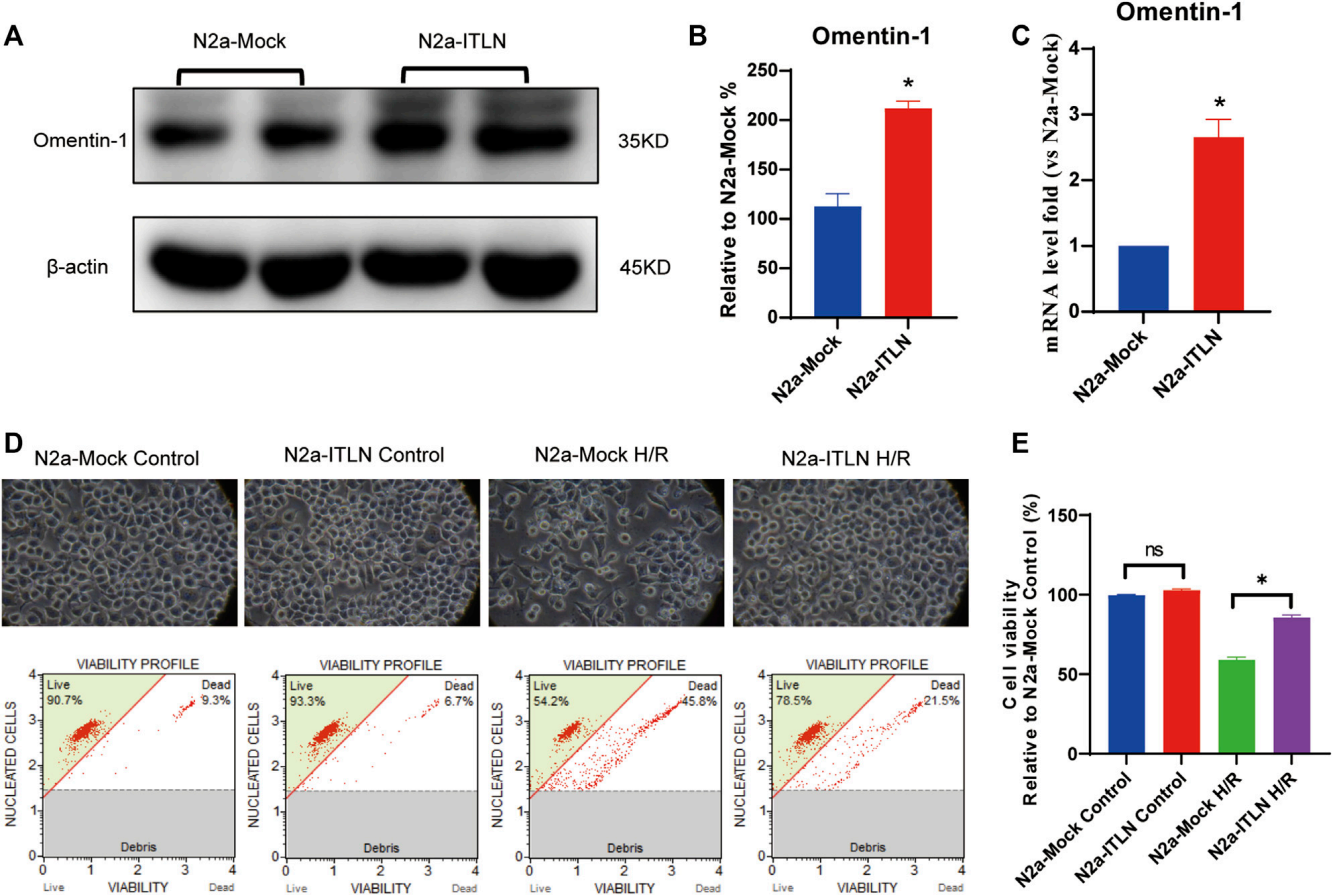
Omentin-1 overexpression protected N2a cells against H/R-induced injury. **(A)** The representative images of omentin-1 detected by Western blot. **(B)** Quantitative analysis of Western blot by normalizing to β-actin. **(C)** qRT–PCR analysis of omentin-1 mRNA expression normalized to the β-actin. **(D)** Toxicity of the omentin-1 overexpression in N2a cells (Control or H/R treated). **(E)** Statistical graph of cell viability. ITLN = Omentin-1. Error bars show the standard deviation for *n* = 6 measurements from representative experiments. *p < 0.05 vs. the N2a-Mock group; ns, non-signifcant.

**FIGURE 9:**
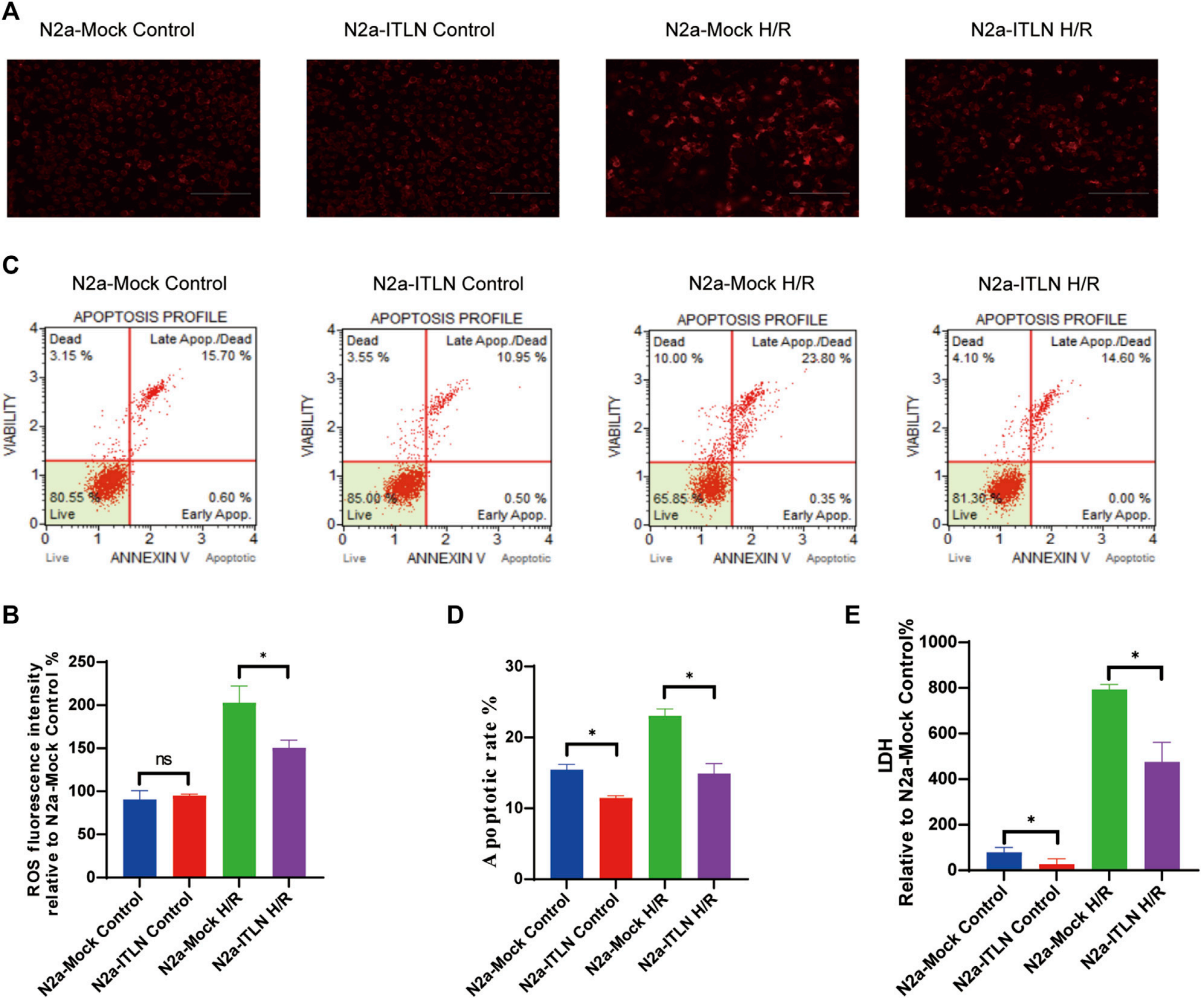
Omentin-1 overexpression inhibited H/R-induced LDH release, intracellular ROS generation, and cellular apoptosis. **(A)** Intracellular ROS levels of H/R-induced injury in lentivirus-infected N2a cells. **(B)** Statistical graph of intracellular ROS levels. **(C)** Apoptotic analysis of H/R-induced injury in lentivirus-infected N2a cells. **(D)** Statistical graph of apoptotic rates. **(E)** LDH release of H/R-induced injury in lentivirus-infected N2a cells. ITLN = Omentin-1. Error bars show the standard deviation for *n* = 6 measurements from representative experiments. *p < 0.05 vs. the N2a-Mock group; ns, non-significant.

As shown in Figures 8A–C a plenti-UCOE-SFFV vector targeting omentin-1 was constructed. Western blot and qRT–PCR were used to identify overexpressed cells. And the plenti-UCOE-SFFV-omentin-1 treatment was not toxic to N2a cells but effectively increased omentin-1 mRNA and protein expression (Figures 8D,E vs. the N2a-Mock group, *p* < 0.05). Compared with the N2a-Mock + H/R group, omentin-1 overexpression significantly increased cell viability, while decreased apoptotic rate, LDH release, and ROS production in N2a cells injured by H/R (Figures 9A–E *p* < 0.05).

## Discussion

**FIGURE 10:**
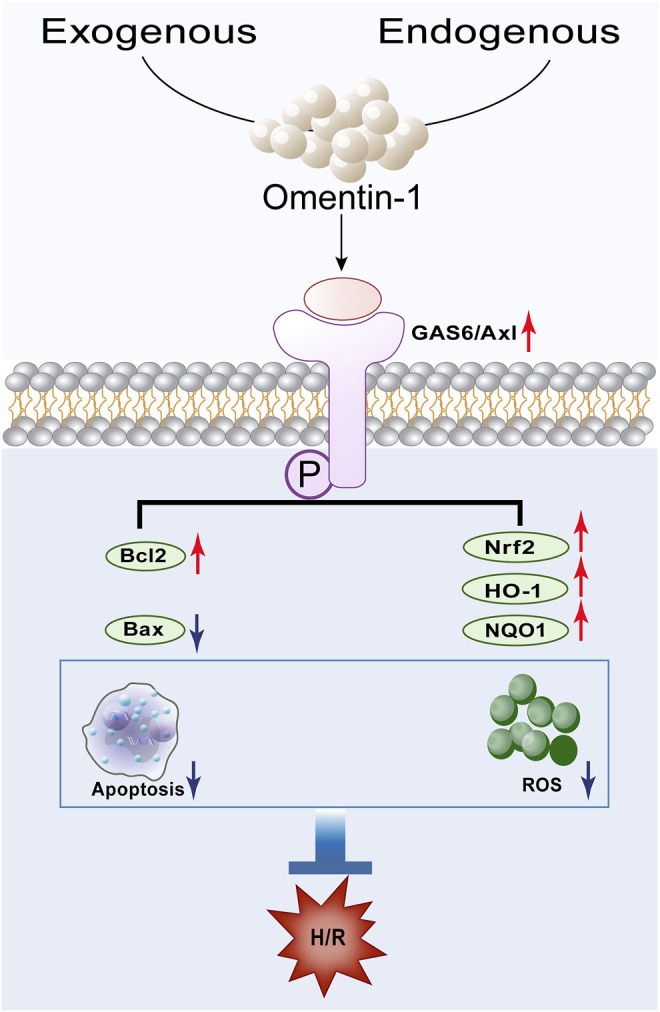
Schematic depicting the proposed mechanisms by which omentin-1 protects against H/R-induced injury by activating the GAS6/Axl signaling pathway.

Cerebral ischemia-reperfusion causes 60–70% of strokes and leads to brain damage through a series of complex pathophysiological events, which is characterized by neuronal death and subsequent neurological dysfunction (Owen and Sunram-Lea, 2011). Cerebral ischemia is proven to be one of the leading causes of death worldwide, but current treatment methods are still not fully satisfactory to reduce the severity of this disease (Iadecola et al., 2020). Omentin-1 was originally found in Paneth cells of the small intestine and is considered to be the intestinal lactoferrin receptor (Suzuki et al., 2001). Omentin-1 is demonstrated to exhibit remarkable therapeutic effect on multiple diseases, such as cardiovascular diseases and various metabolic related diseases (Narumi et al., 2014; Du et al., 2016; Leandro et al., 2021). Multiple pharmacological activities, such as anti-oxidative, anti-inflammatory, anti-apoptotic, and anti-microbial effect, are responsible for its extensive therapeutic effect (Kaho et al., 2016; Yin et al., 2017; Naibing et al., 2019). Importantly, our previous study has found that omentin-1 act as a novel regulator of vascular and anti-apoptotic response in cerebral ischemia (Gu et al., 2017). In this study, omentin-1 exerted neuroprotective effect against H/R injury in neuroblastoma N2a cells through activation of the GAS6/Axl signaling pathway, which is accompanied by alleviation of apoptosis and oxidative stress (Figure 10).

Oxidative stress is induced by the imbalance between oxidation and anti-oxidation in an organism, leading to the excessive production of free radicals and accounting for the pathogenesis of many diseases, such as stroke, cerebral IRI, Alzheimer’s disease and so on (Rossetti et al., 2020; Dionísio et al., 2021). Nrf2 is an extremely critical transcription factor whose main role is to induce endogenous antioxidant enzymes to resist on oxidative stress. Meanwhile, Nrf2 is regarded as a regulator to maintain redox homeostasis in body. HO-1 and NQO1 have been proved to be the downstream target genes of Nrf2 (Kahroba et al., 2021). Pan et al. (2017) showed that astaxanthin pretreatment significantly increased the expression of Nrf2, HO-1, and NQO1 mRNA in a cerebral ischemia rat model evidencing a protective effect against brain injury. In this study, H/R injury increased ROS production, and decreased Nrf2, HO-1 and NQO1 expressions significantly, while rh-omentin treatment remarkably decreased ROS production and increased the expressions of Nrf2, HO-1 and NQO1, suggesting that omentin-1 has anti-oxidative effect on H/R injury.

Apoptosis is a gene-controlled autonomous cell death process, also known as programmed cell death under physiological conditions. It is one of the mechanisms to maintain the stability of the internal environment (Carneiro and El-Deiry, 2020). Evidence shows that neuronal apoptosis is the basic form of delayed neuronal death after cerebral IR (Liu et al., 2020). Anti-apoptotic Bcl2 and pro-apoptotic Bax were determined to play key roles in cell survival or death (Pihán et al., 2017). Gao et al., confirmed that H/R injury could activate the expressions of Bax and caspase-3, reduce the expressions of Bcl-2 and surviving in N2a cells. Resveratrol treatment reversed the levels of anti-apoptotic proteins and reduced the pro-apoptotic factors in the presence of H/R injury (Gao et al., 2019). Moreover, our study found that mangiferin treatment remarkably decreased the cell apoptotic rate and increased the antiapoptosis molecule Bcl2 induced by H/R injury (Chen et al., 2021). In this study, rh-omentin treatment could significantly reduce H/R-induced injury by inhibiting neuroblastoma apoptosis, as evidenced by remarkably decreased the cell apoptotic rate, Bax expression and increased Bcl2 expression. These results suggested that the neuroprotective role of rh-omentin may be mediated through apoptotic signaling pathways.

GAS6 is a vitamin K-dependent protein, first identified in murine fibroblasts in 1988. The biological functions of GAS6 are mediated by the interaction with TAM receptors, including cell survival and proliferation, immune regulation and apoptosis (van der Meer et al., 2014). Notably, the neuroprotective role of GAS6/Axl axis has been identified in many studies. Tong et al. (2017) found that in an intracerebral hemorrhage mouse model, recombinant GAS6 administration can facilitate immune restoration, alleviate brain edema and improve neurobehavioral performance, probably by enhancing Axl phosphorylation. In addition, in the rat model of stroke, recombinant GAS6 increases the expression of Axl and improve the short-term and long-term effect after middle cerebral artery occlusion, suggesting its profound neuroprotective effect (Wu et al., 2018). Importantly, a recent study found that omentin-1 promotes vascular remodeling in the ischemic state to improve endothelial function, reduce infarct size, and reduce apoptosis (Yang and Gao, 2020). In this study, rh-omentin treatment remarkably upregulated the expressions of omentin-1, GAS6, Axl, p-Axl. Moreover, GAS6 siRNA significantly reversed the effect of rh-omentin against cell viability, apoptotic rate, LDH release, and ROS production, and upregulation of omentin-1, GAS6, Axl, p-Axl, Nrf2, HO-1, and NQO1 in H/R-injured N2a cells. These results suggested that the neuroprotective role of omentin-1 on H/R-induced injury are mediated by GAS6/Axl signaling.

At present, the specific receptor of omentin-1 has not been identified. Multiple studied confirmed that the signal transduction of omentin-1 is not via a specific protein receptor. Current studies confirm that omentin-1 may play a protective role through the following mechanisms: 1) Omentin-1 may inhibit inflammation, directly or indirectly, thereby decreasing the formation and rupture of unstable atherosclerotic plaques and preventing cerebrovascular accidents. 2) Omentin-1 also promotes vascular remodeling in the ischemic state to improve endothelial function, reduce infarct size, and reduce apoptosis. 3) Omentin-1 inhibits alkaline phosphatase activity and osteocalcin production, thereby inhibiting the differentiation of vascular smooth muscle cells into osteoblast-like cells to prevent vascular calcification. 4) Omentin-1 promotes endothelial release of bioactive substances, regulates endothelial function, and improves vasomotor function. 5) Omentin-1 inhibits oxidative stress in vascular smooth muscle cells, exerts an anti-atherosclerosis role, and inhibits oxidative stress in mitochondria, thereby decreasing cytotoxicity and exerting a neuroprotective role (Yang and Gao, 2020).

Omentin-1, as novel adipocytokine, plays vital roles in the maintenance of body metabolism and insulin sensitivity, and has anti-atherosclerotic, anti-inflammatory, and cardiovascular protective effects via AMPK/Akt/NF-κB/MAPK (ERK, JNK, and p38) signaling (Watanabe et al., 2017). Clinical studies have indicated the usage of circulating omentin-1 as a biomarker of obesity, metabolic disorders including insulin resistance, diabetes, and metabolic syndrome, and atherosclerotic cardiovascular diseases (Watanabe et al., 2017). These results suggest that omentin-1 plays an important role in maintaining metabolism. Importantly, a recent study found that omentin-1 promotes vascular remodeling in the ischemic state to improve endothelial function, reduce infarct size, and reduce apoptosis (Yang and Gao, 2020). However, the exact metabolic effects of omentin-1 in ischemia need further study.

Taken together, omentin-1 protects against H/R-induced injury through activation of the GAS6/Axl signaling pathway, which is accompanied by amelioration of oxidative stress and apoptosis (Figure 10). These findings may provide a theoretical basis for the application of omentin-1 as a potential neuroprotective drug candidate, which may be beneficial for the ischemic stroke patients in clinic.

## Data Availability

The original contributions presented in the study are included in the article/Supplementary Material, further inquiries can be directed to the corresponding authors.

## Supplementary Material

Supplementary Figure S1 Construction of an appropriate H/R injury model in neuroblastoma cells. **(A)** The morphology was observed at H (12 h)/ R (3, 6, and 9 h, respectively) in N2a cell. **(B)** Statistical graph of cell viability. Error bars show the standard deviation for *n* = 6 measurements from representative experiments. **P*<0.05 vs. the Control group.

Supplementary Figure S2 The expression of GAS6 in N2a cells injured by H/R. **(A)** The representative image of GAS6 detected by Western blot. **(B)** Quantitative analysis of Western blot by normalizing to β-actin. **(C)** QRT–PCR analysis of Omentin-1 mRNA expression normalized to the β-actin mRNA. **(D)** QRT–PCR analysis of GAS6 mRNA expression normalized to the β-actin mRNA. Error bars show the standard deviation for *n* = 6 measurements from representative experiments. **P*<0.05 vs. the Control group.

Supplementary Figure S3 The expression of neuronal markers in N2a cells induced by serum deprivation or H/R treatment. **(A)** GAP43 expression in N2a cells induced by serum deprivation or H/R treatment detected by immunofluorescence. **(B)** TUBB3 expression in N2a cells induced by serum deprivation or H/R treatment detected by immunofluorescence. All experiments were performed in triplicate.

Supplementary Figure S4 The toxicity and effectiveness of siRNA silencing on N2a cells. **(A)** N2a cells were transfected with small interfering RNA (Negative Control siRNA or GAS6 siRNA) for 4 h, then incubated for 24 h. Cell morphology was observed under an inverted/phase contrast microscope, and images were obtained. **(B)** Statistical graph of cell viability. **(C)** QRT–PCR analysis of GAS6 mRNA expression normalized to the β-actin. Error bars show the standard deviation for *n* = 6 measurements from representative experiments. **P*<0.05 vs. the NC siRNA; ns, non-significant.
