# Supplementary material for: Neuroprotective Effects of Omentin-1 Against Cerebral Hypoxia/Reoxygenation Injury via Activating GAS6/Axl Signaling Pathway in Neuroblastoma Cells
Source: Front Cell Dev Biol. 2022 Jan 24;9:784035. doi: 10.3389/fcell.2021.784035 (PMC8818945; doi:10.3389/fcell.2021.784035)

Figure 1A

Control

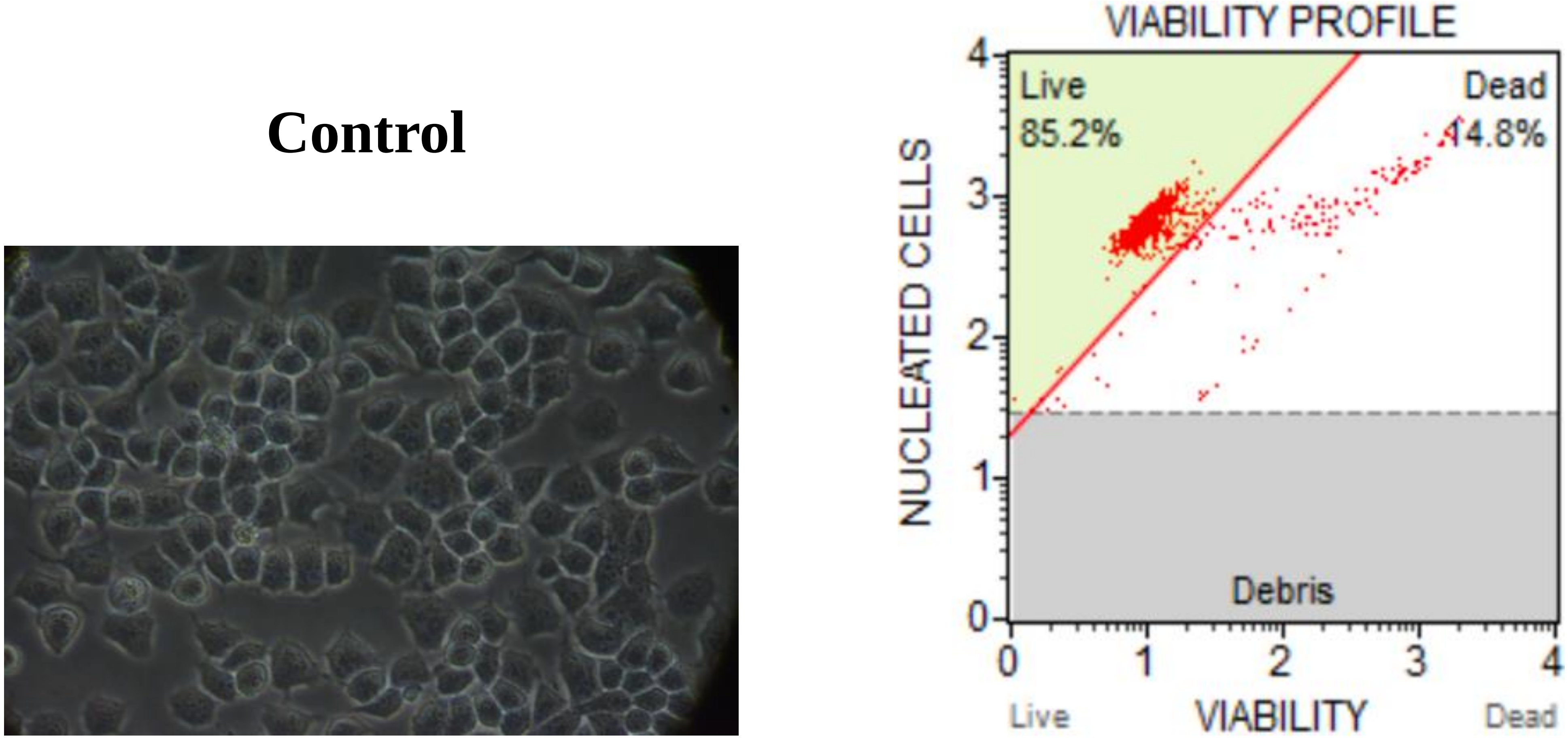

Figure 1A

H/R

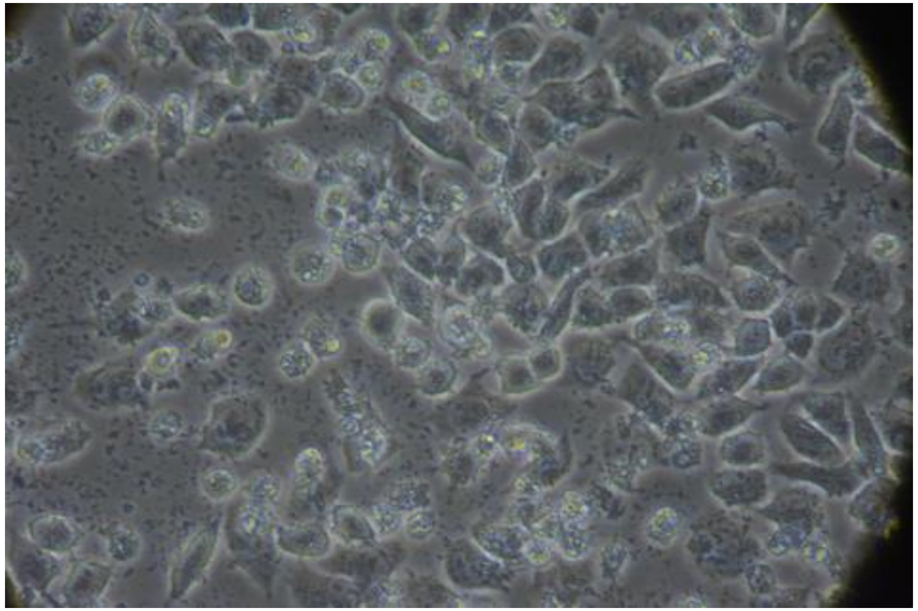

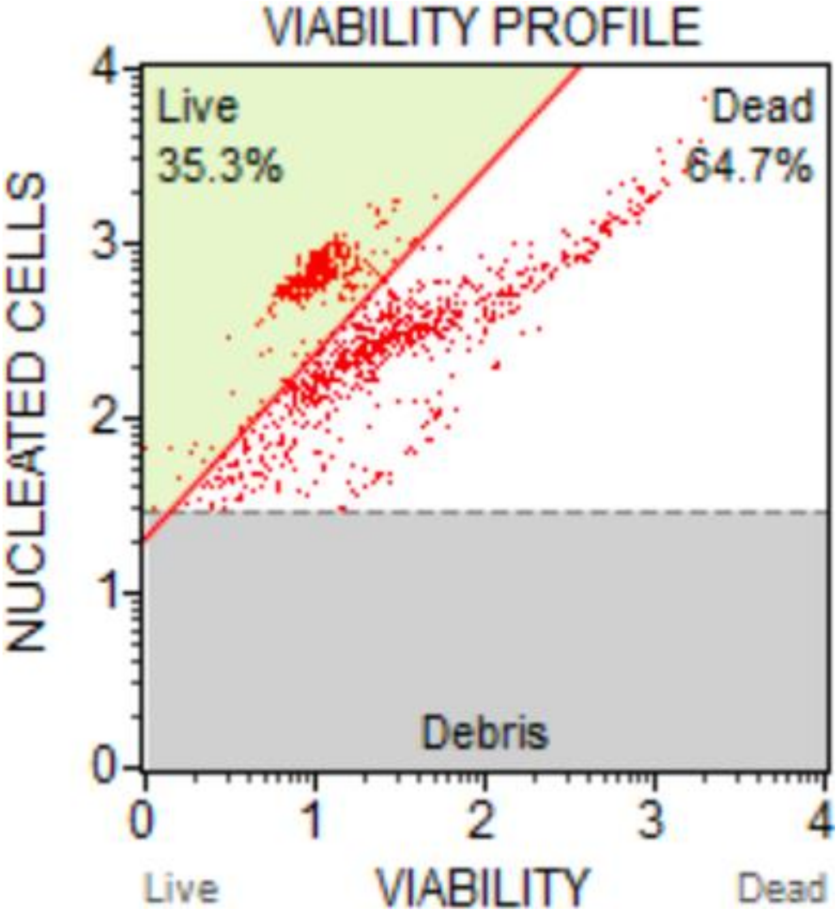
VIABILITY PROFILE
Live
35.3%
Dead
64.7%
NUCLEATED CELLS
Debris
0
1
2
3
4
0
1
2
3
4
Live
VIABILITY
Dead

Figure 1C

Control Omentin-1

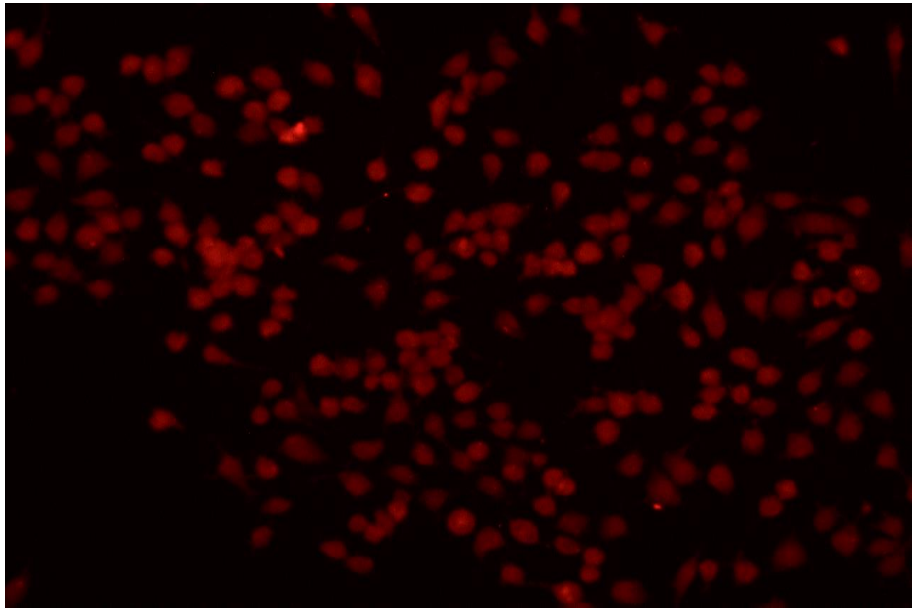

Figure 1C

H/R Omentin-1

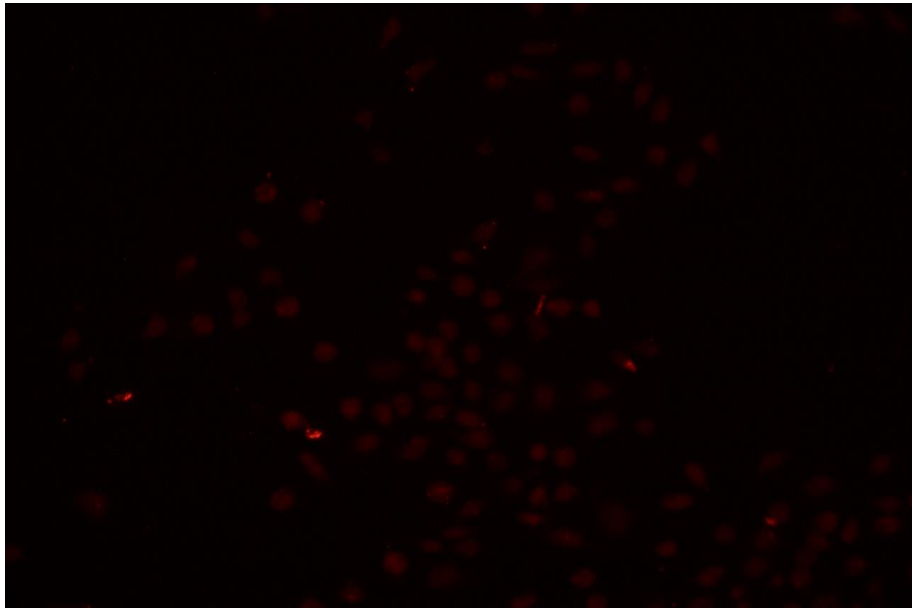

Figure 1C

Control DAPI

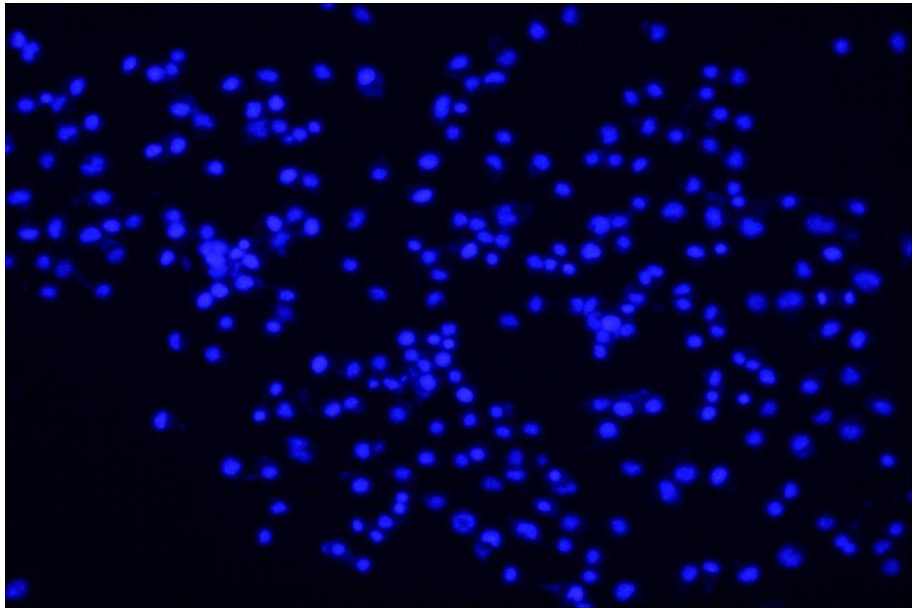

Figure 1C

H/R DAPI

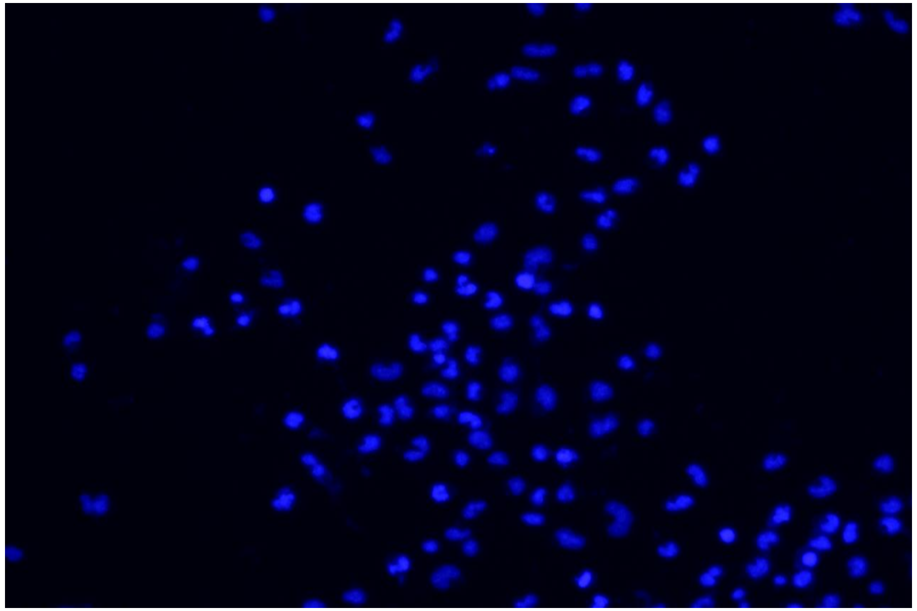

Figure 1C

Control Merge

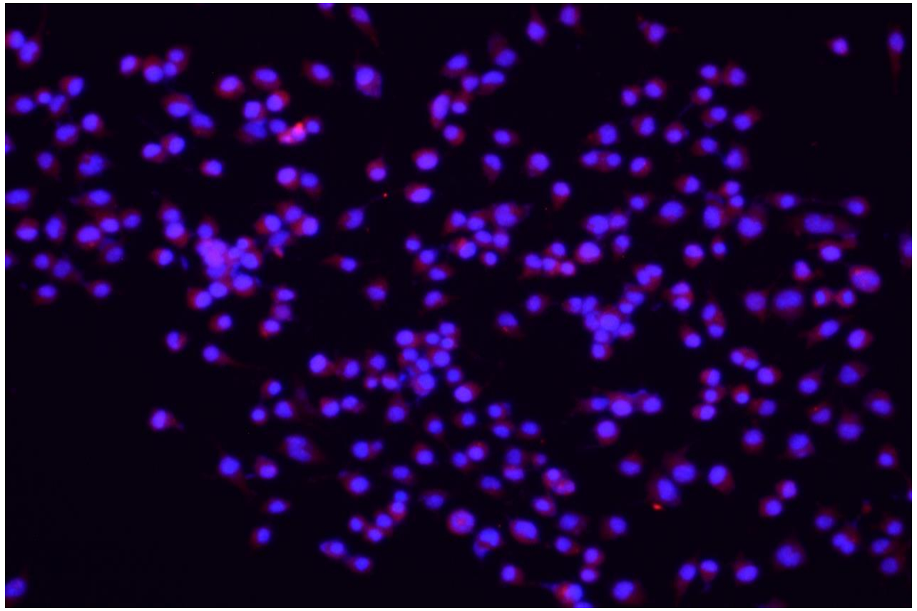

Figure 1C

H/R Merge

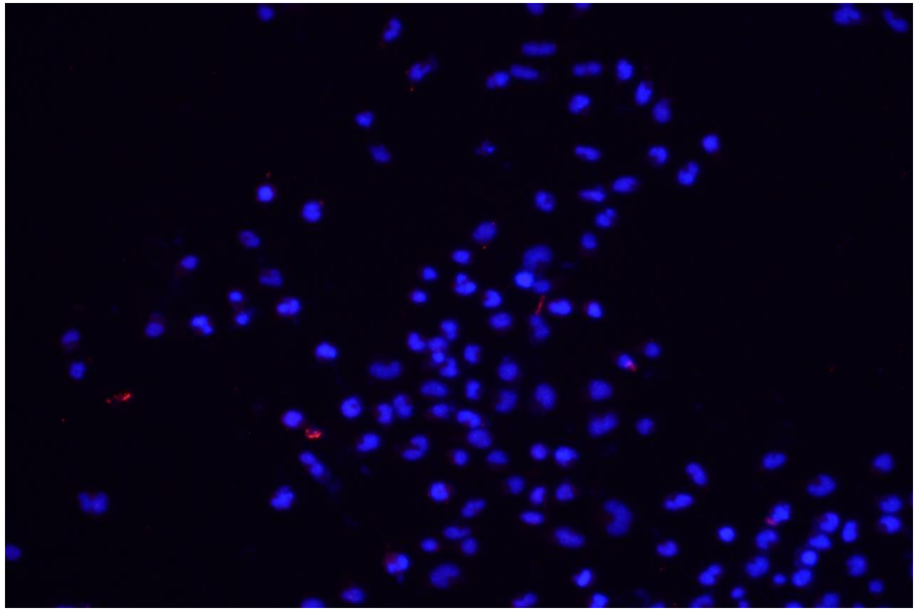

Figure 2A

Control

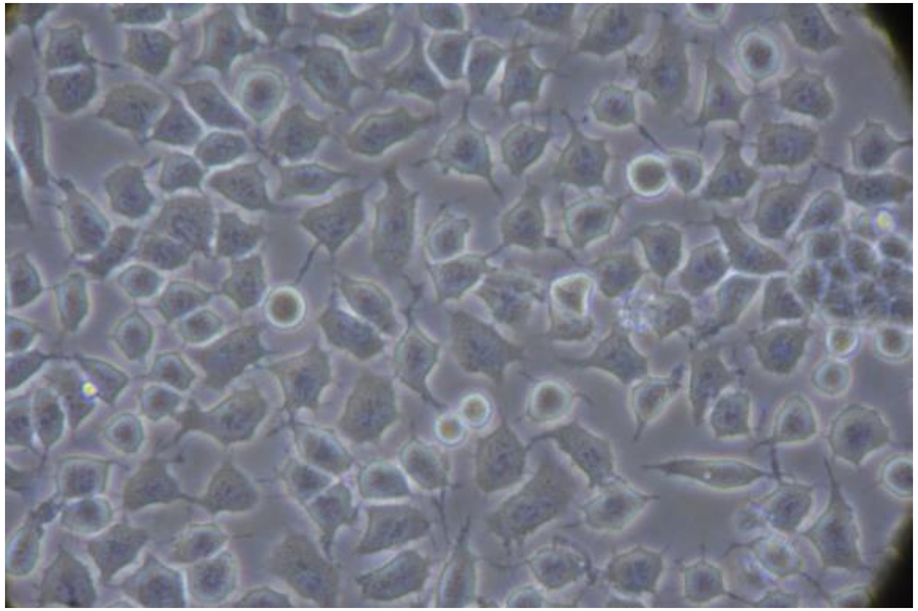

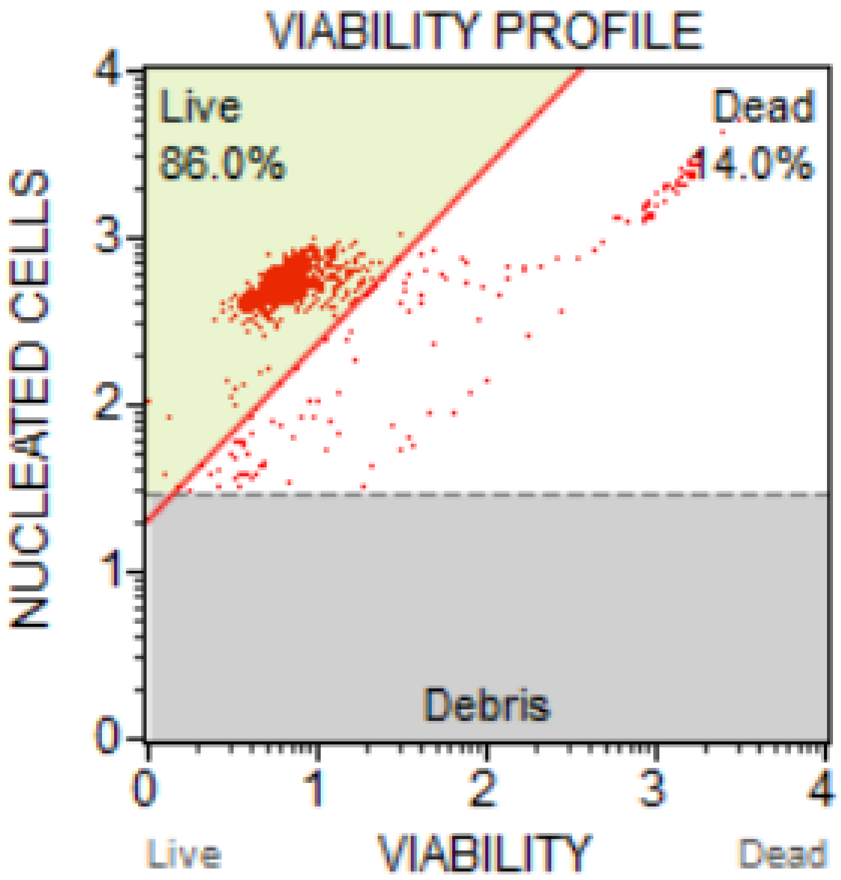

Figure 2A

250ng/ml rh-omentin

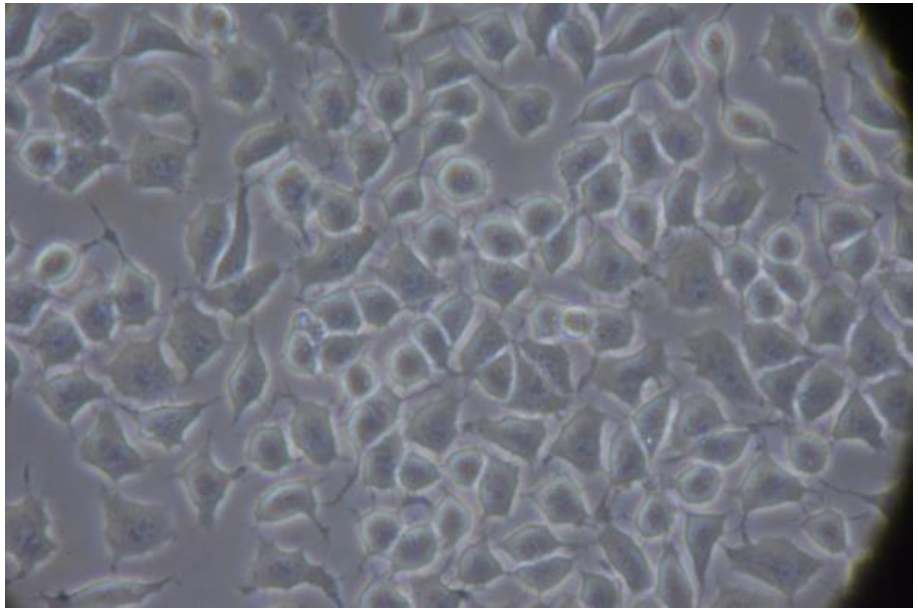

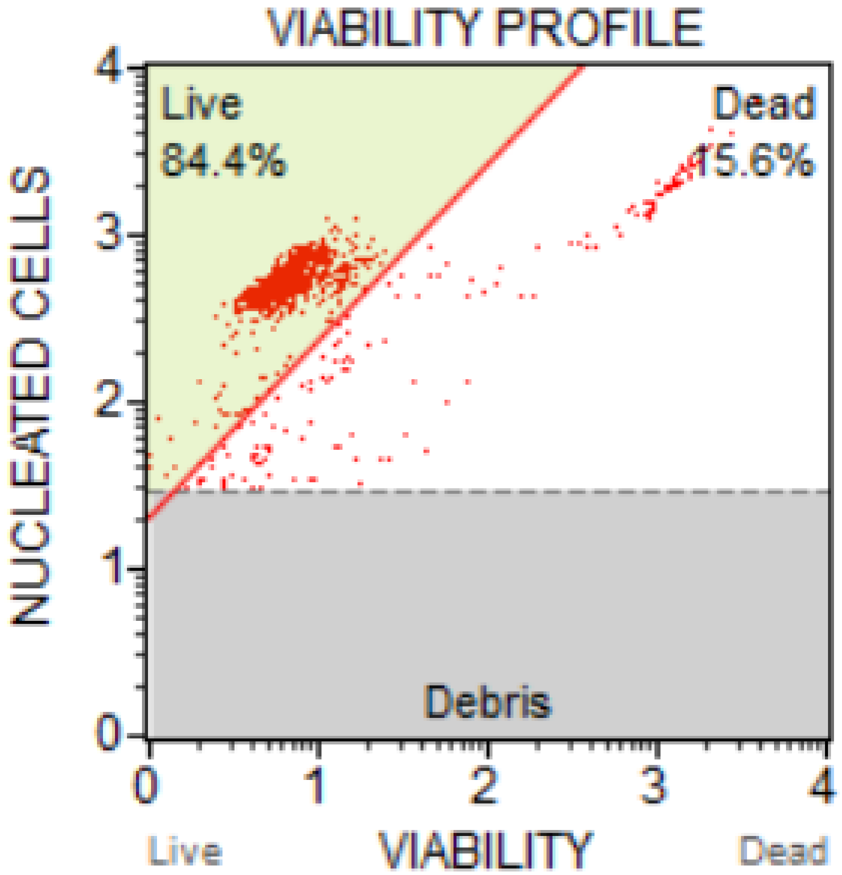

Figure 2A

500ng/ml rh-omentin

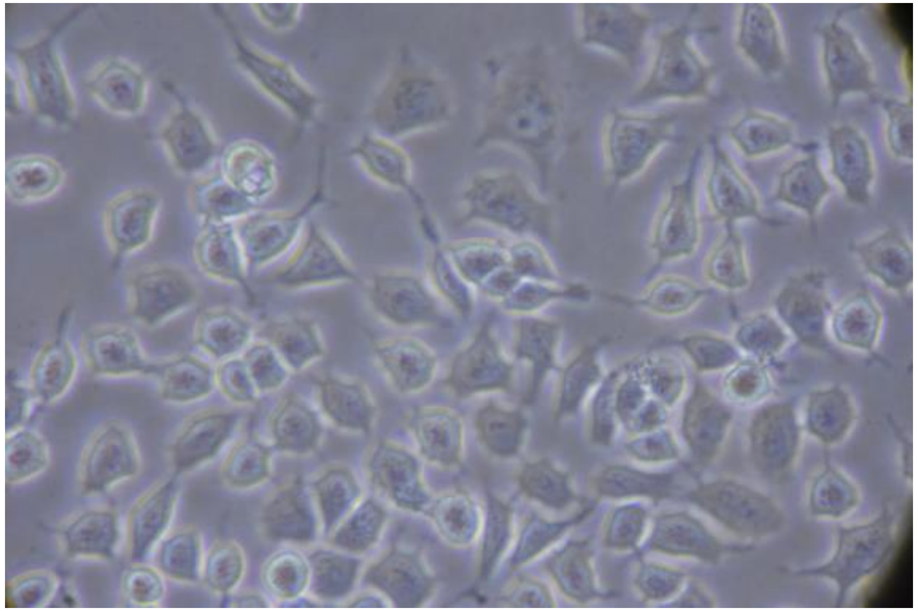

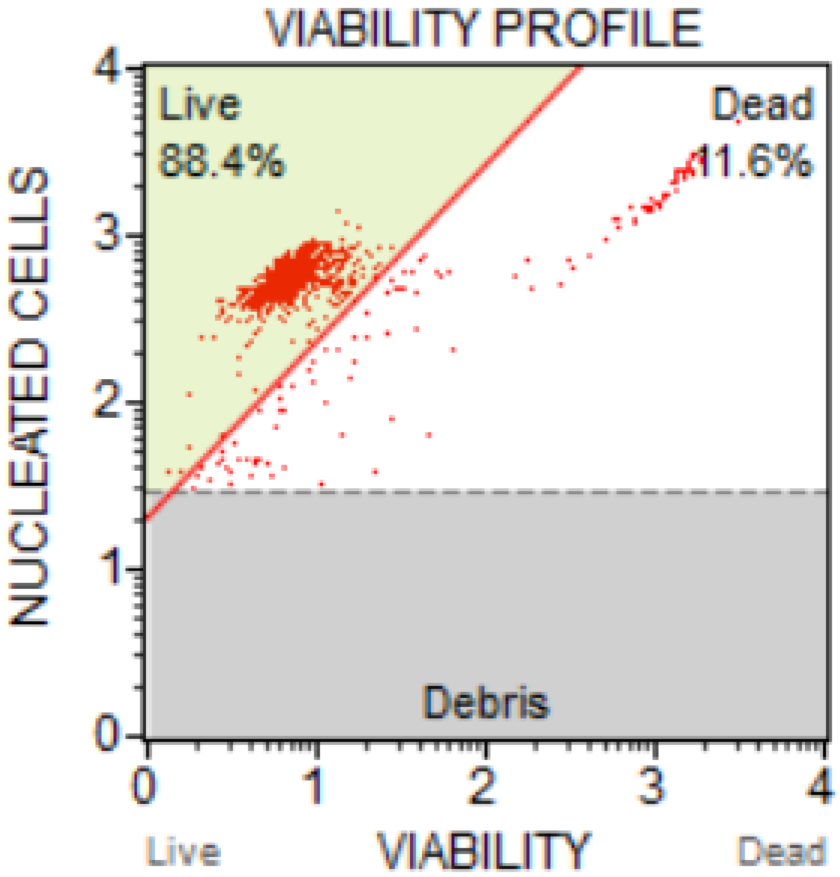

Figure 2A

750ng/ml rh-omentin

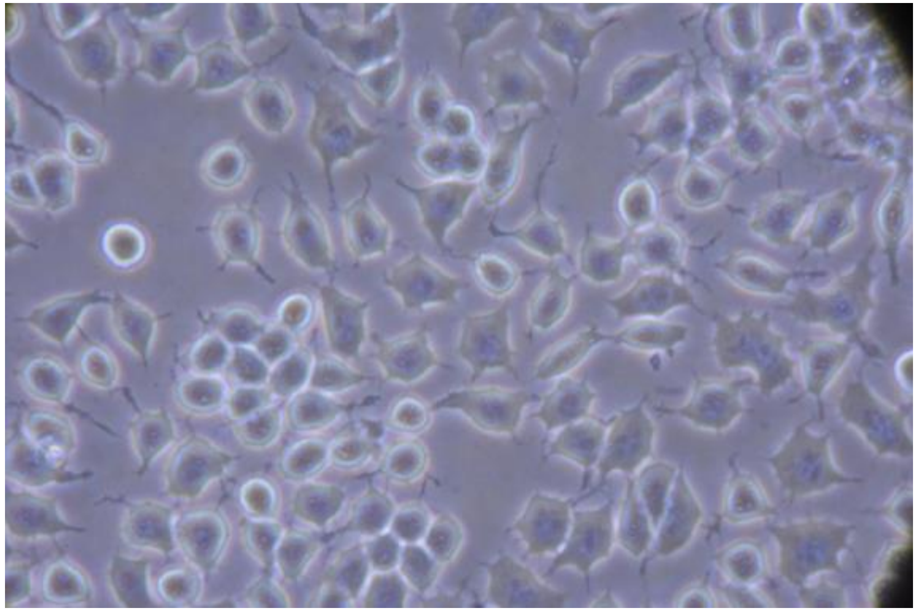

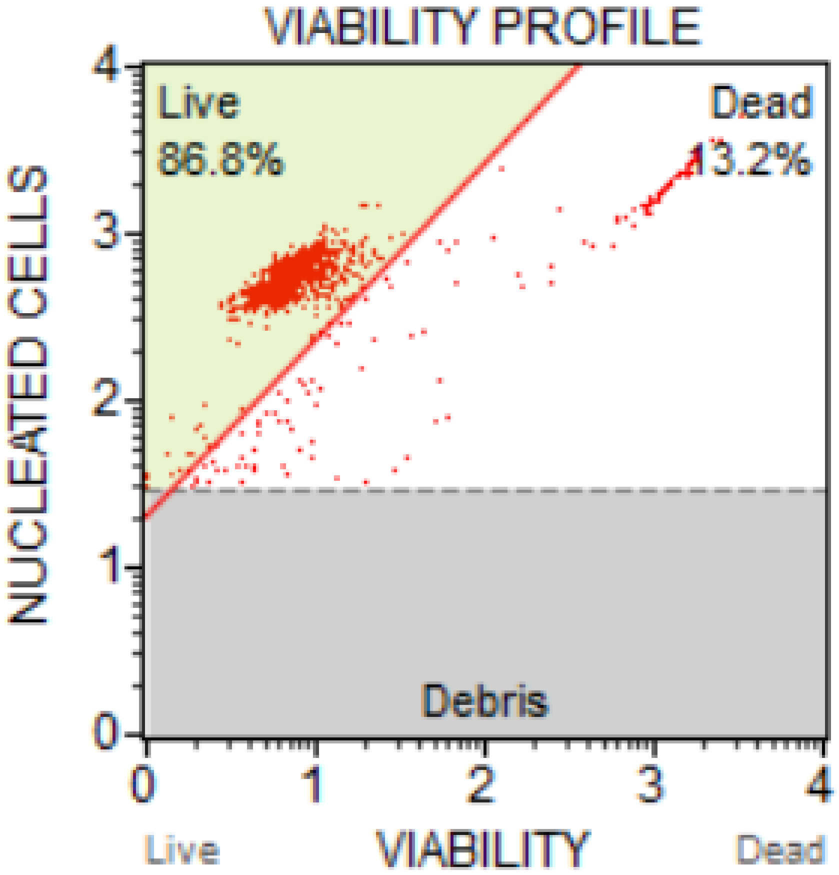

Figure 2A

1000ng/ml rh-omentin

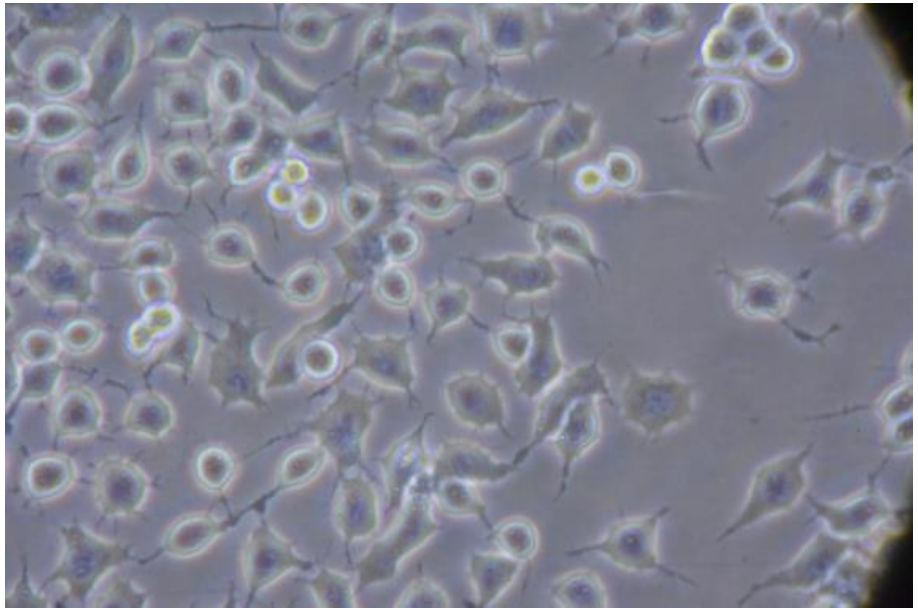

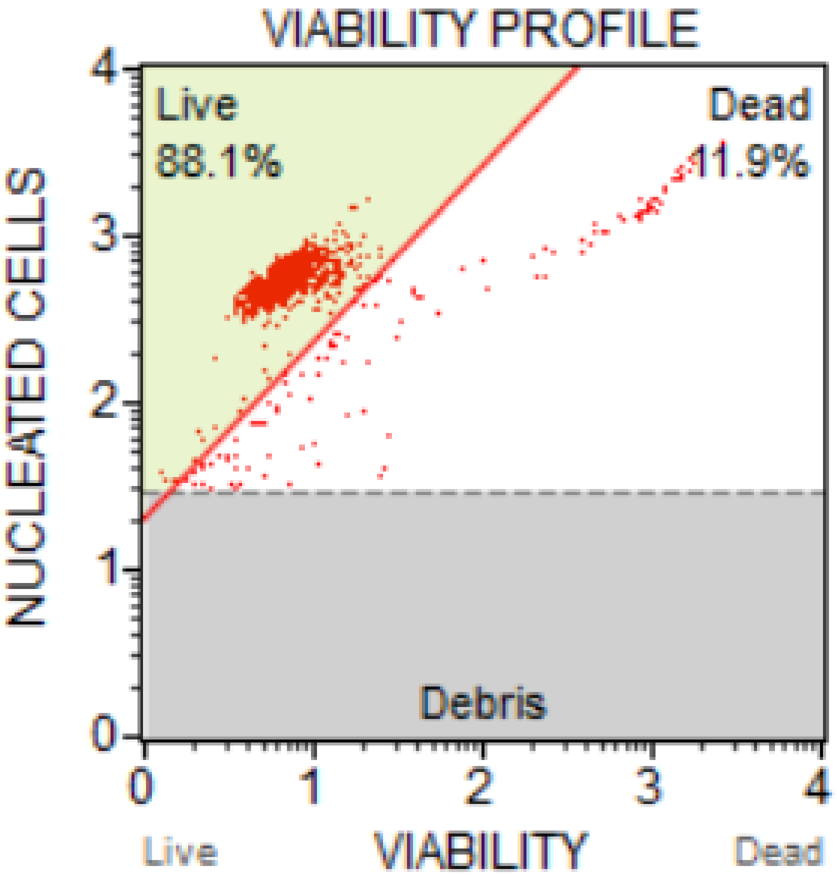

Figure 2A

1250ng/ml rh-omentin

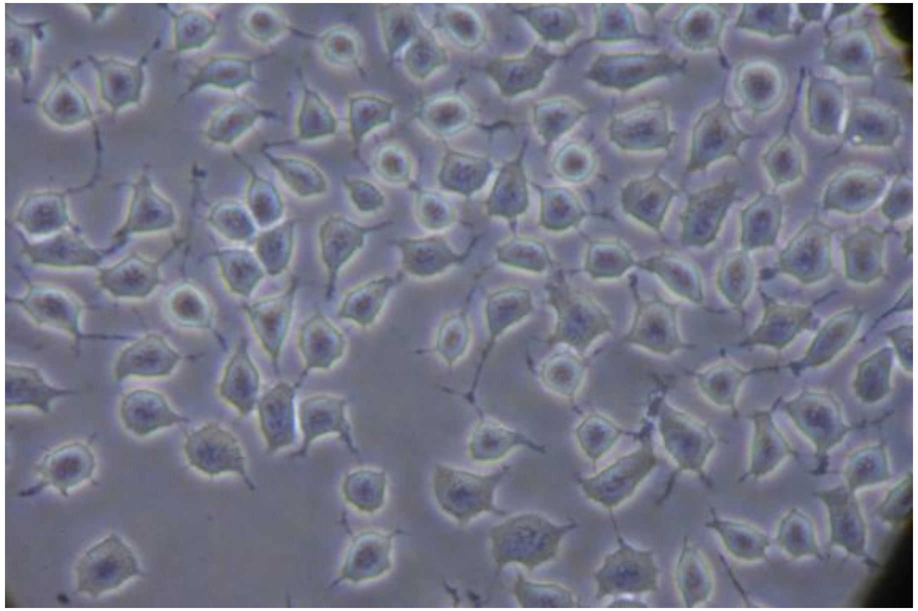

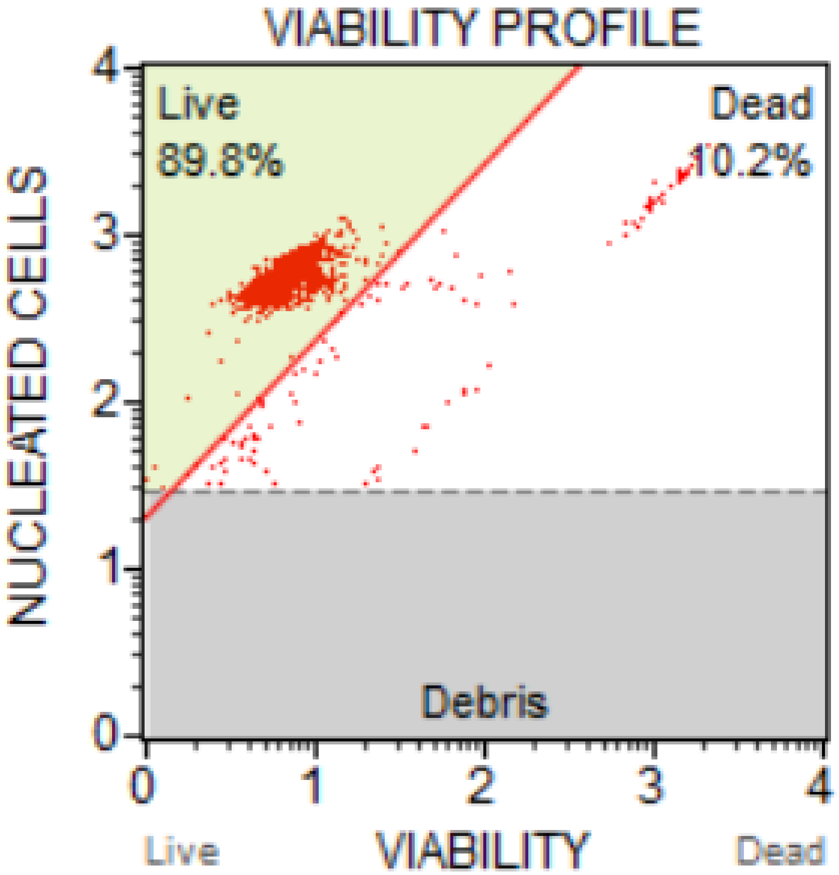

Figure 3A

Control

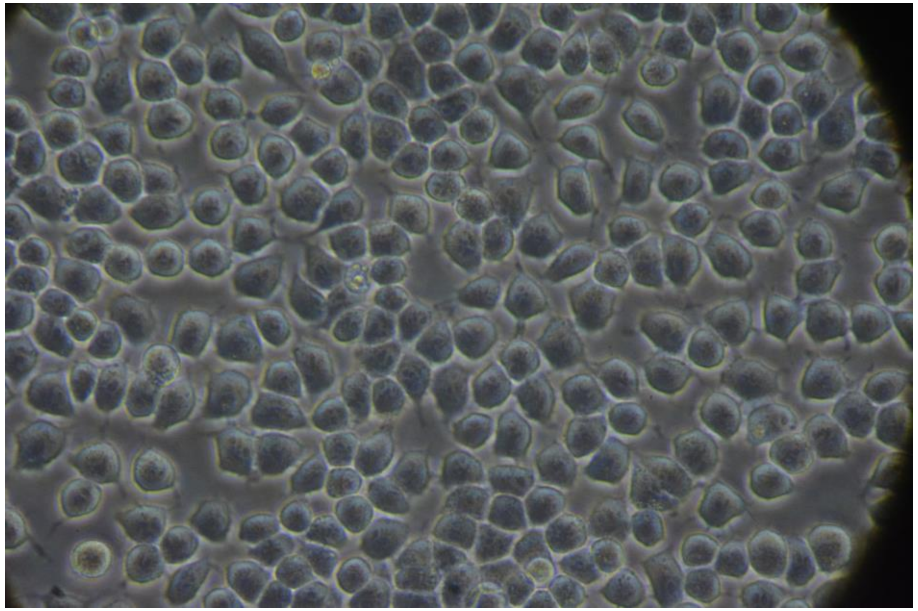

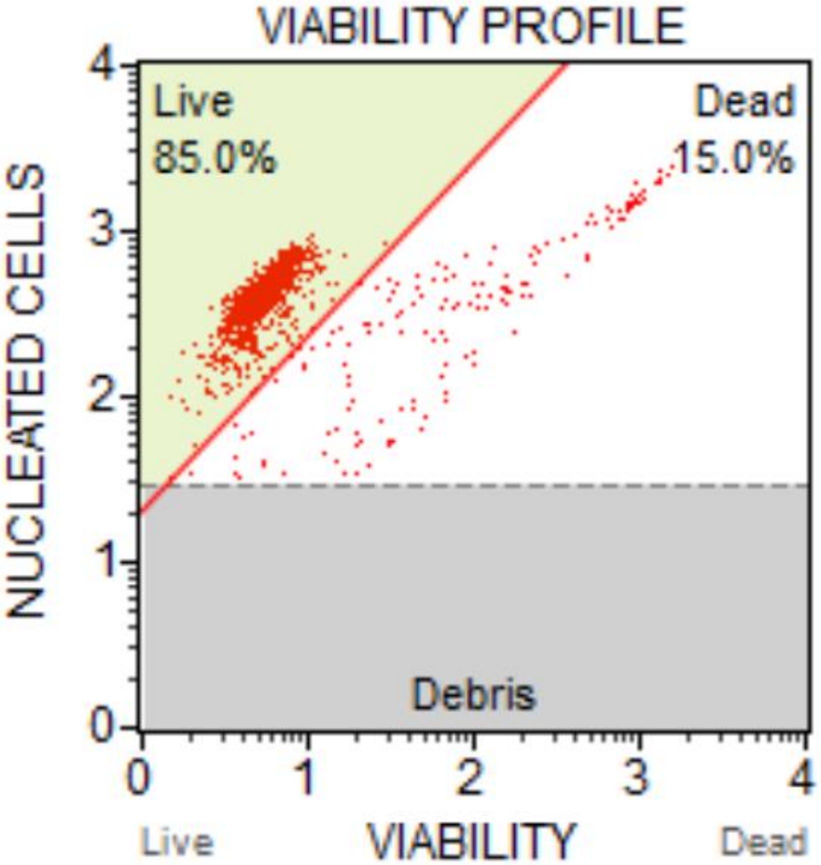

Figure 3A

H/R

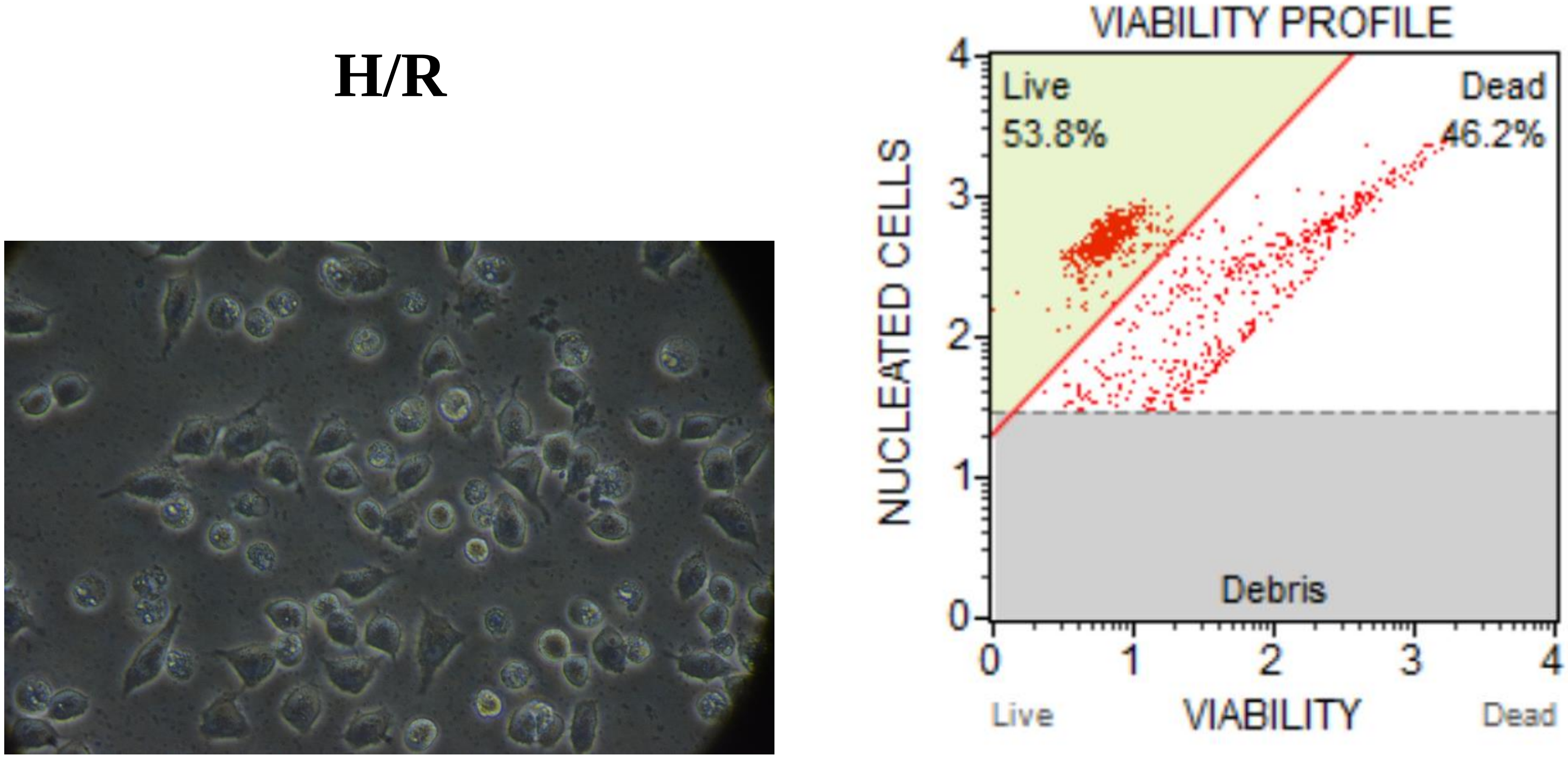
VIABILITY PROFILE
Live
53.8%
Dead
46.2%
Debris
NUCLEATED CELLS
VIABILITY
Live
Dead
0
1
2
3
4

Figure 3A

250ng/ml rh-omentin + H/R

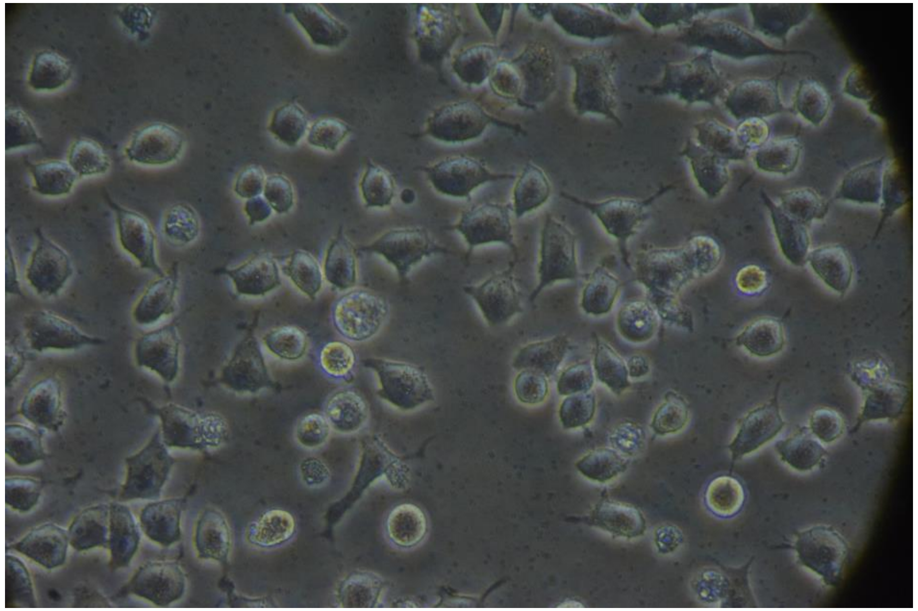

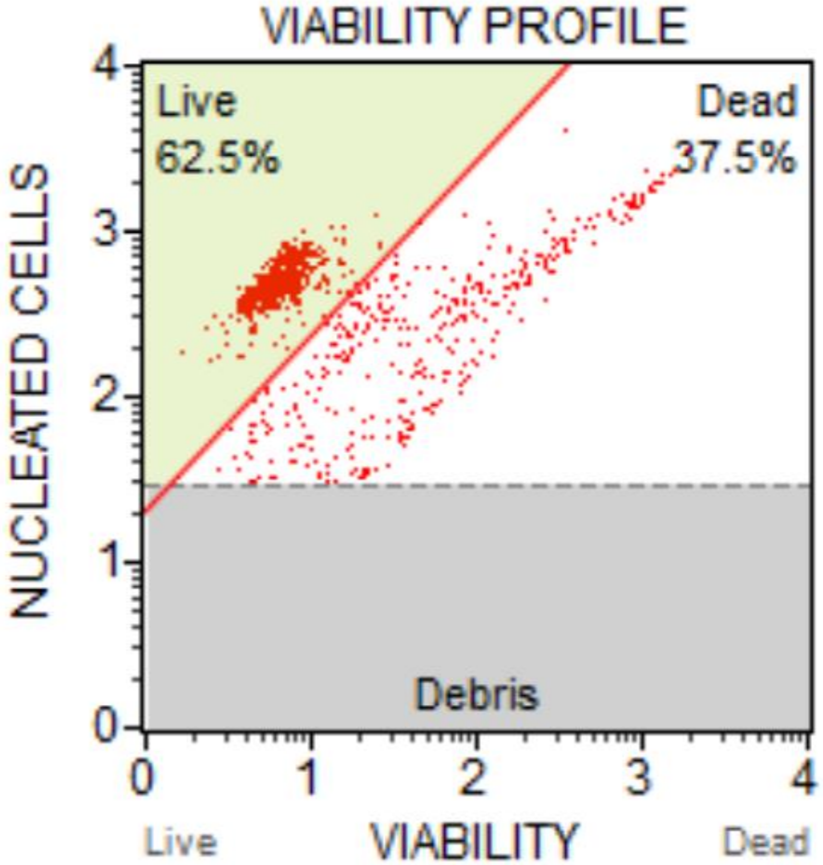

Figure 3A

500ng/ml rh-omentin + H/R

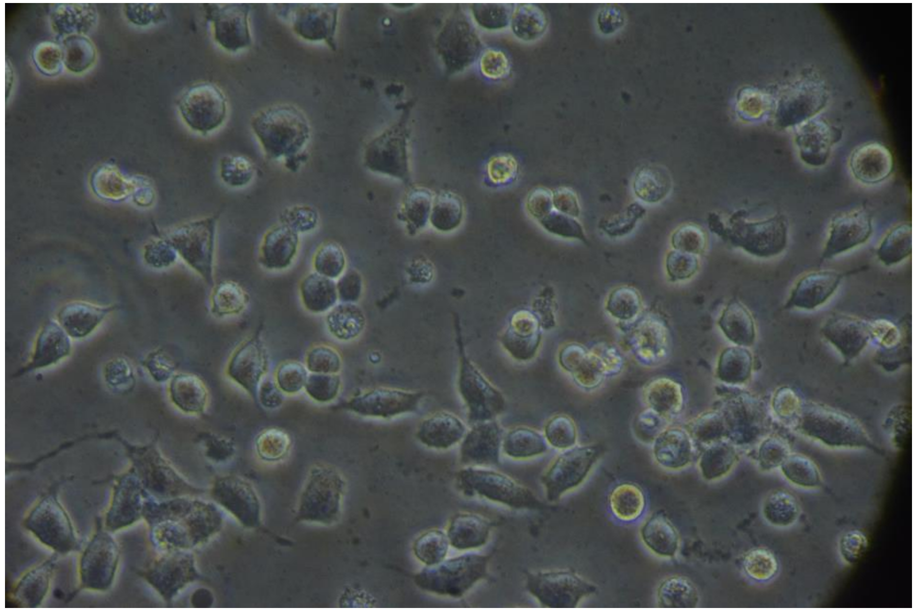

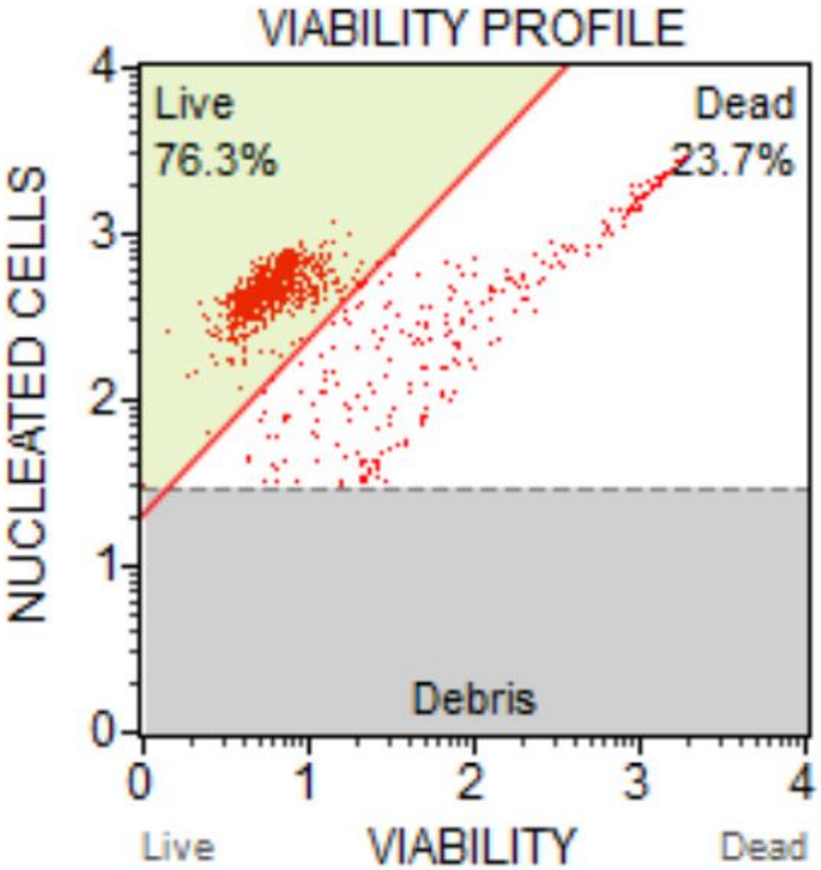

Figure 3A

750ng/ml rh-omentin + H/R

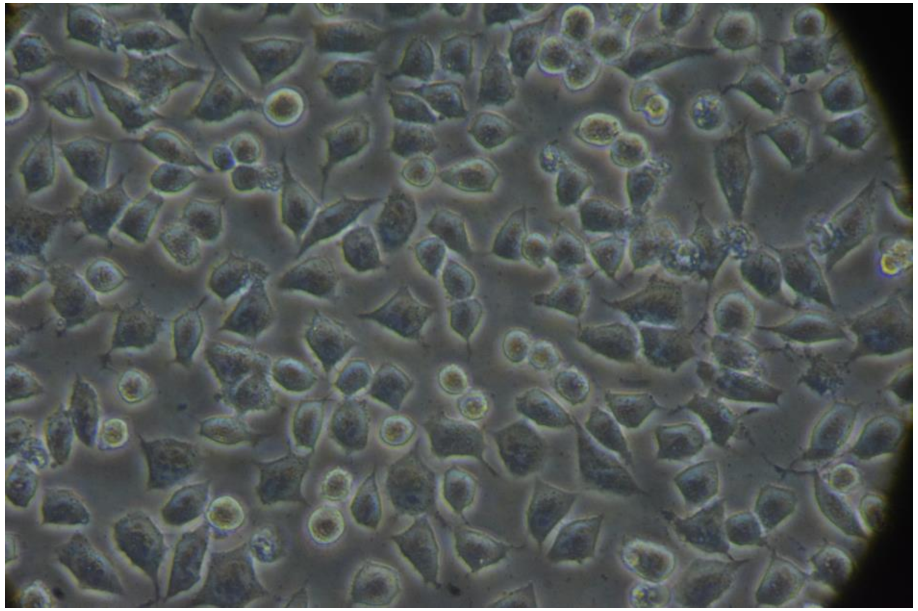

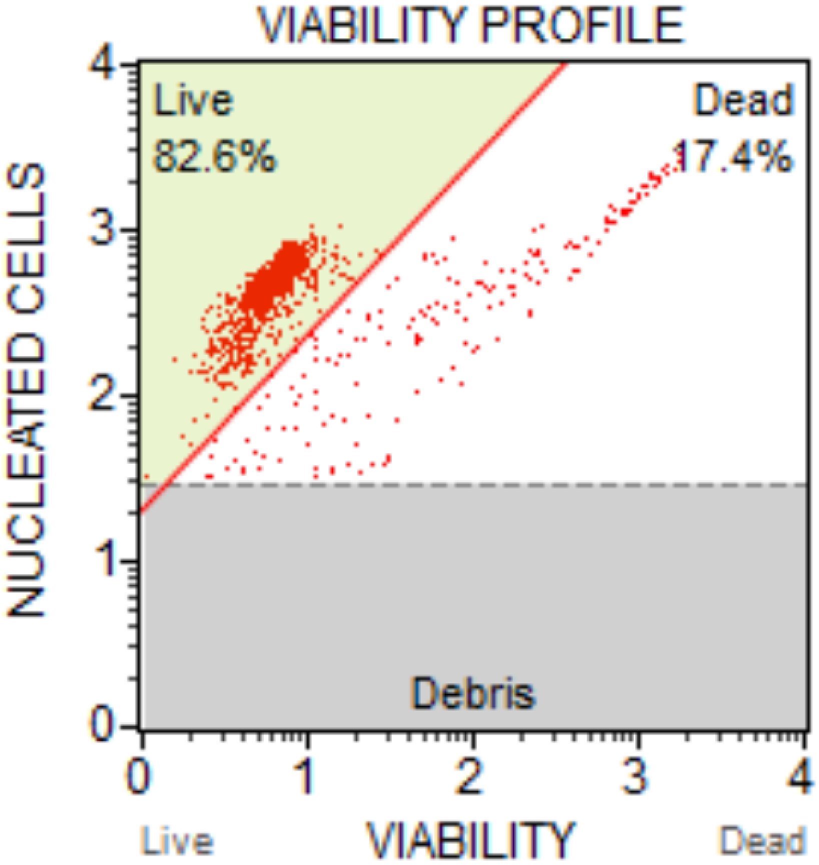

Figure 3A

1000ng/ml rh-omentin
+ H/R

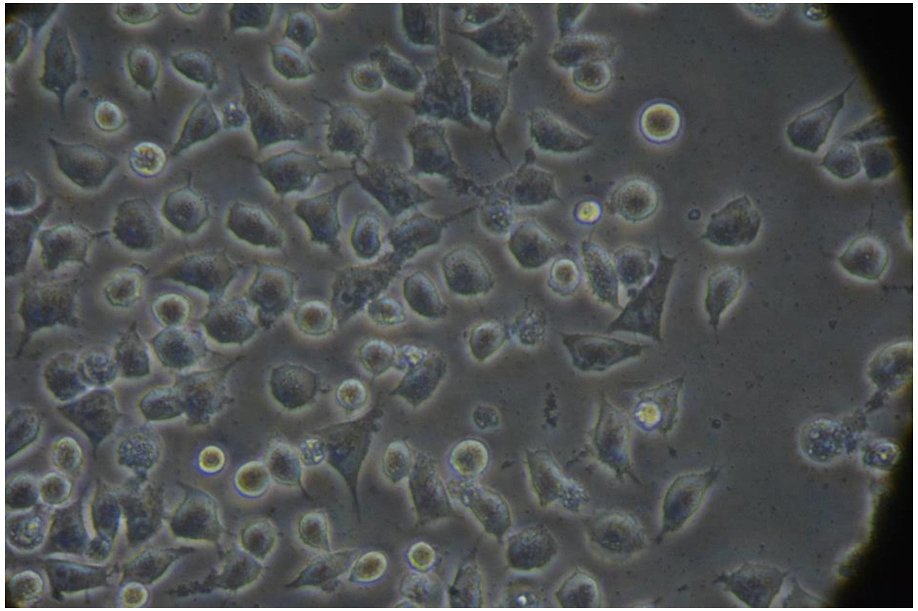

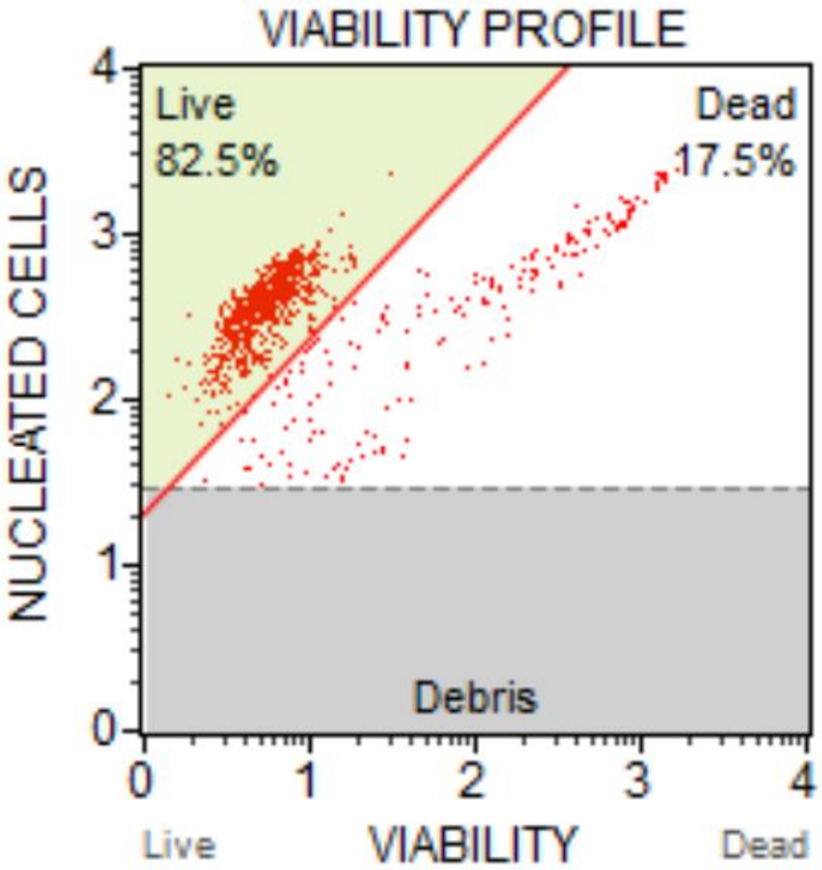

Figure 3A

1250ng/ml rh-omentin
+ H/R

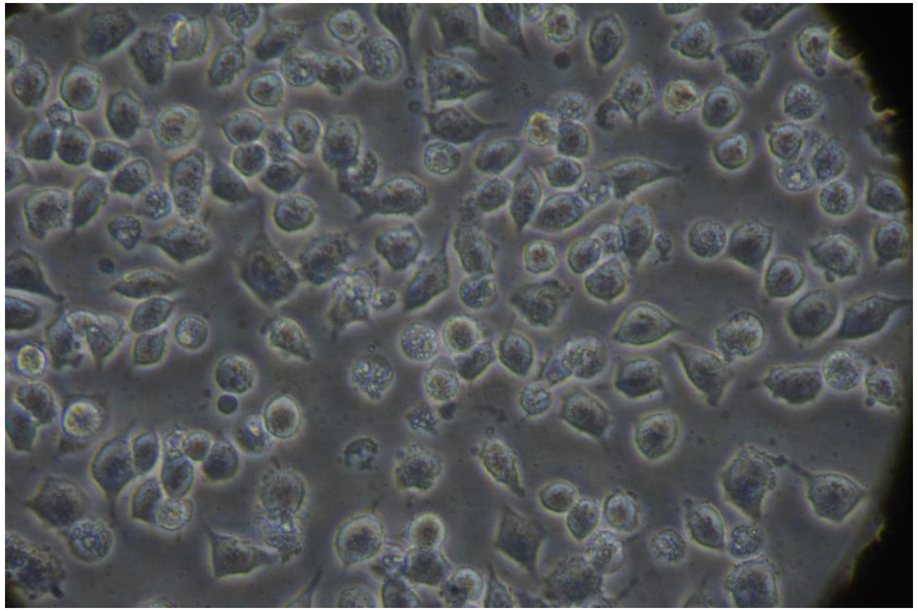

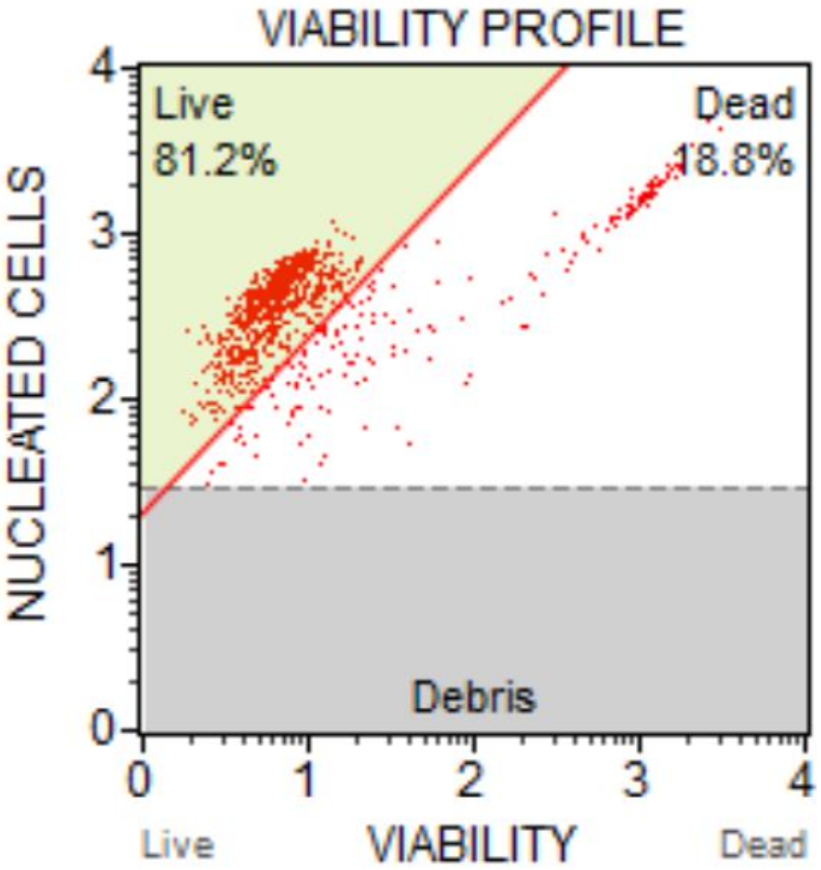

Figure 4C

Control

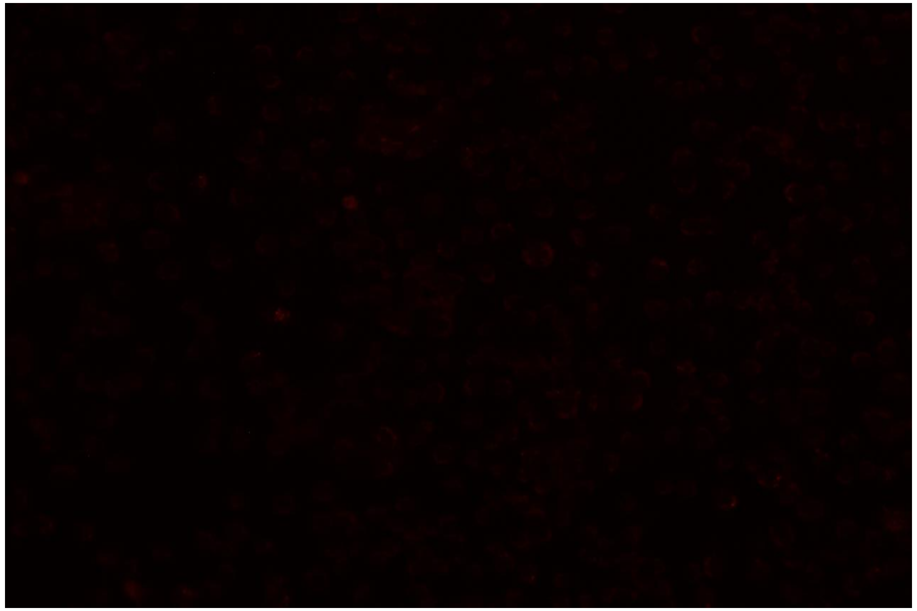

Figure 4C

H/R

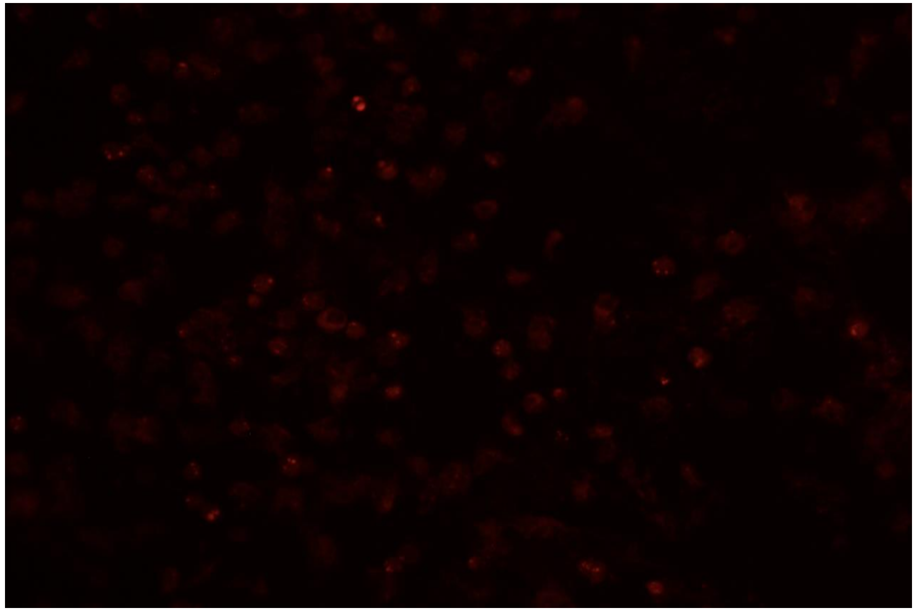

Figure 4C

750ng/ml rh-omentin + H/R

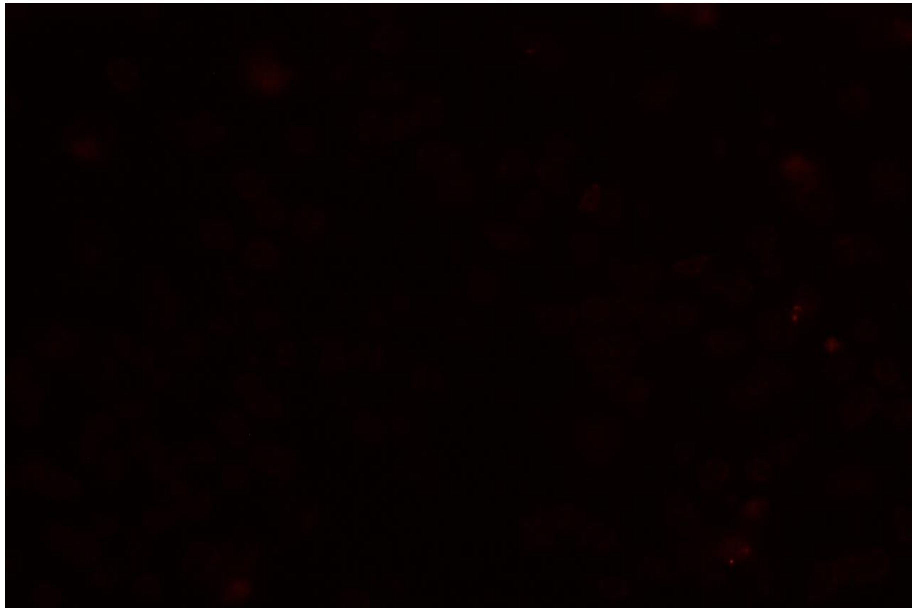

Figure 6A

NC siRNA H/R

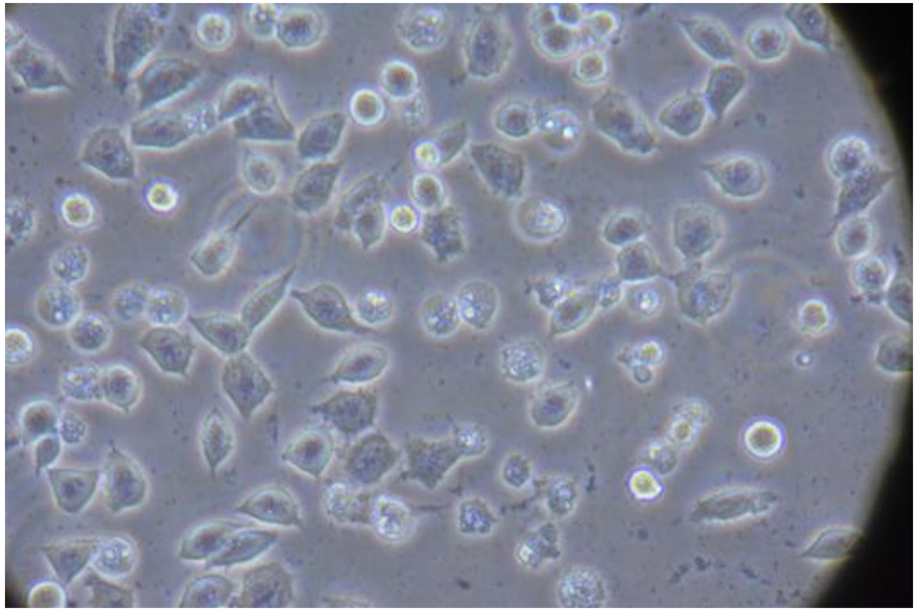

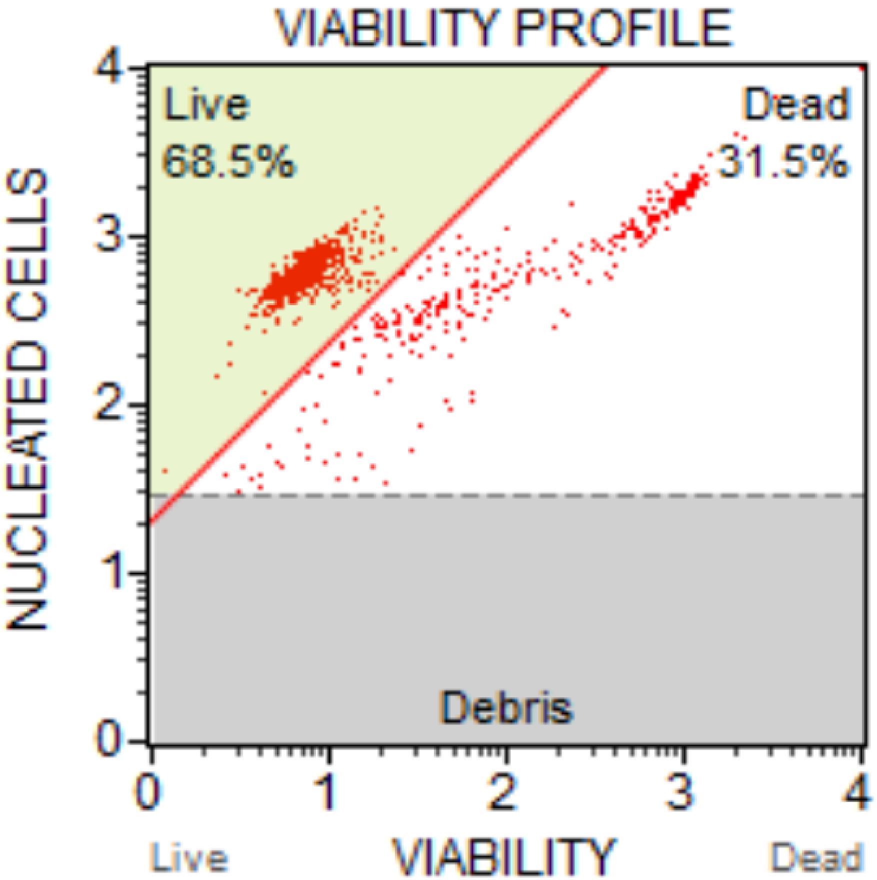

Figure 6A

NC siRNA H/R
+ rh-omentin

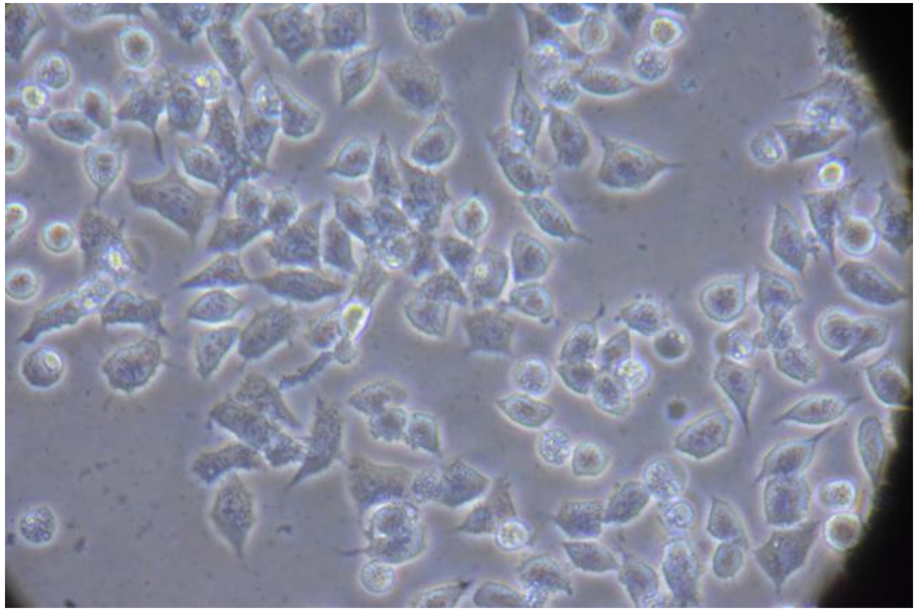

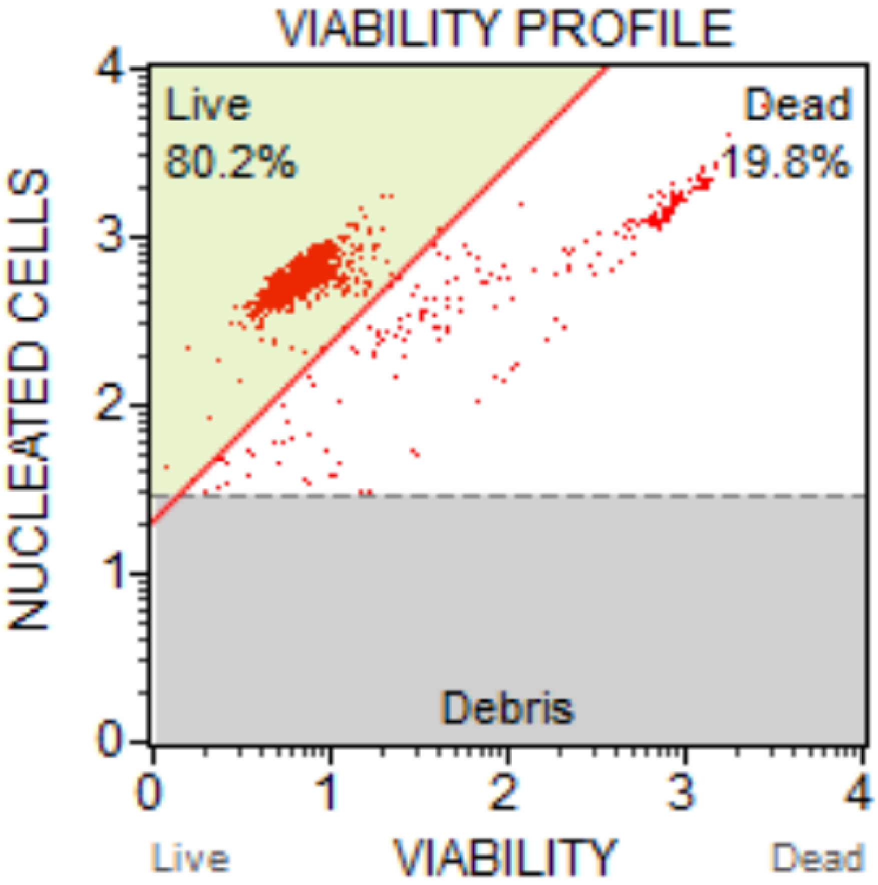

Figure 6A

GAS6 siRNA H/R
+ rh-omentin

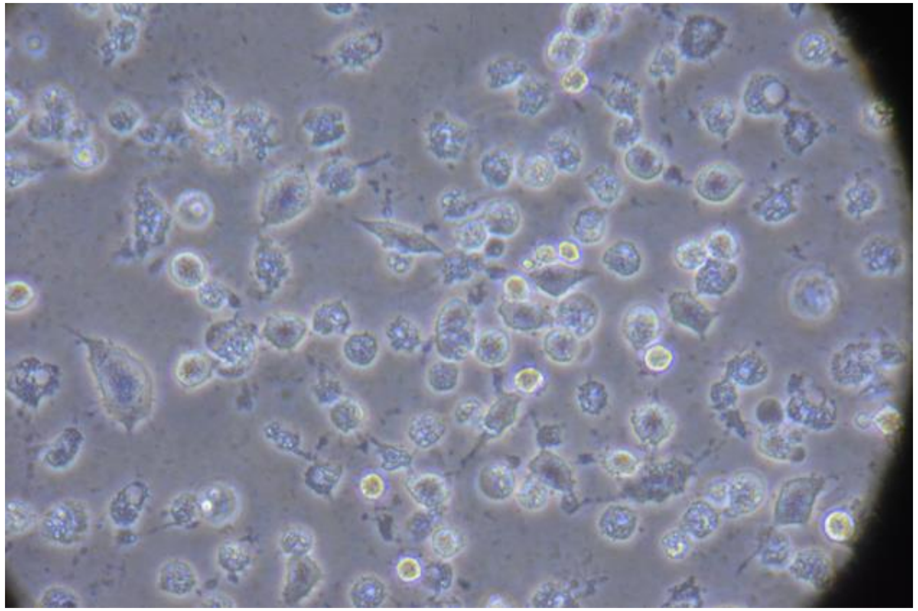

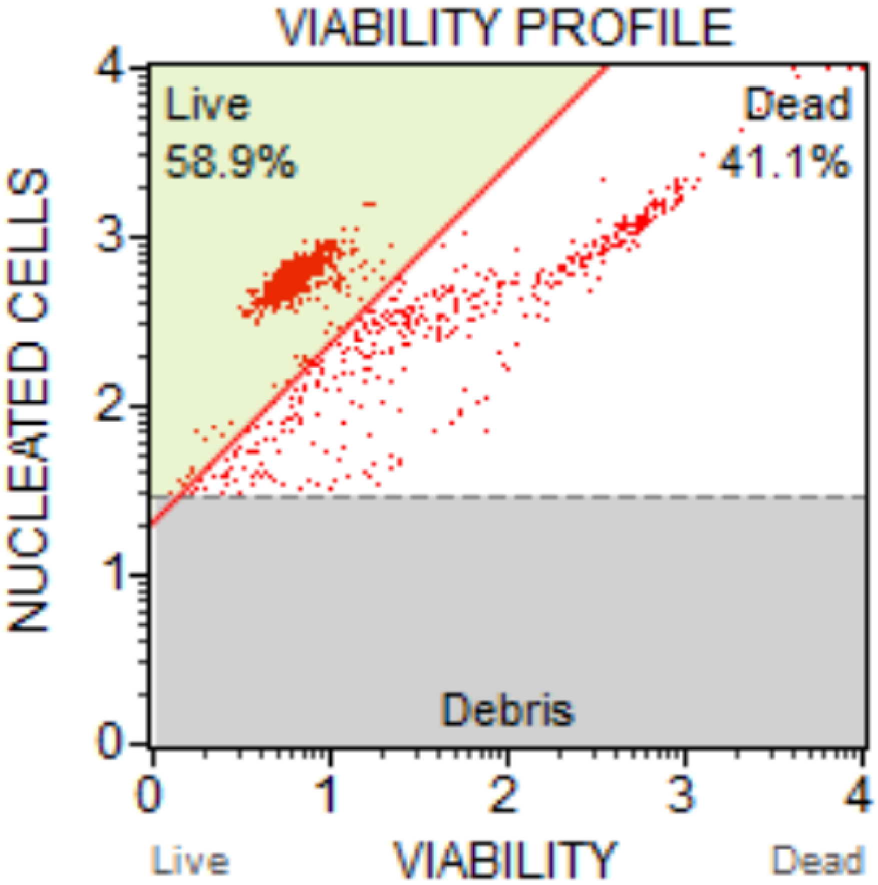

Figure 6A

GAS6 siRNA H/R

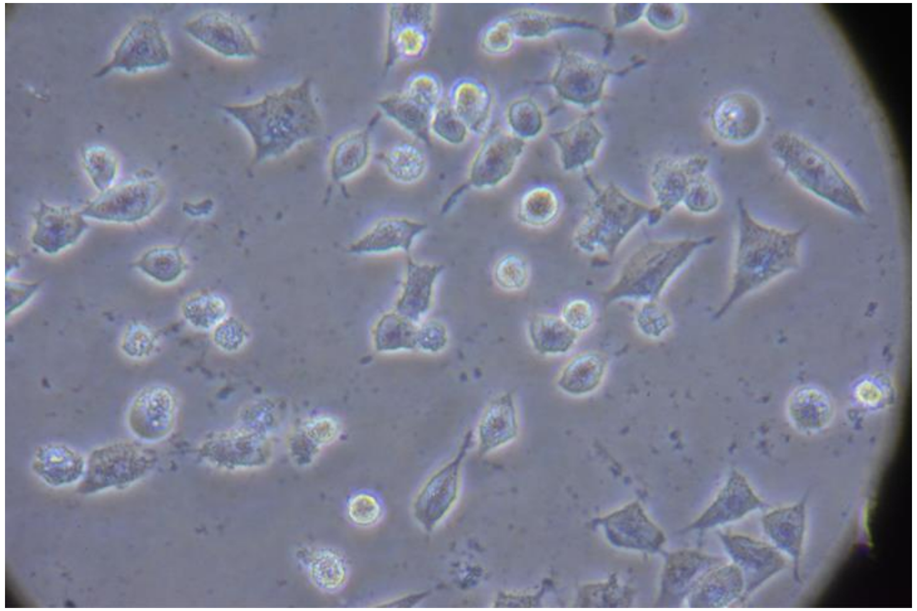

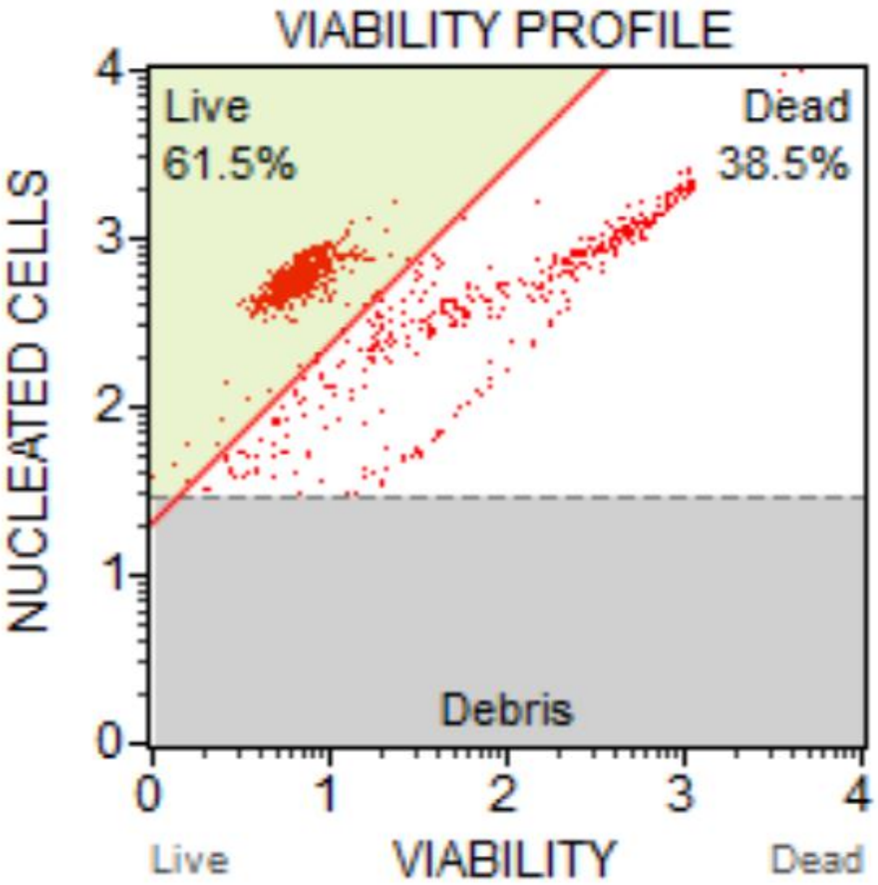

Figure 6C

NC siRNA H/R

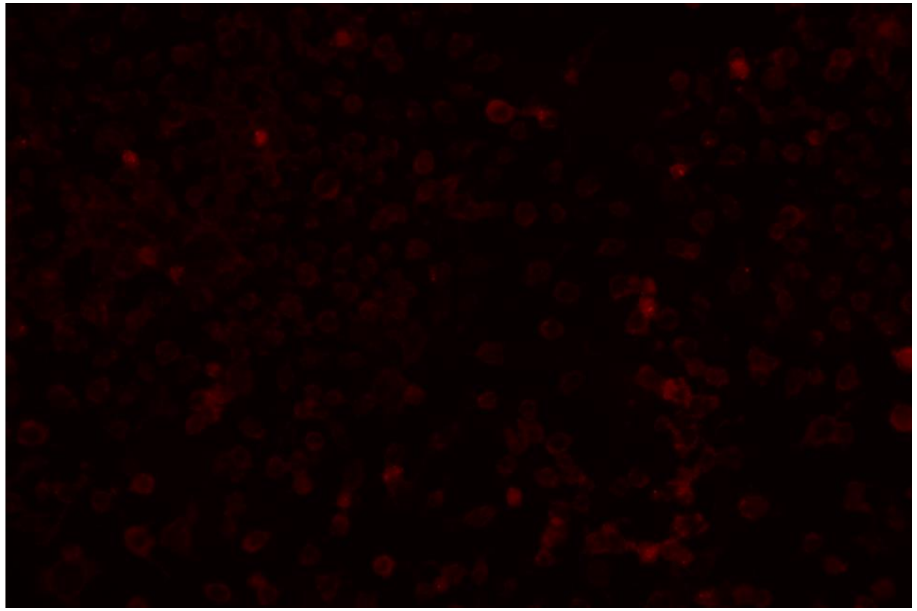

Figure 6C

NC siRNA H/R + rh-omentin

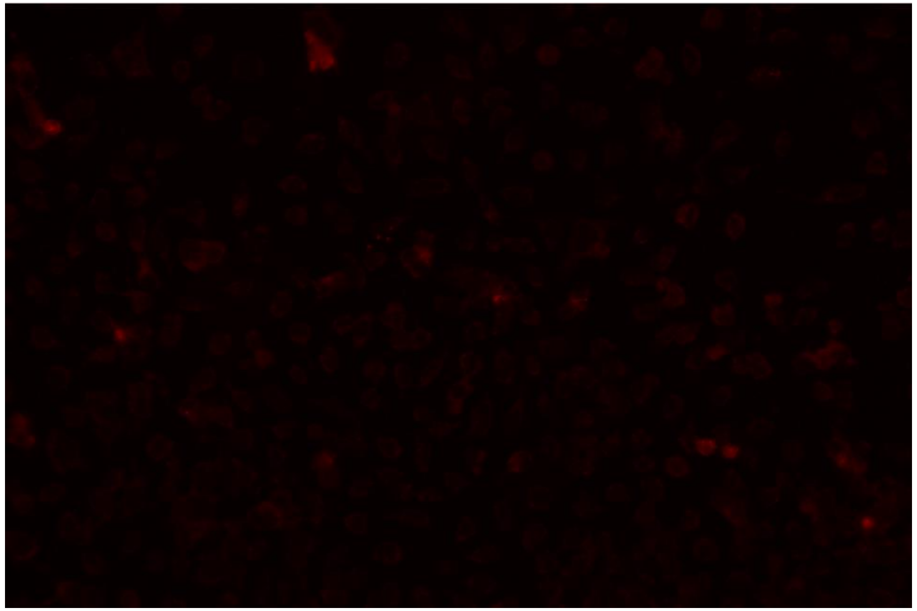

Figure 6C

GAS6 siRNA H/R + rh-omentin

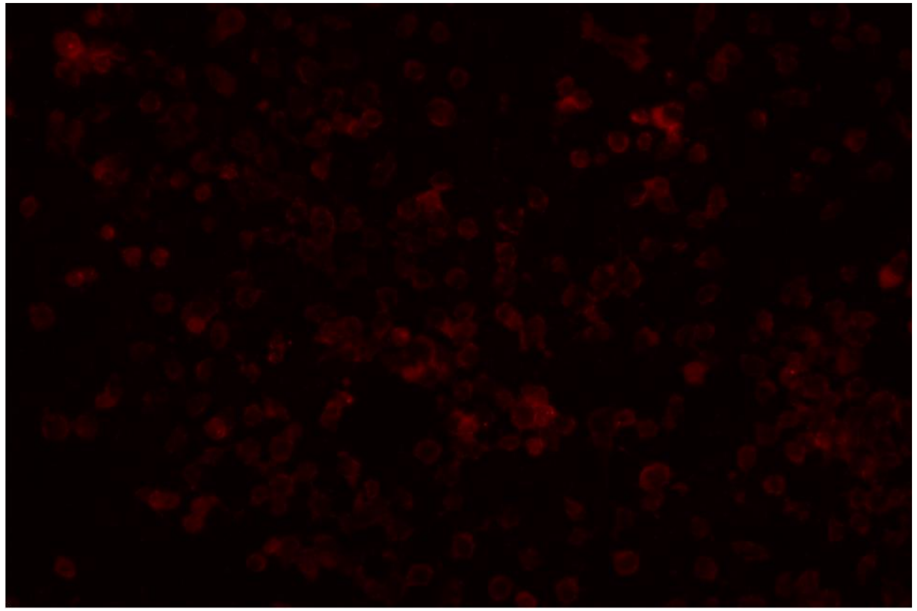

Figure 6C

GAS6 siRNA H/R

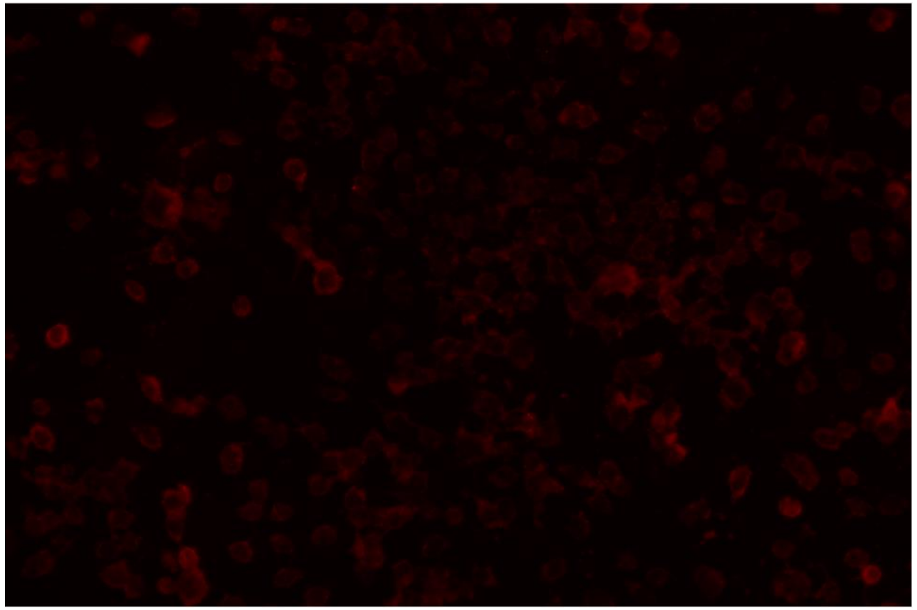

Figure 8D

Control N2a-Mock

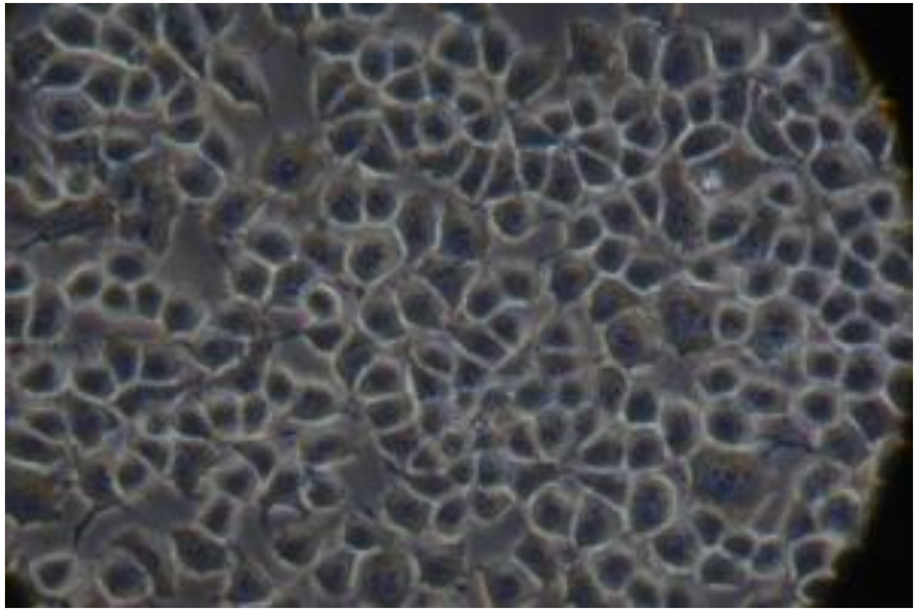

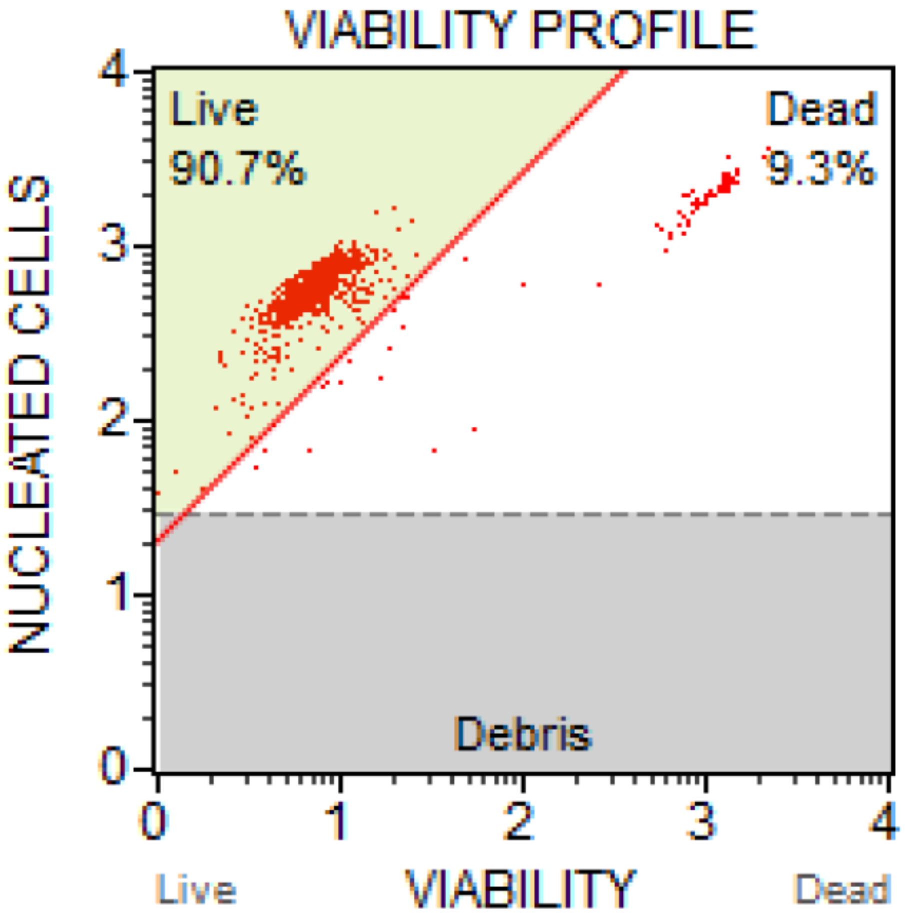

Figure 8D

Control N2a-ITLN

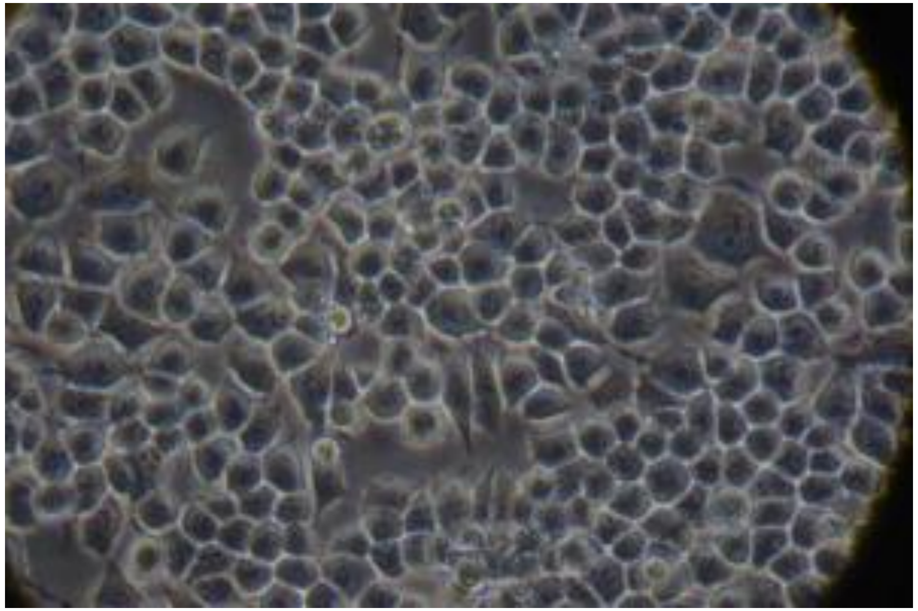

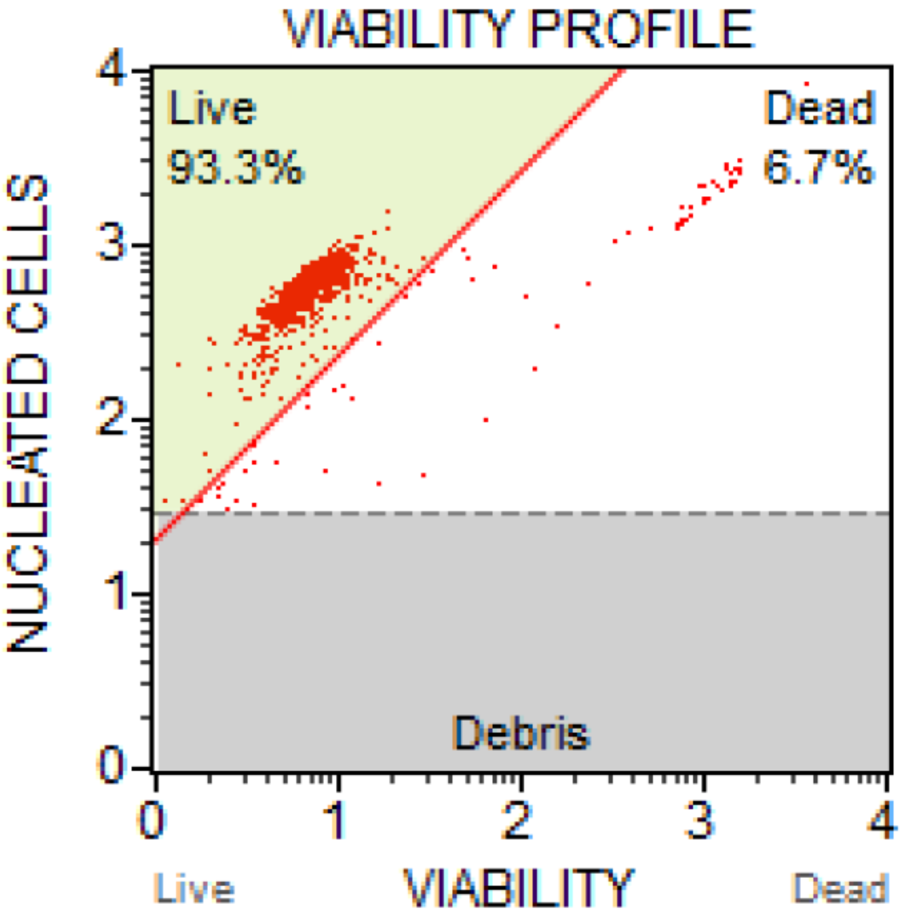

Figure 8D

H/R N2a-Mock

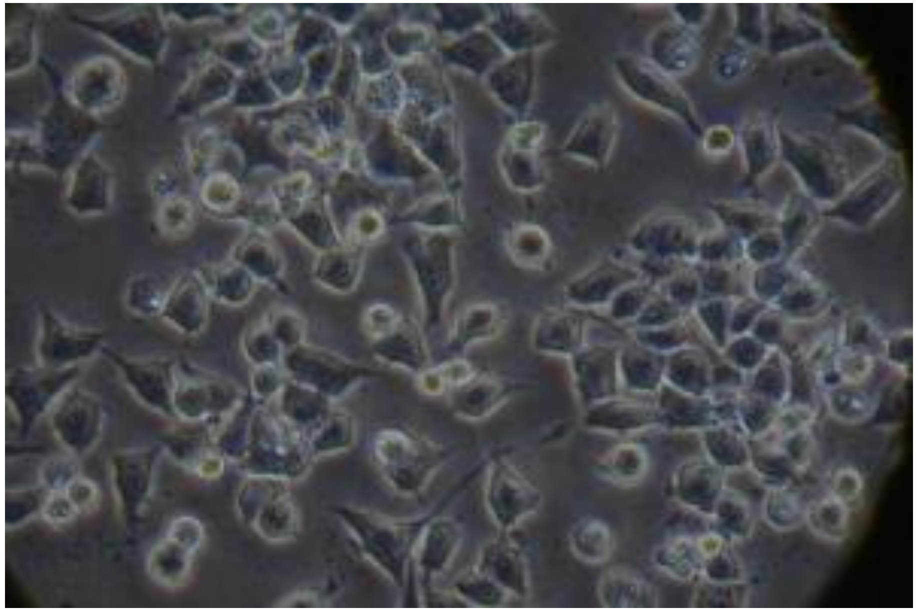

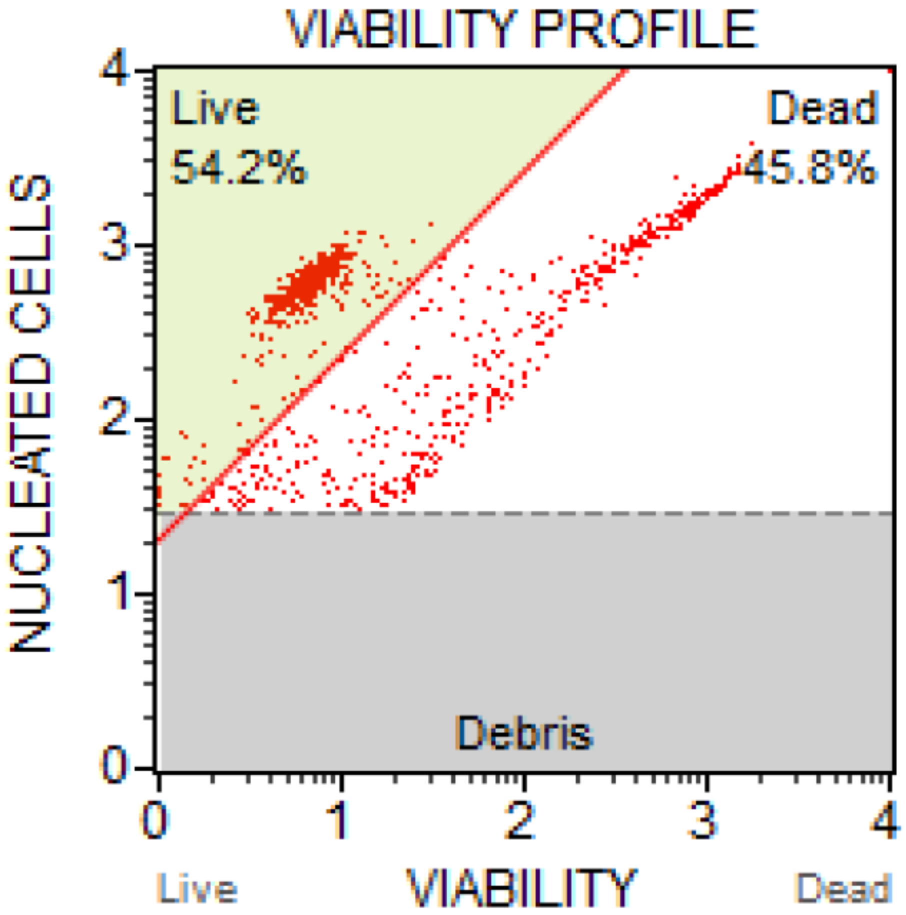

Figure 8D

H/R N2a-ITLN

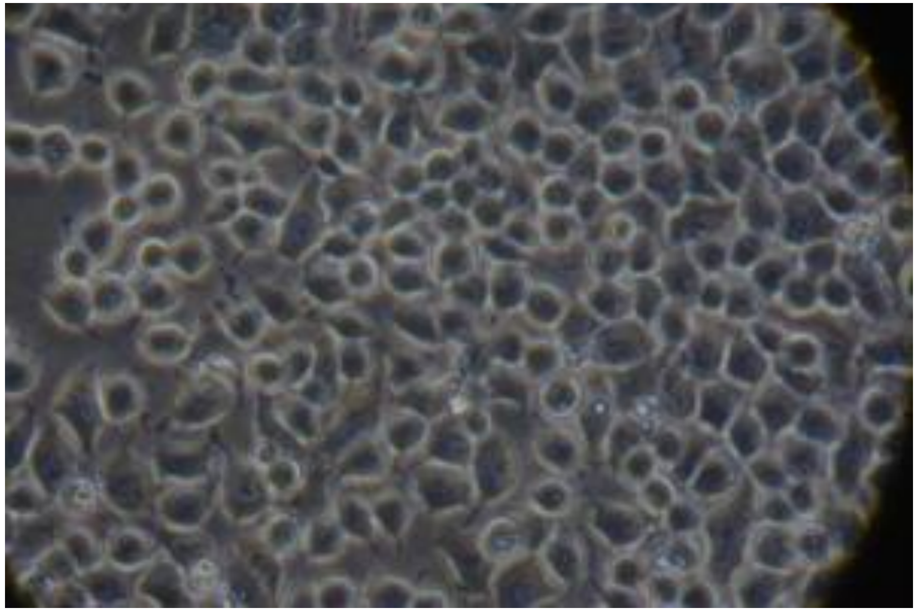

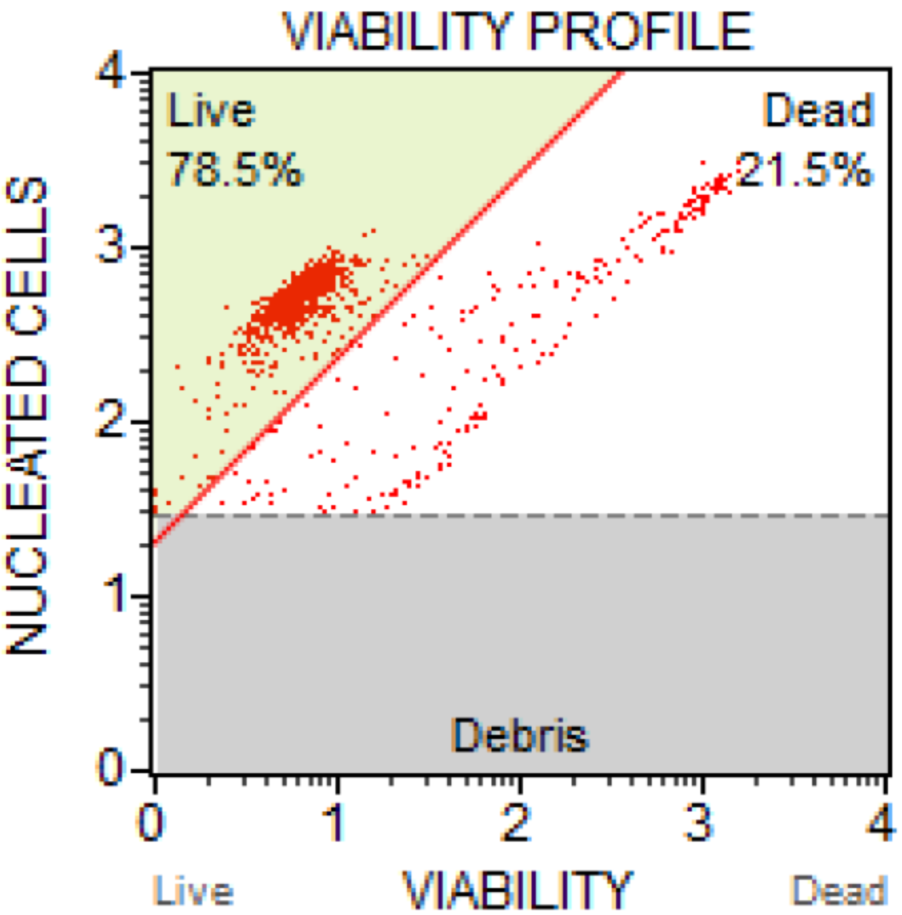

Figure 9A

Control N2a-Mock

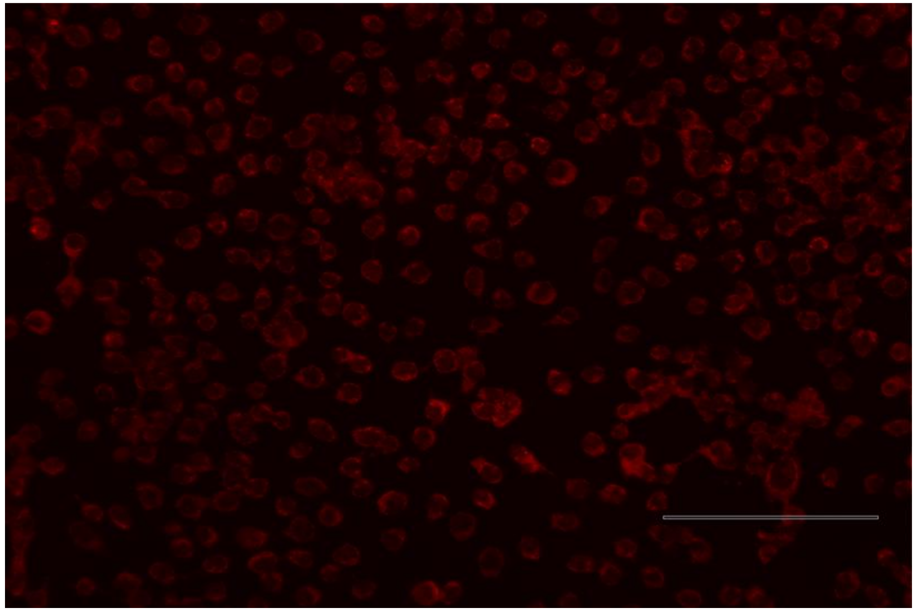

Figure 9A

Control N2a-ITLN

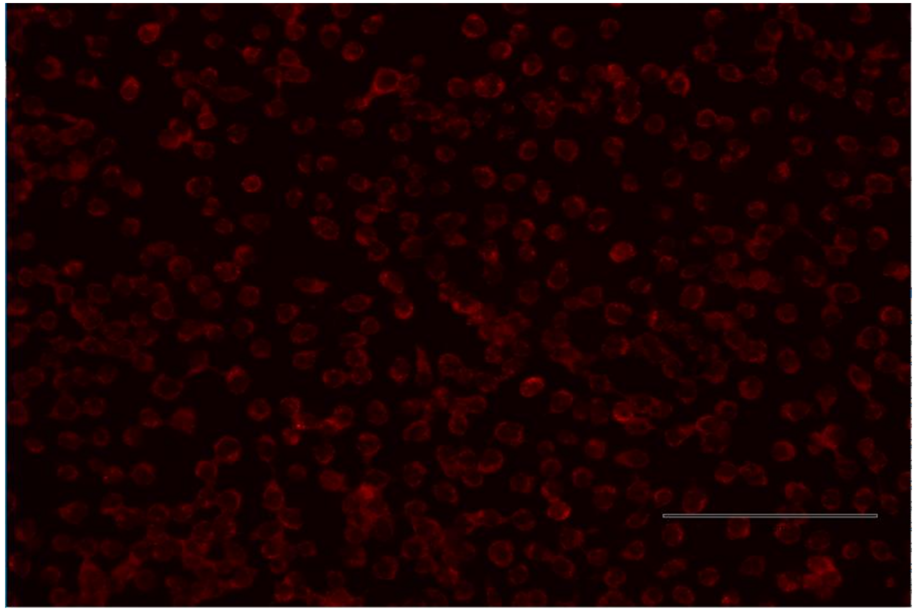

Figure 9A

H/R N2a-Mock

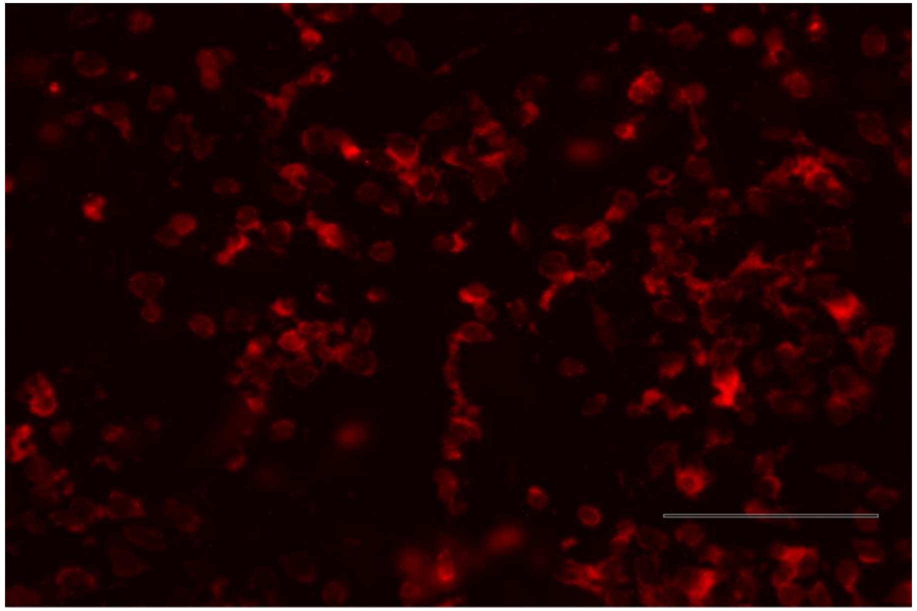

Figure 9A

H/R N2a-ITLN

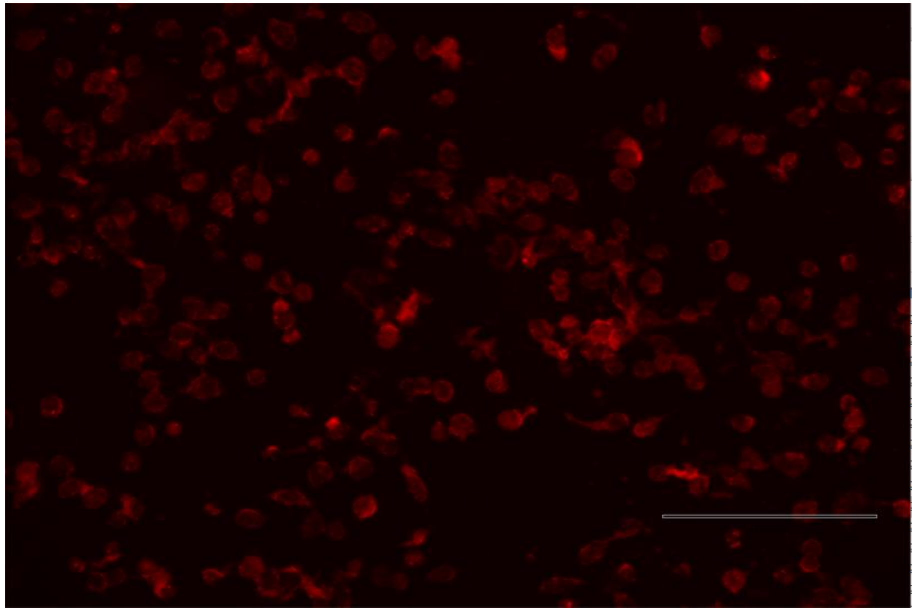

Supplementary Figure 1A

Control

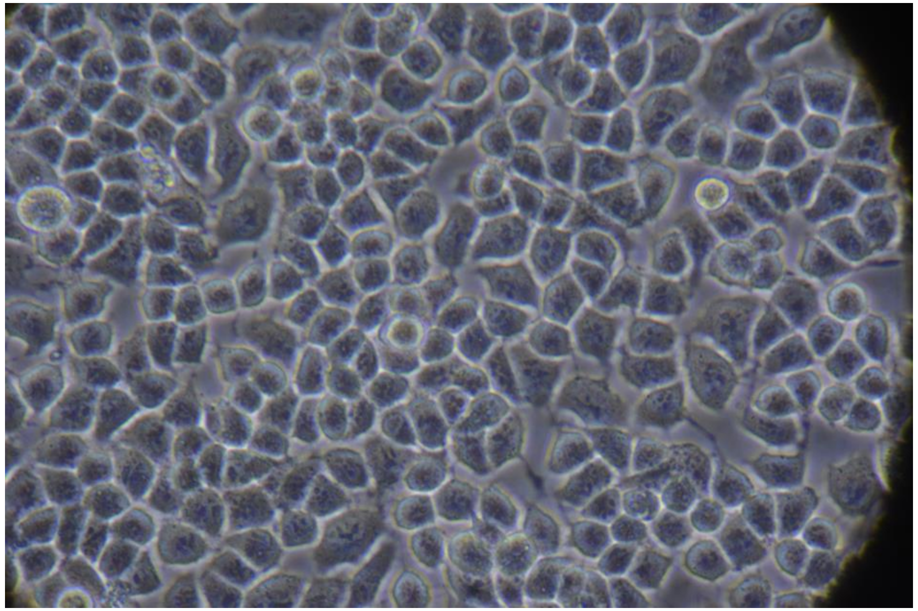

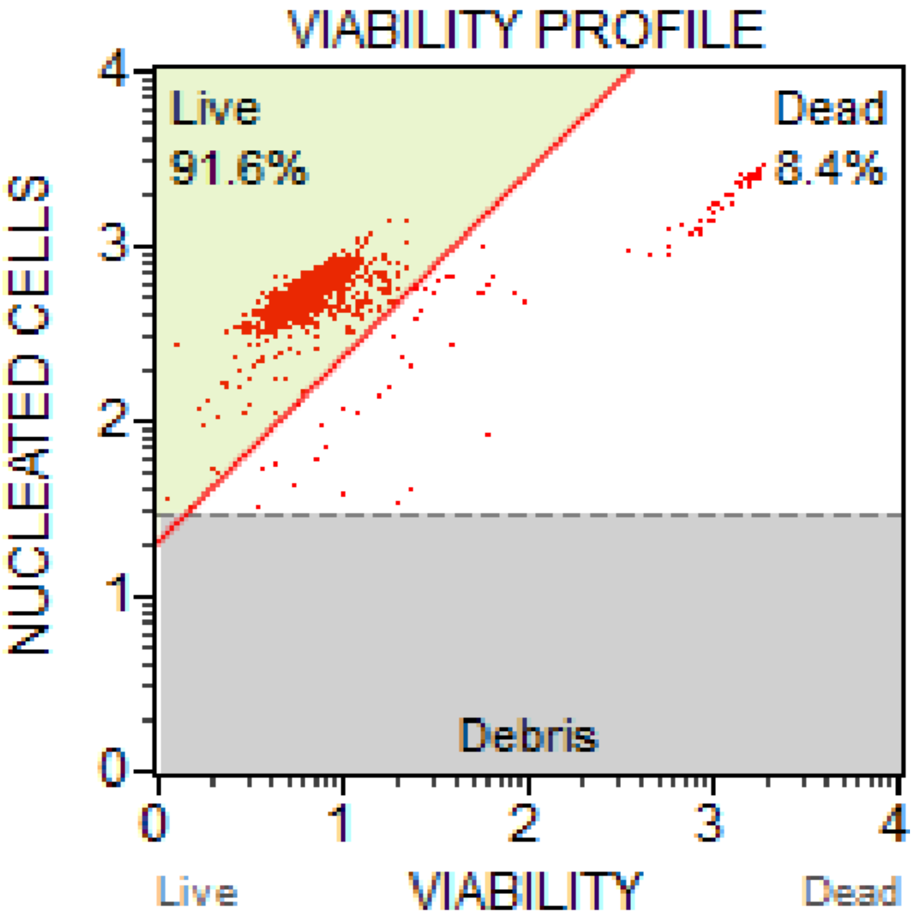

Supplementary Figure 1A

H 12h/R 3h

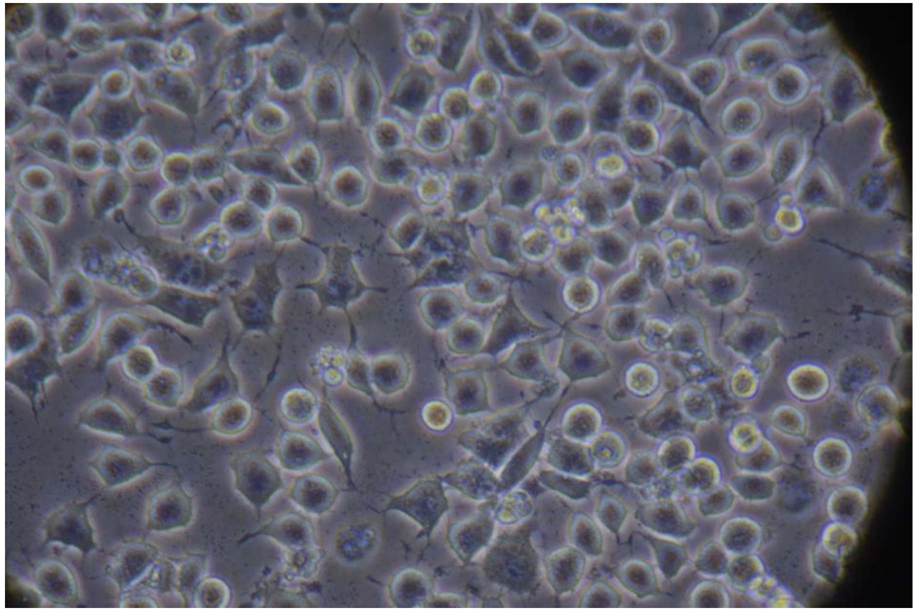

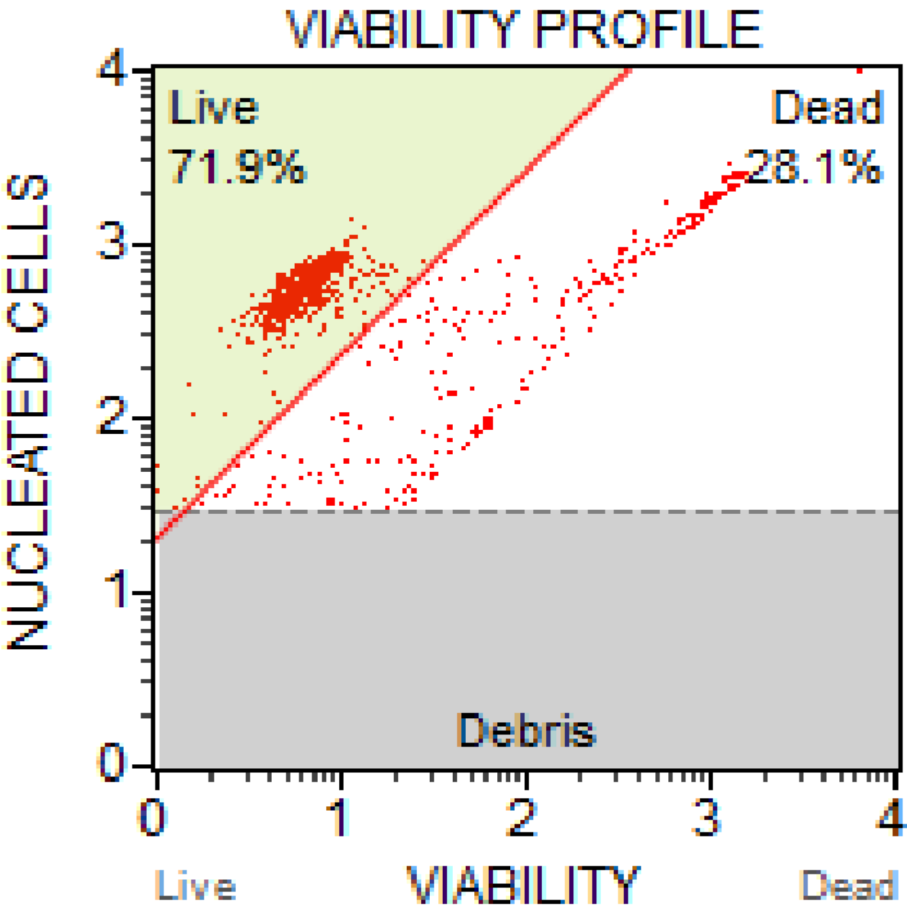

Supplementary Figure 1A

H 12h/R 6h

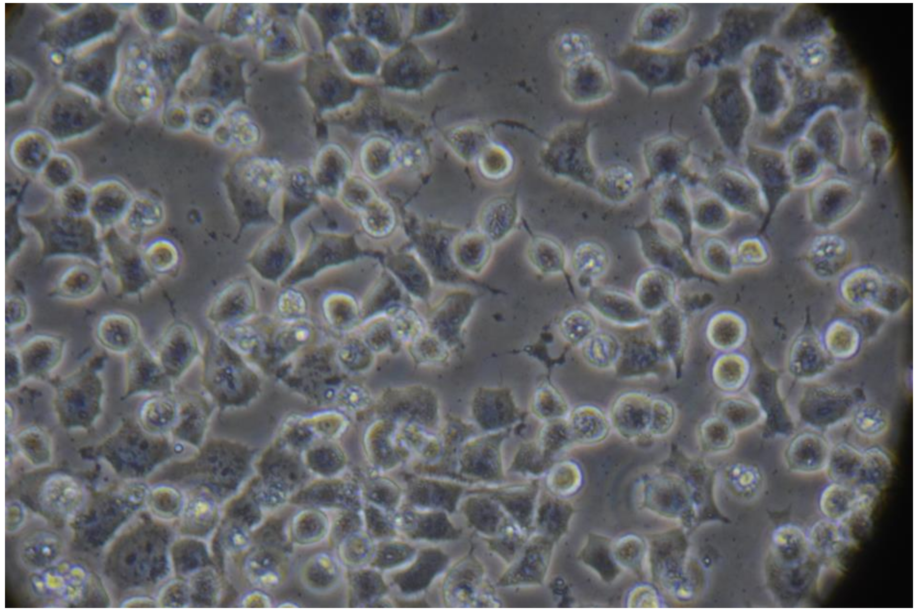

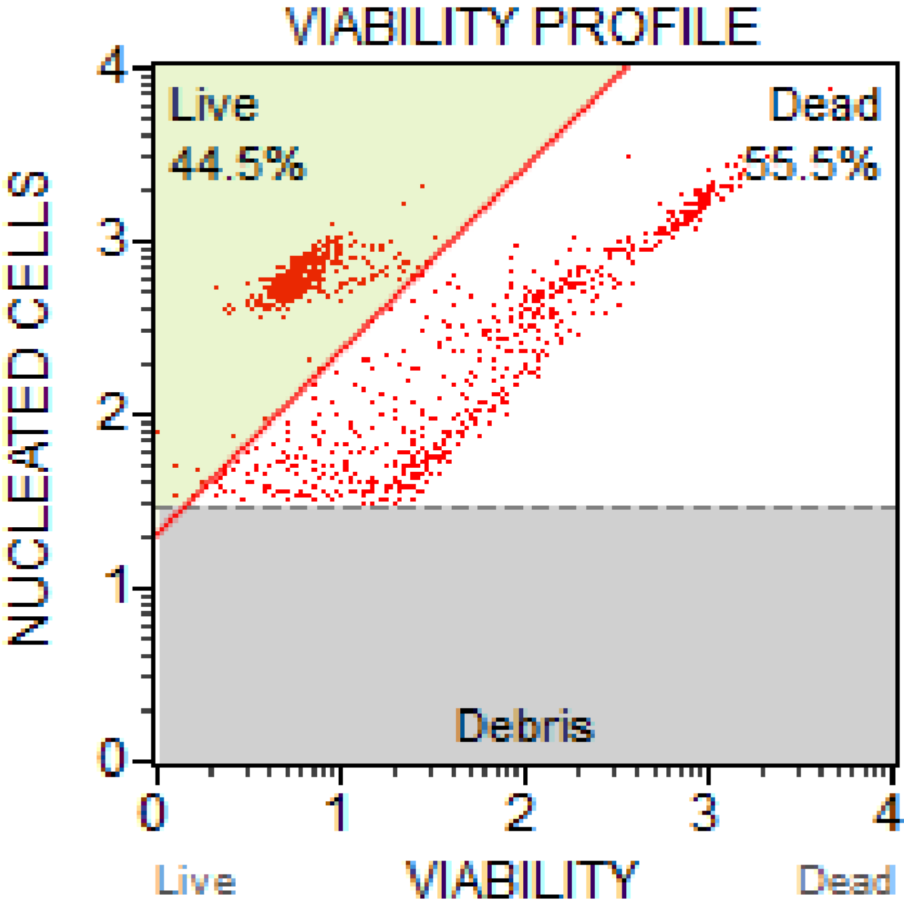

Supplementary Figure 1A

H 12h/R 9h

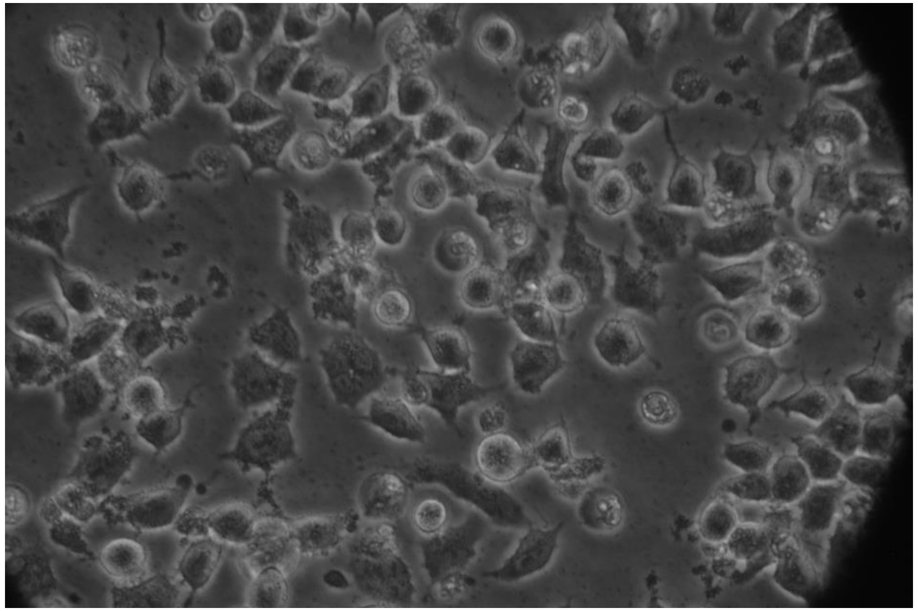

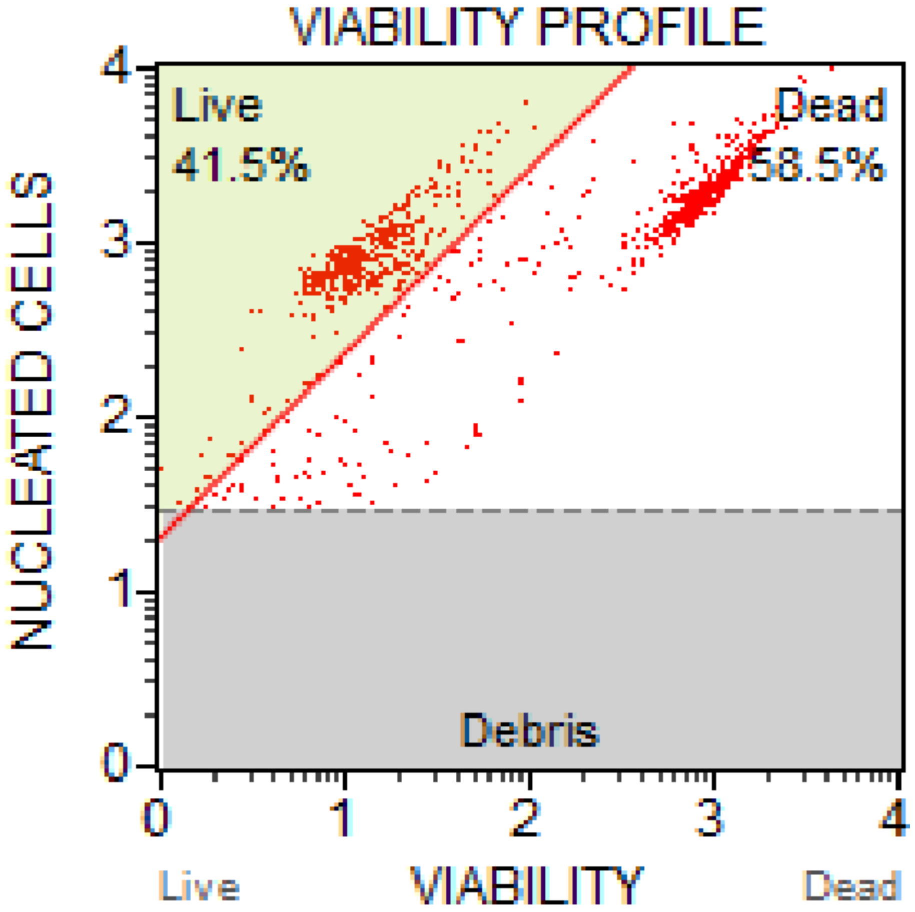

Supplementary Figure 3A

DMEM-FBS GAP43

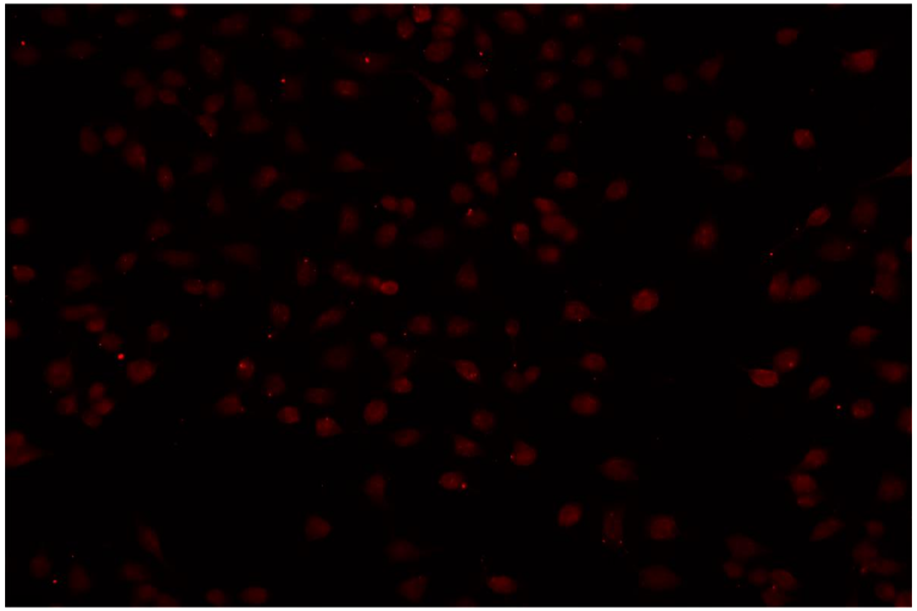

Supplementary Figure 3A

DMEM GAP43

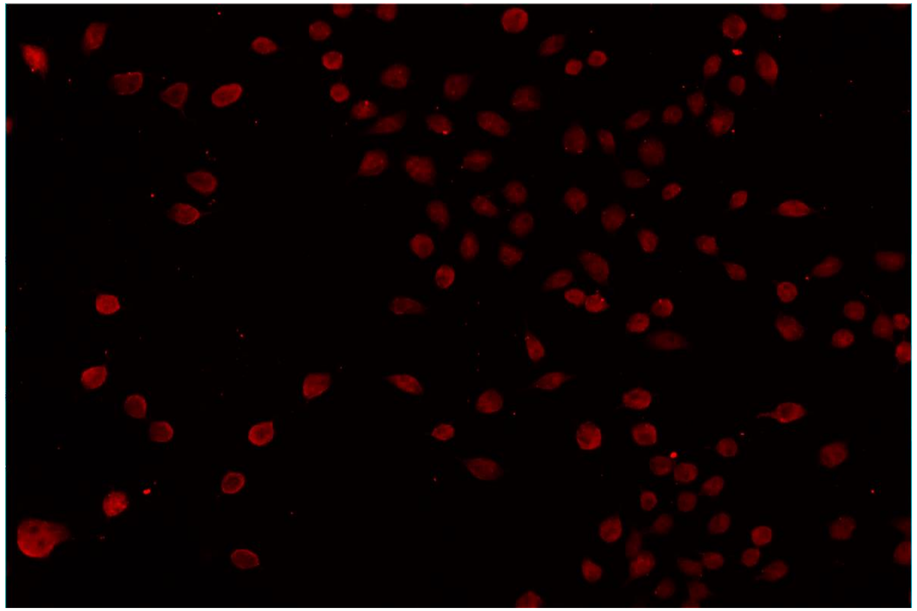

Supplementary Figure 3A

DMEM+H/R GAP43

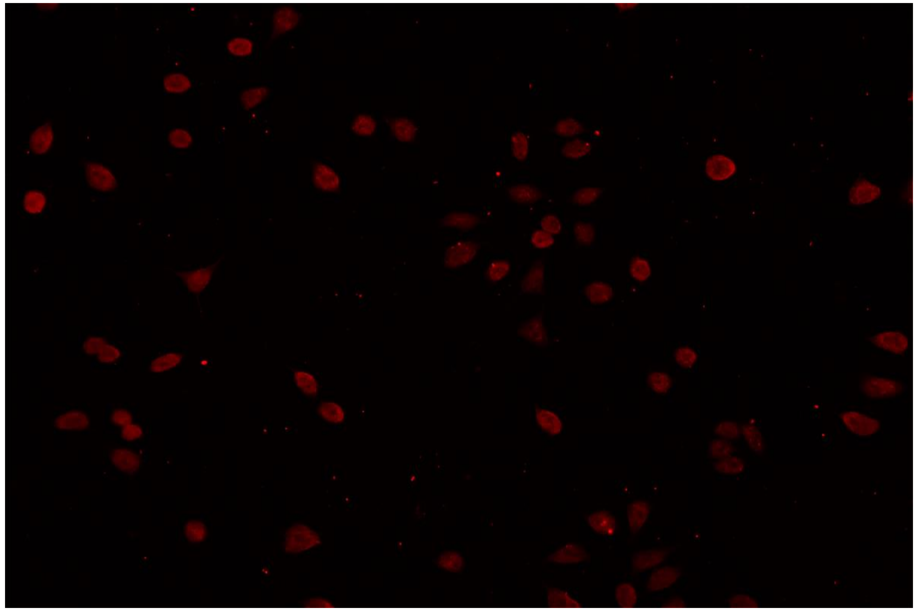

Supplementary Figure 3A

DMEM-FBS DAPI

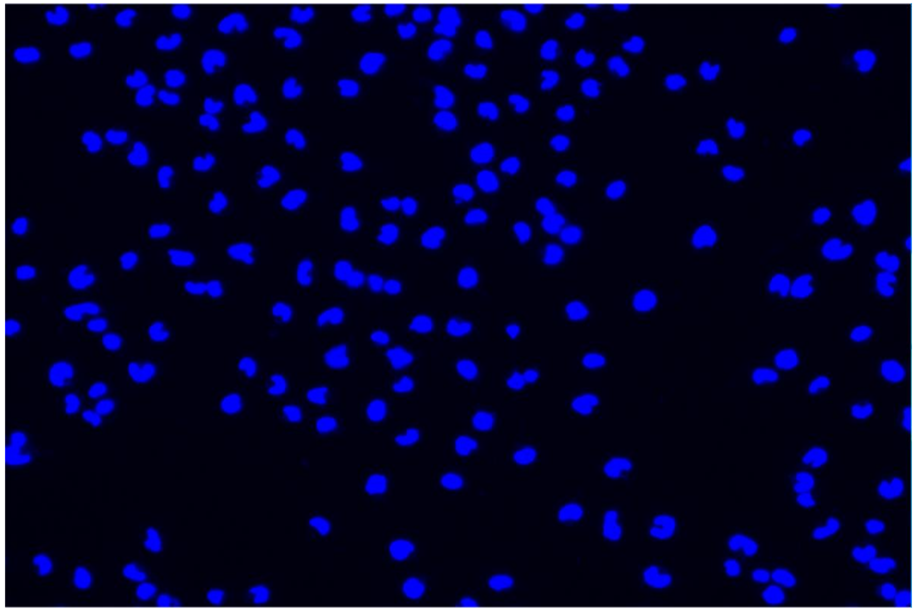

Supplementary Figure 3A

DMEM DAPI

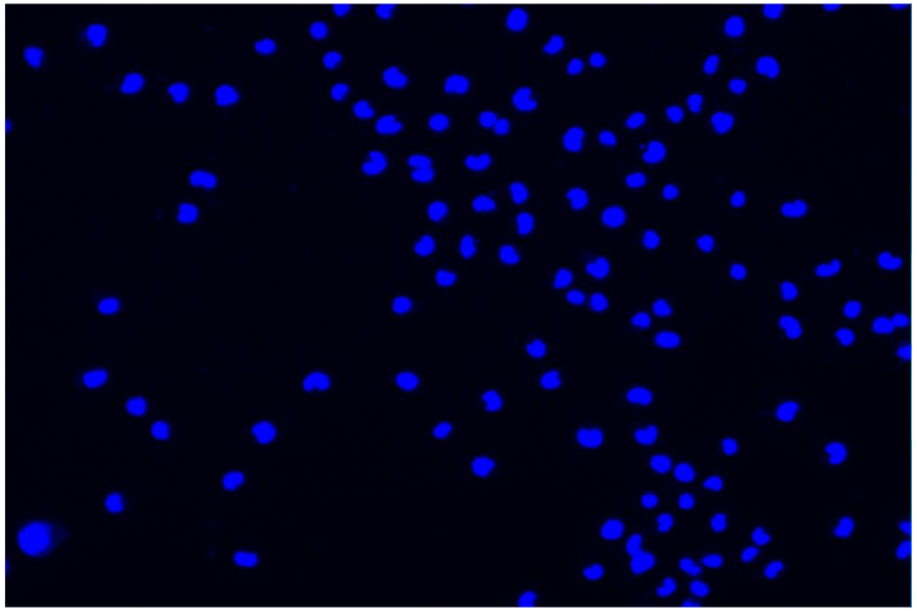

Supplementary Figure 3A

DMEM+H/R DAPI

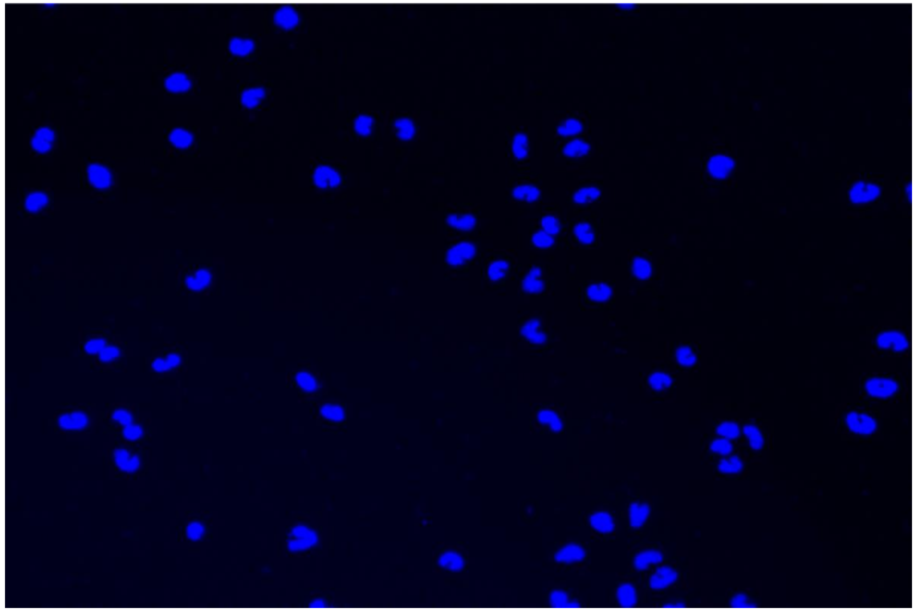

Supplementary Figure 3A

DMEM-FBS Merge

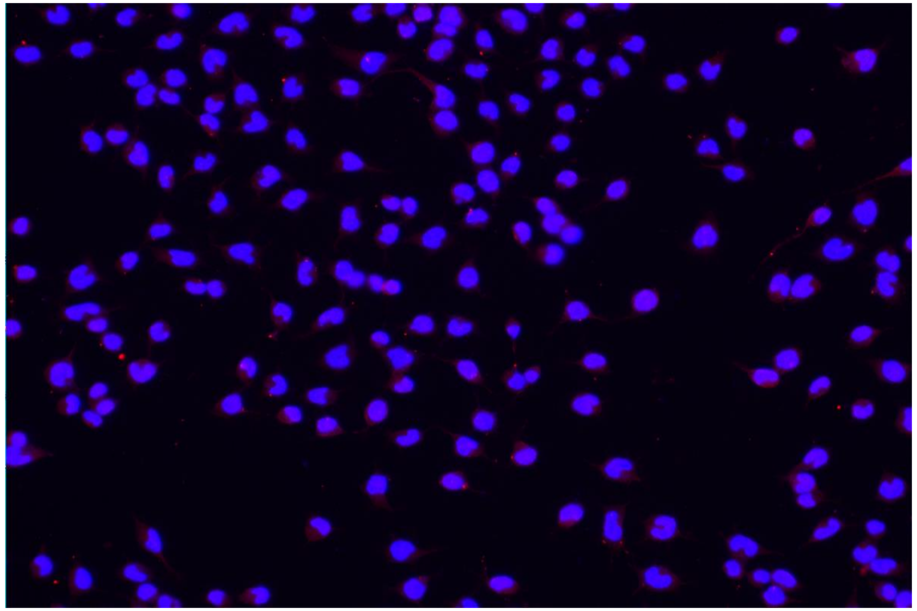

Supplementary Figure 3A

DMEM Merge

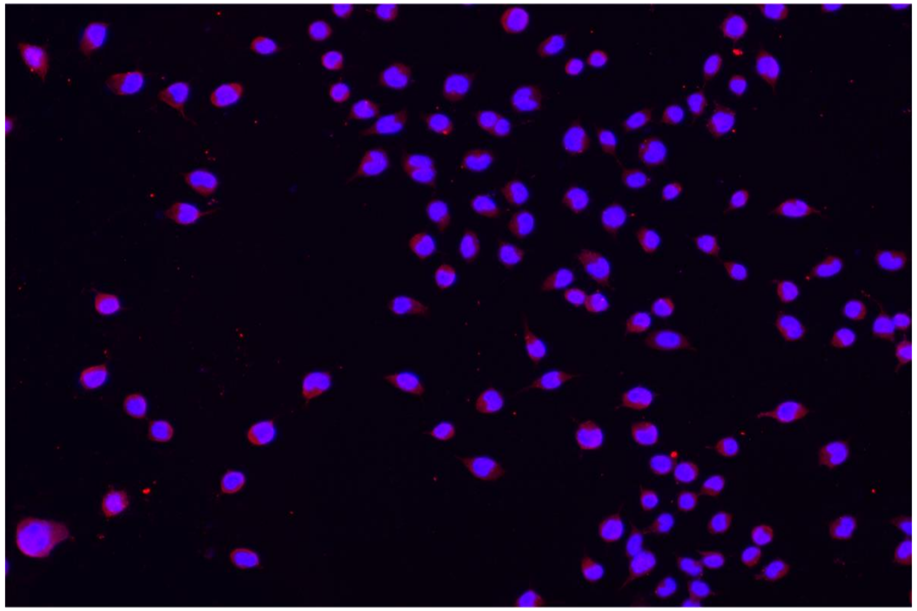

Supplementary Figure 3A

DMEM+H/R Merge

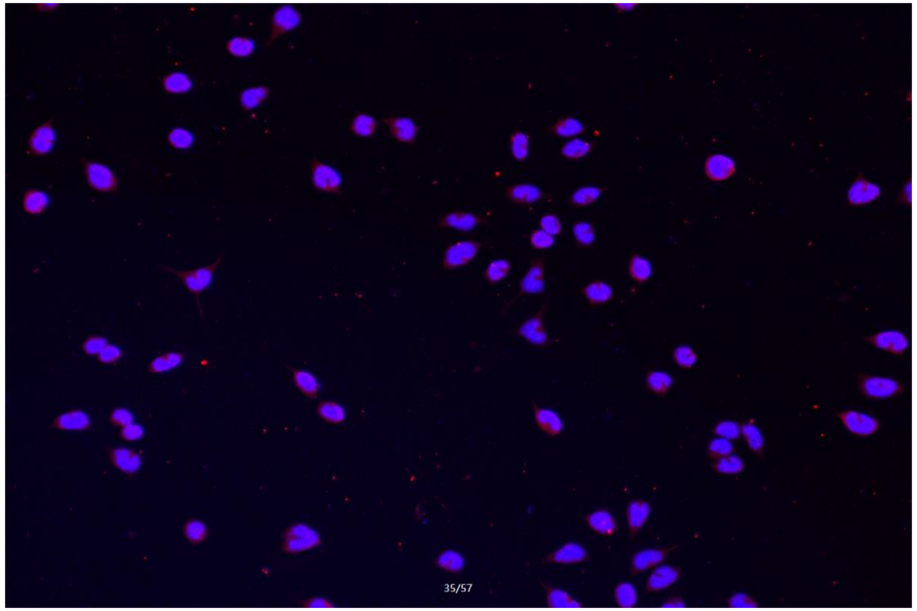

Supplementary Figure 3B

DMEM-FBS TUBB3

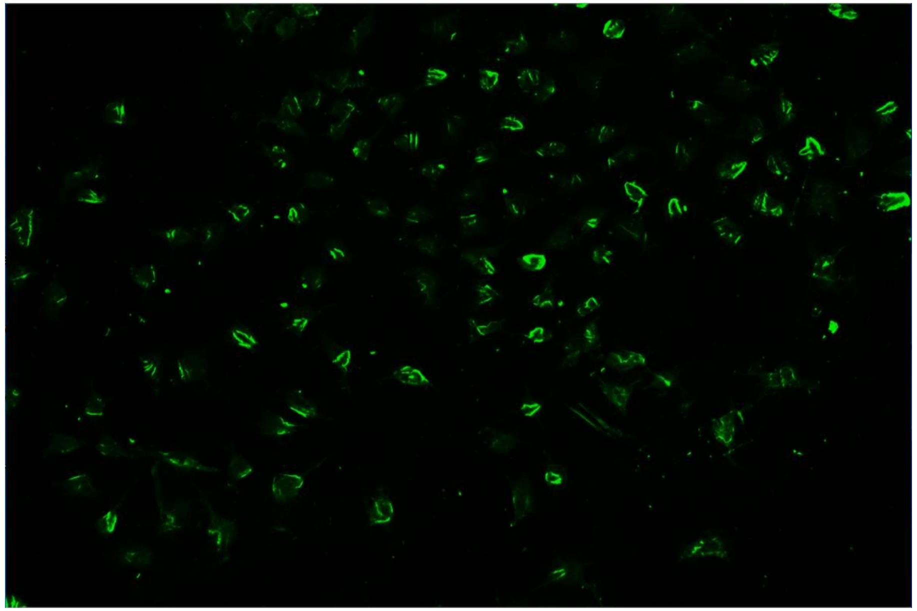

Supplementary Figure 3B

DMEM TUBB3

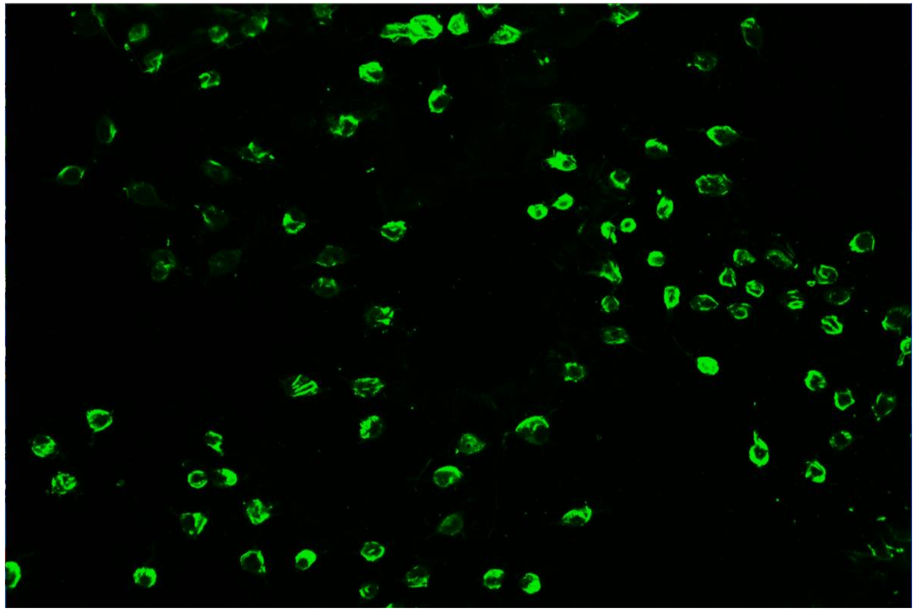

Supplementary Figure 3B

DMEM+H/R TUBB3

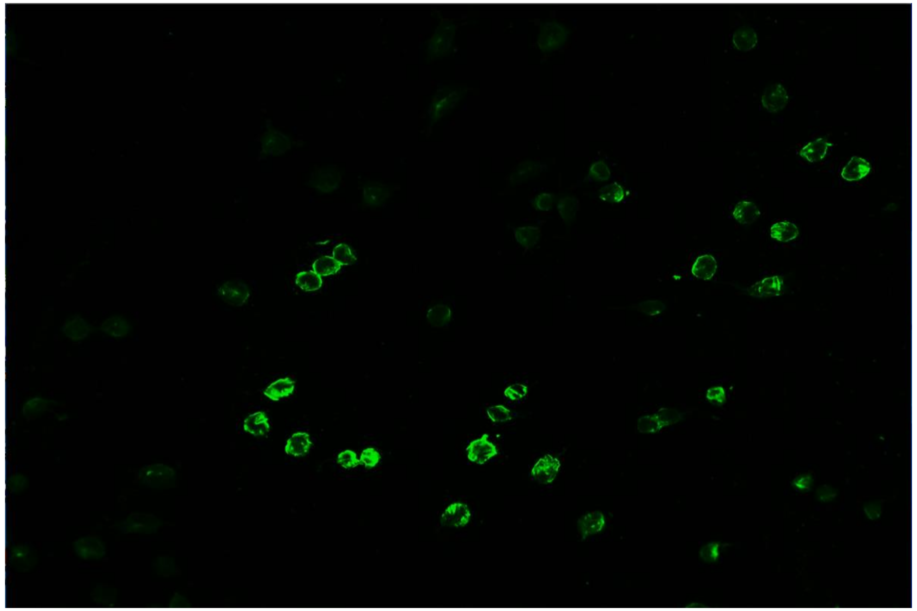

Supplementary Figure 3B

DMEM-FBS DAPI

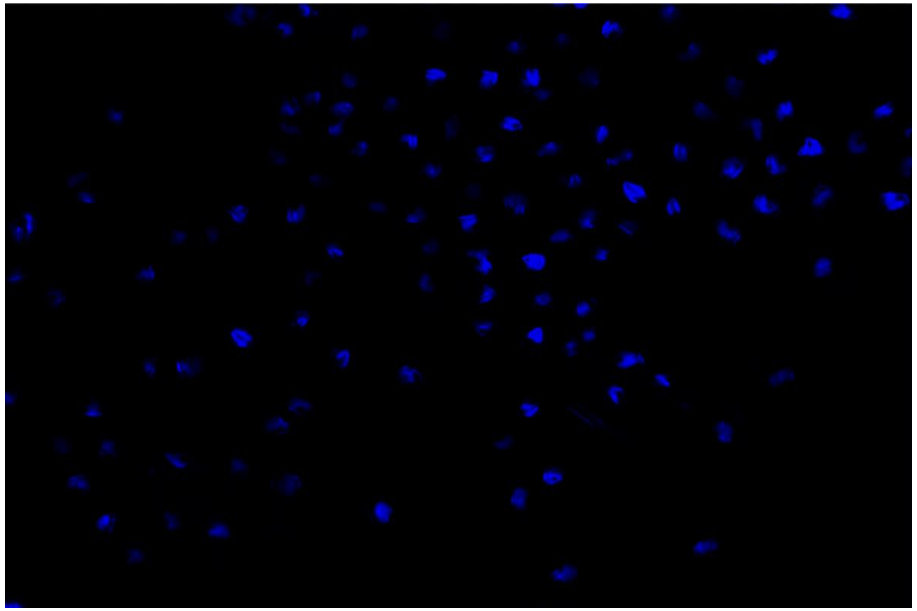

Supplementary Figure 3B

DMEM DAPI

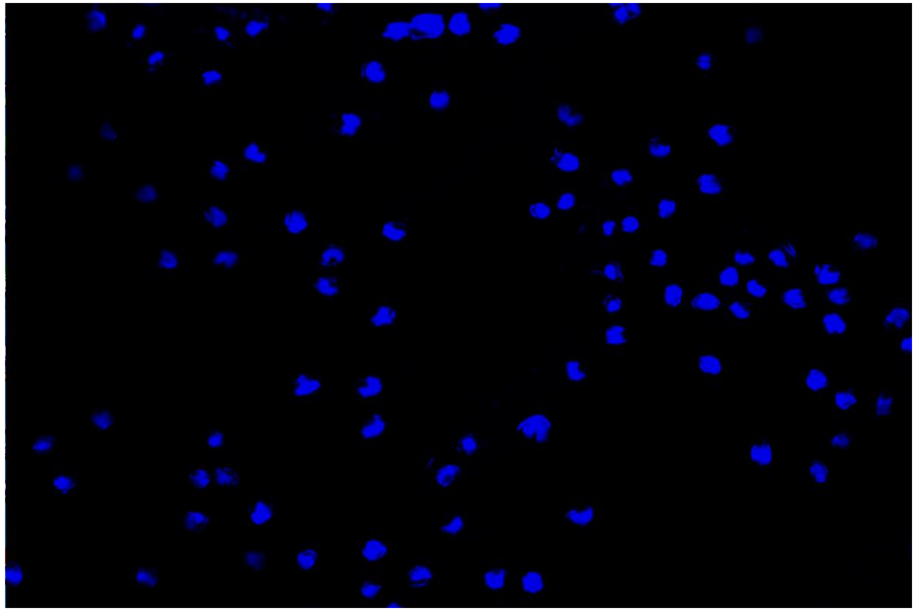

Supplementary Figure 3B

DMEM+H/R DAPI

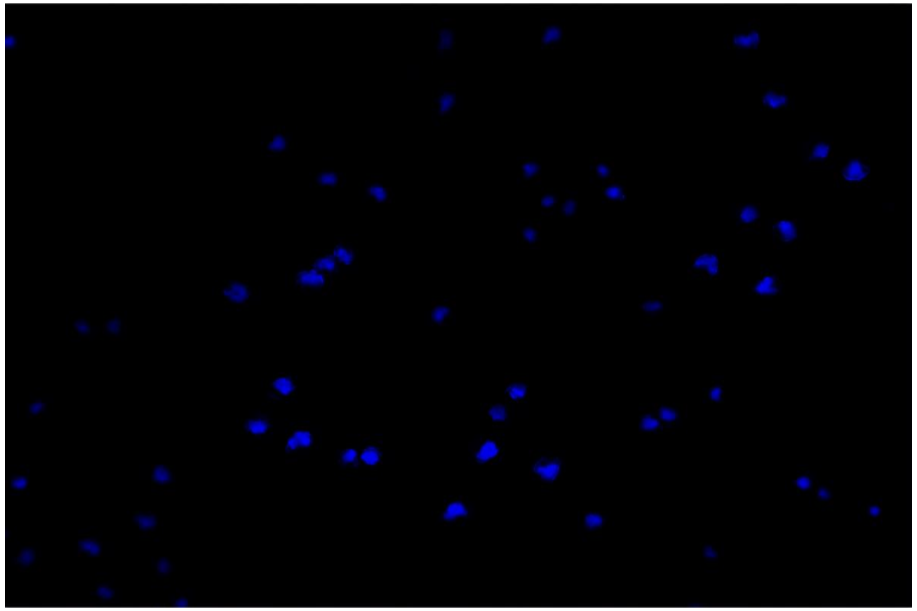

Supplementary Figure 3B

DMEM-FBS Merge

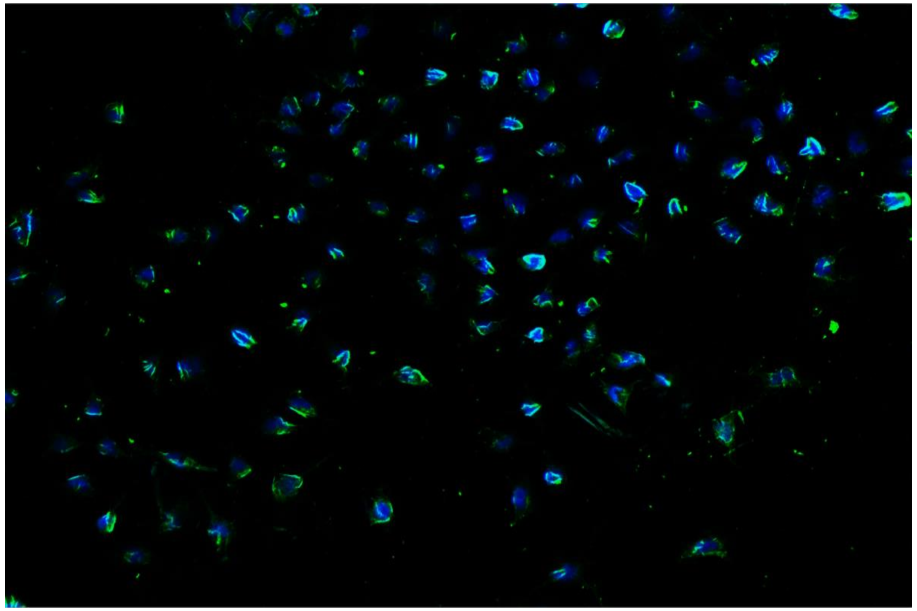

Supplementary Figure 3B

DMEM Merge

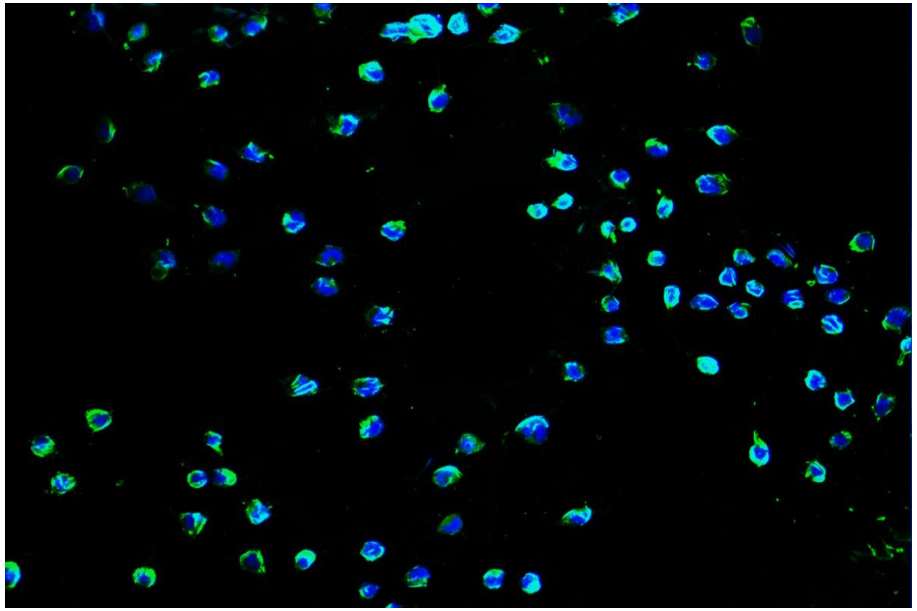

Supplementary Figure 3B

DMEM+H/R Merge

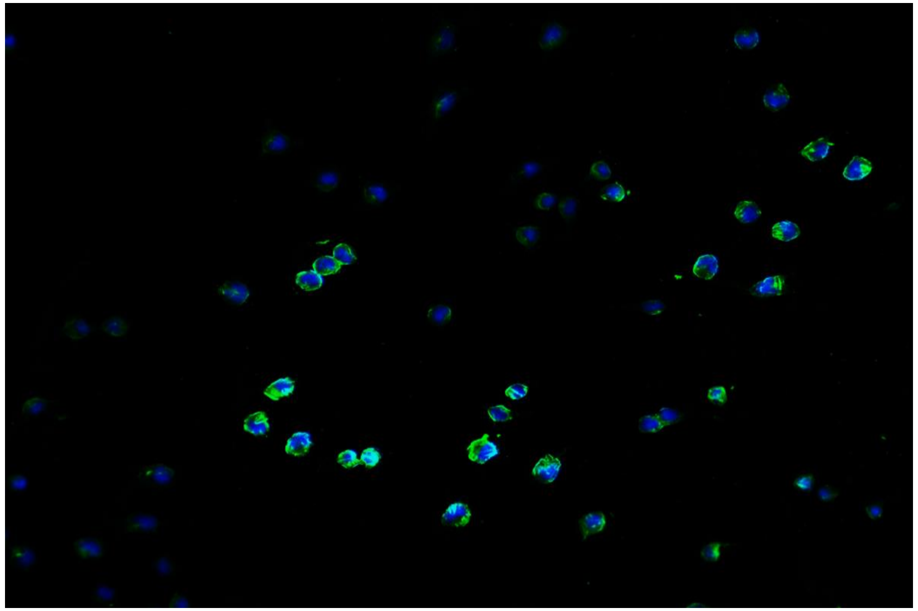

Supplementary Figure 4A

Control

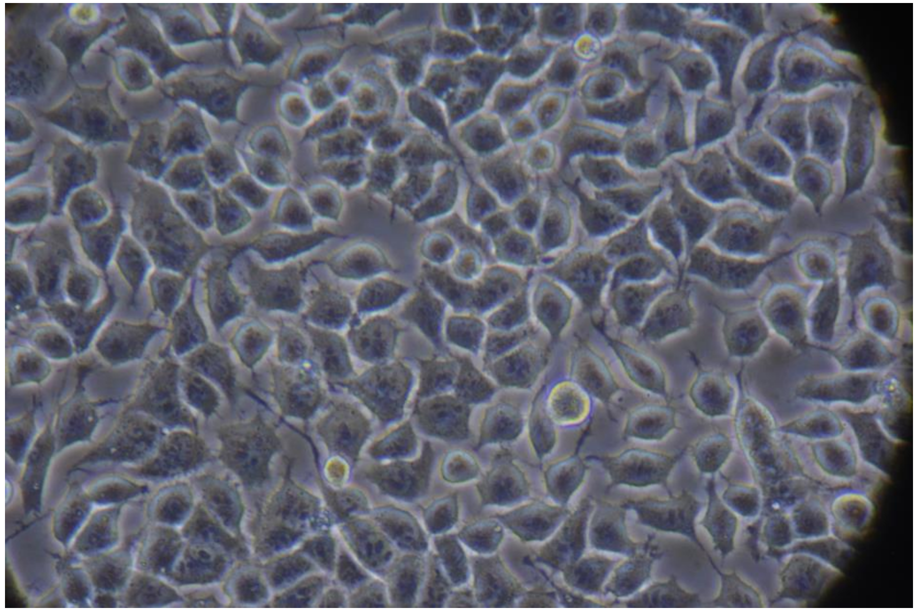

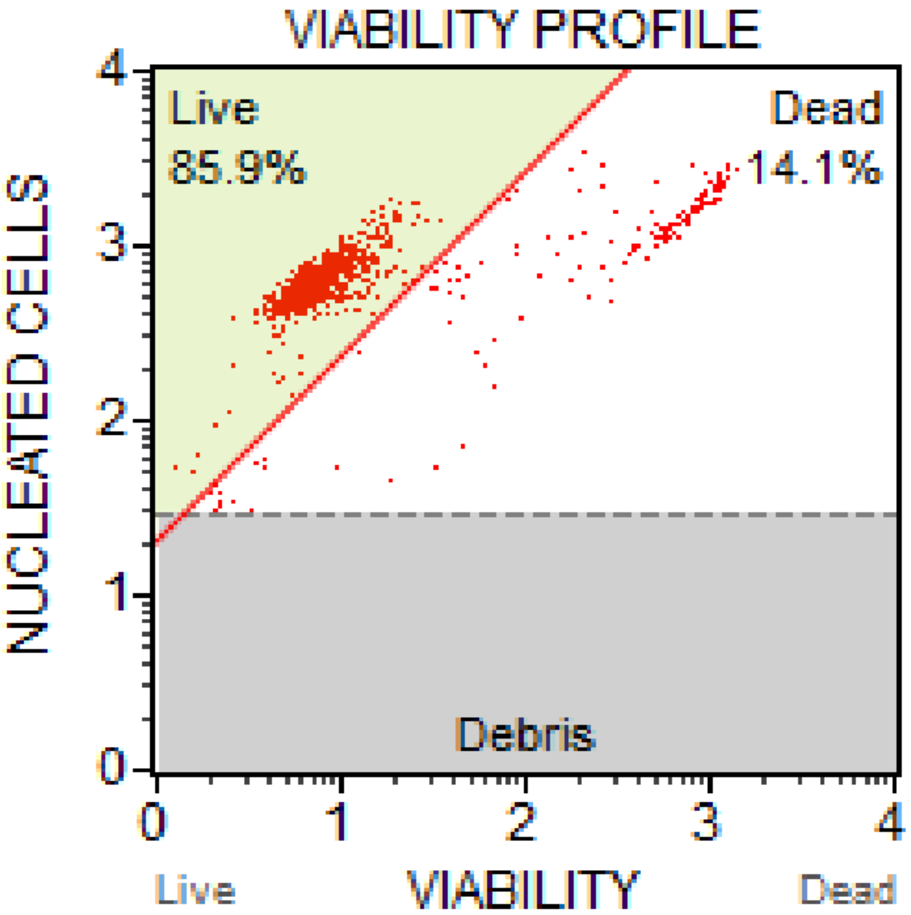

Supplementary Figure 4A

NC siRNA

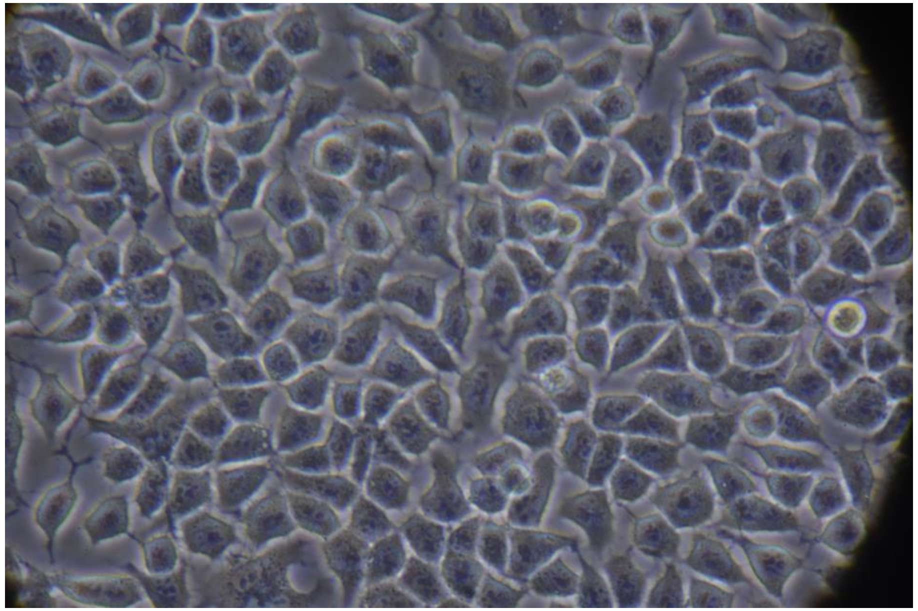

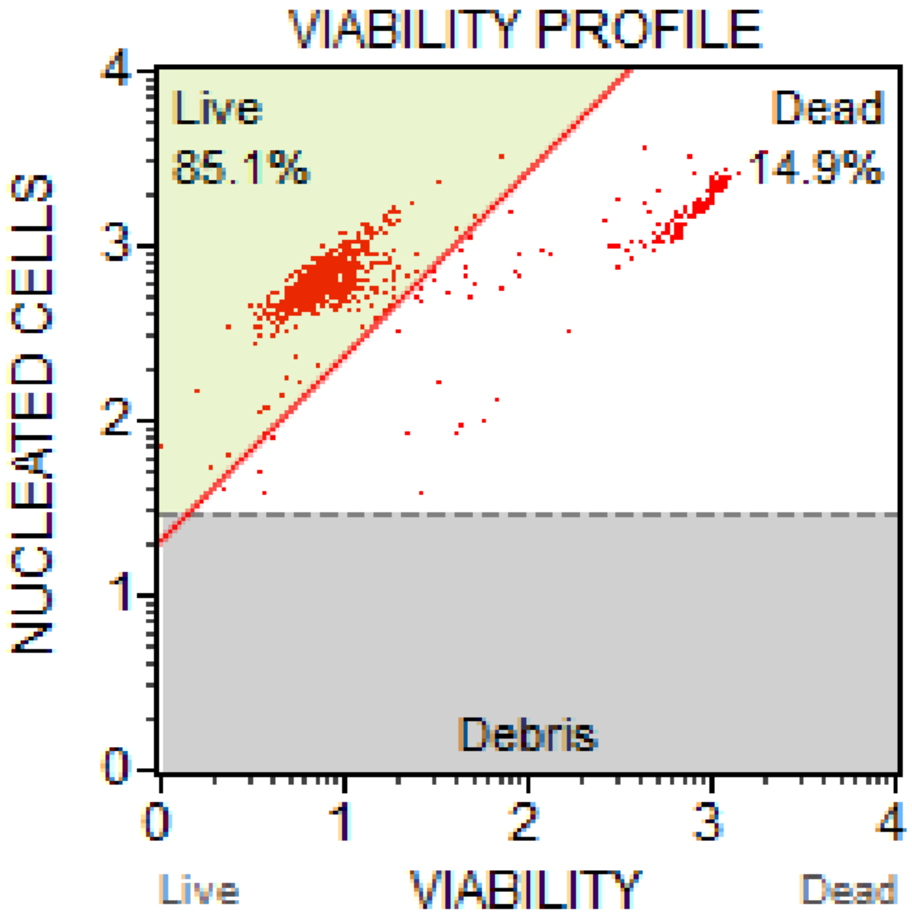

Supplementary Figure 4A

GAS6 siRNA

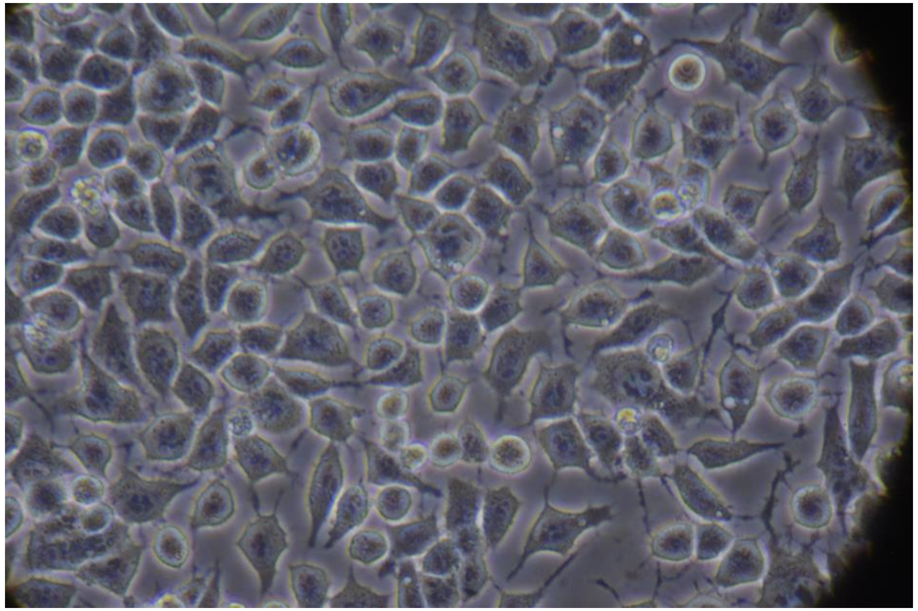

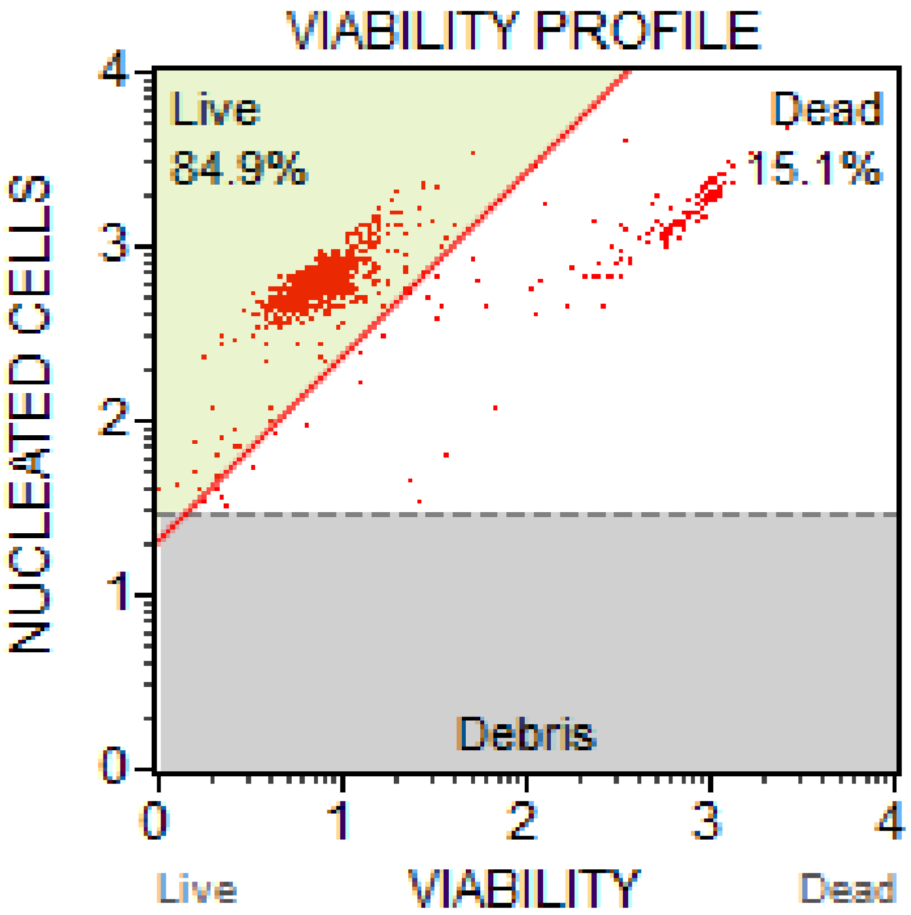

Supplement: Supplementary file 1 [file DataSheet2.PDF]
