# Supplementary figures and images for: Neuroprotective Effects of Omentin-1 Against Cerebral Hypoxia/Reoxygenation Injury via Activating GAS6/Axl Signaling Pathway in Neuroblastoma Cells
Source: Front Cell Dev Biol. 2022 Jan 24;9:784035. doi: 10.3389/fcell.2021.784035 (PMC8818945; doi:10.3389/fcell.2021.784035)

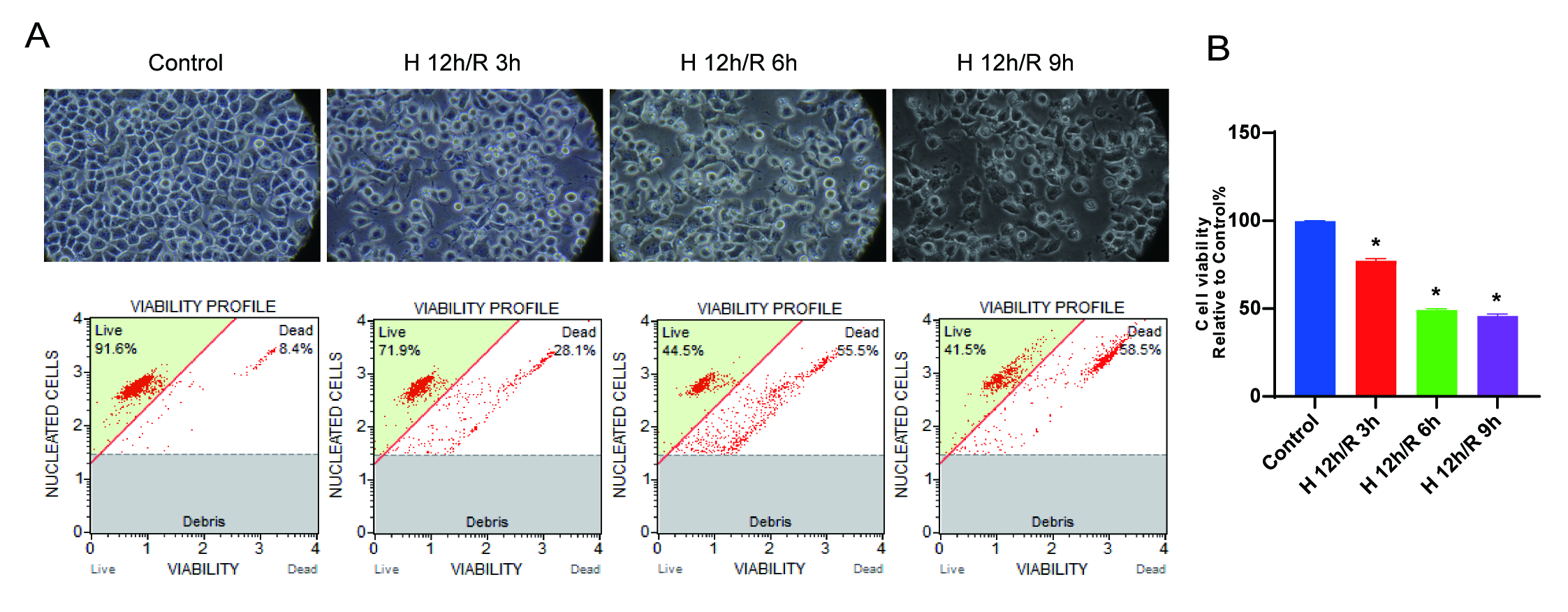

Supplement: Supplementary file 2 [file DataSheet4.zip › Supplementary Figure/Supplementary Figure S1.tif]

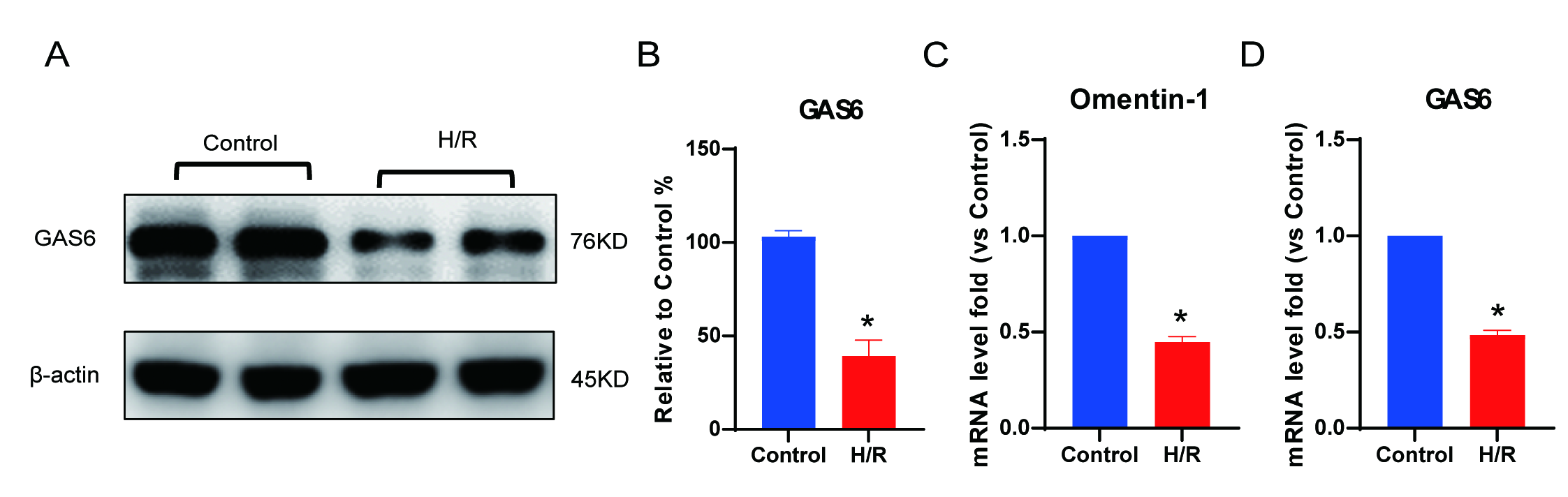

Supplement: Supplementary file 2 [file DataSheet4.zip › Supplementary Figure/Supplementary Figure S2.tif]

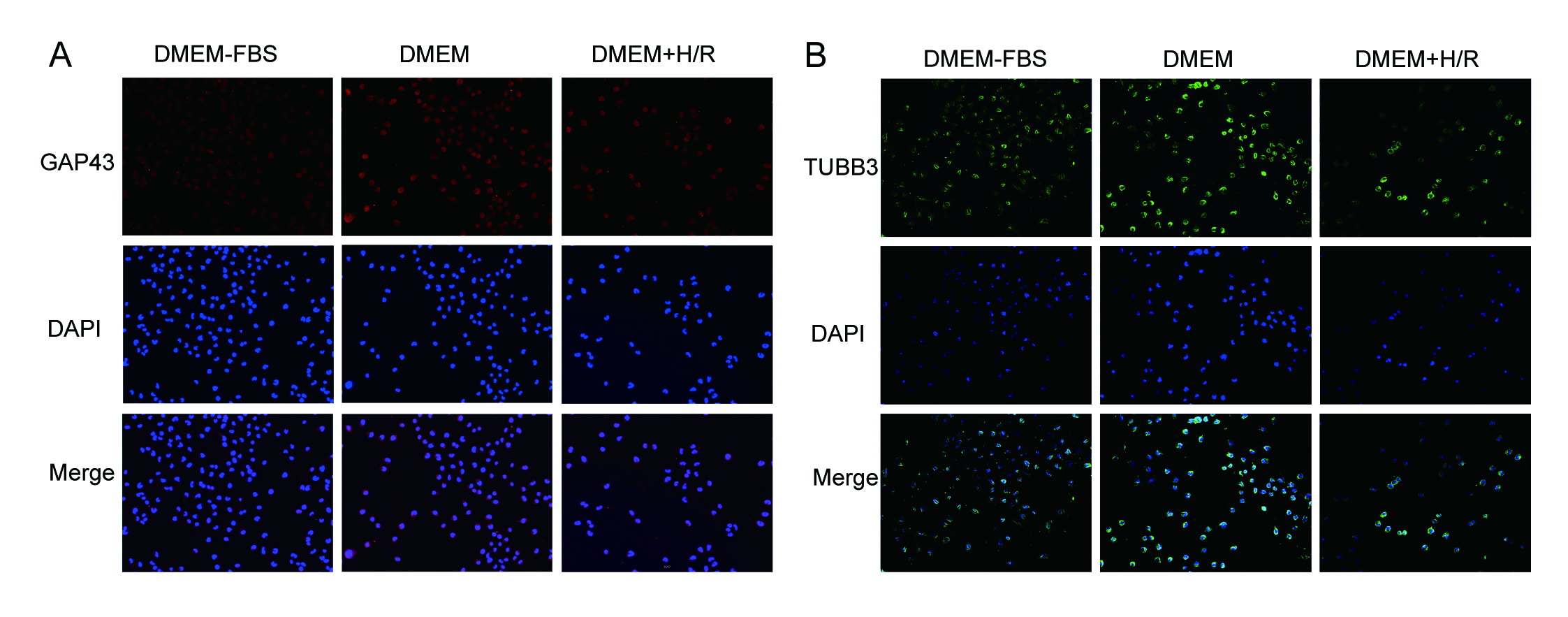

Supplement: Supplementary file 2 [file DataSheet4.zip › Supplementary Figure/Supplementary Figure S3.tif]

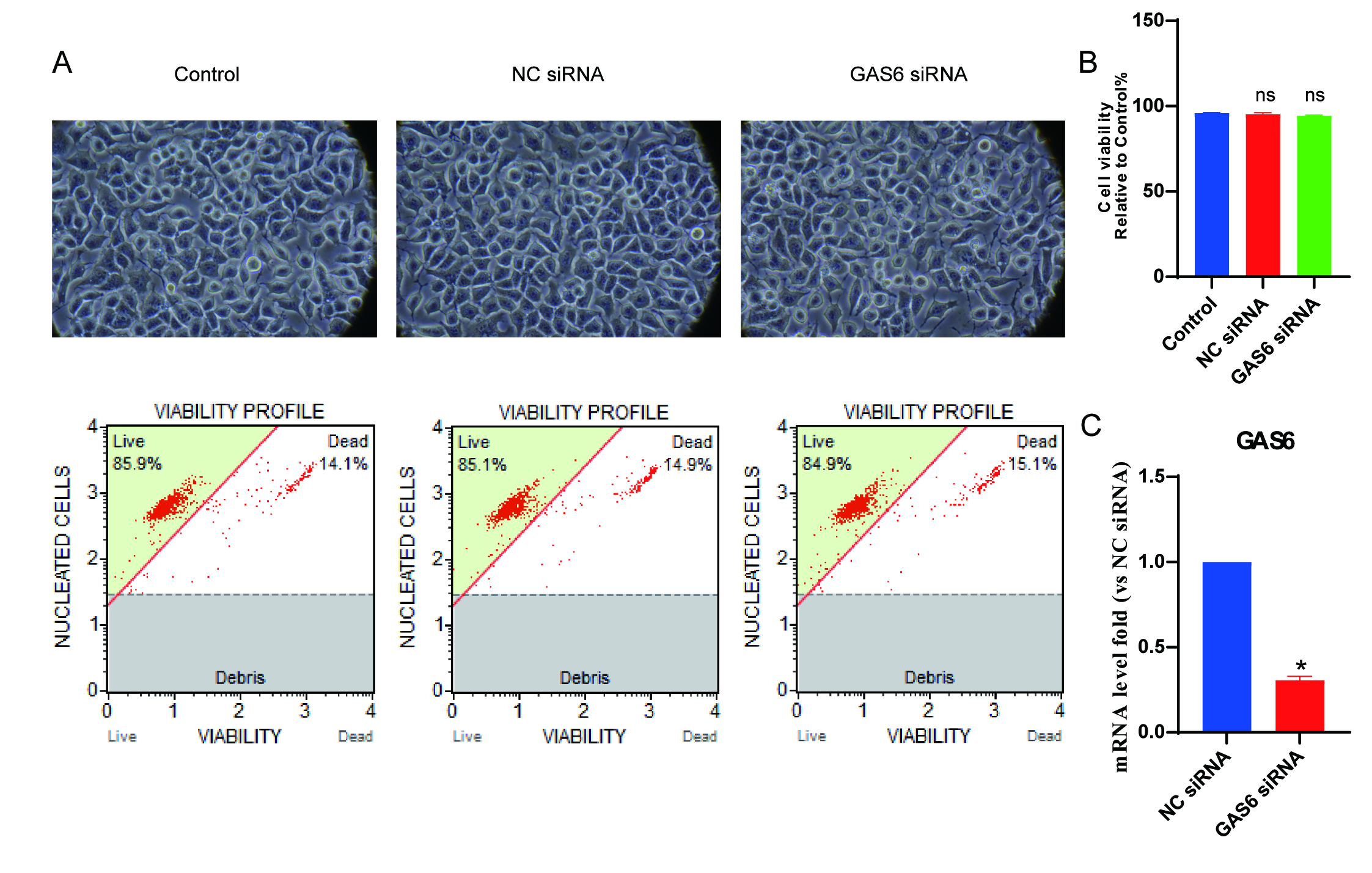

Supplement: Supplementary file 2 [file DataSheet4.zip › Supplementary Figure/Supplementary Figure S4.tif]
