# Supplementary material for: Neuroprotective Effects of Omentin-1 Against Cerebral Hypoxia/Reoxygenation Injury via Activating GAS6/Axl Signaling Pathway in Neuroblastoma Cells
Source: Front Cell Dev Biol. 2022 Jan 24;9:784035. doi: 10.3389/fcell.2021.784035 (PMC8818945; doi:10.3389/fcell.2021.784035)

Neuroprotective effects of omentin-1 against cerebral hypoxia/reoxygenation injury via activating GAS6/Axl signaling pathway in neuroblastoma cells

Figure 1D

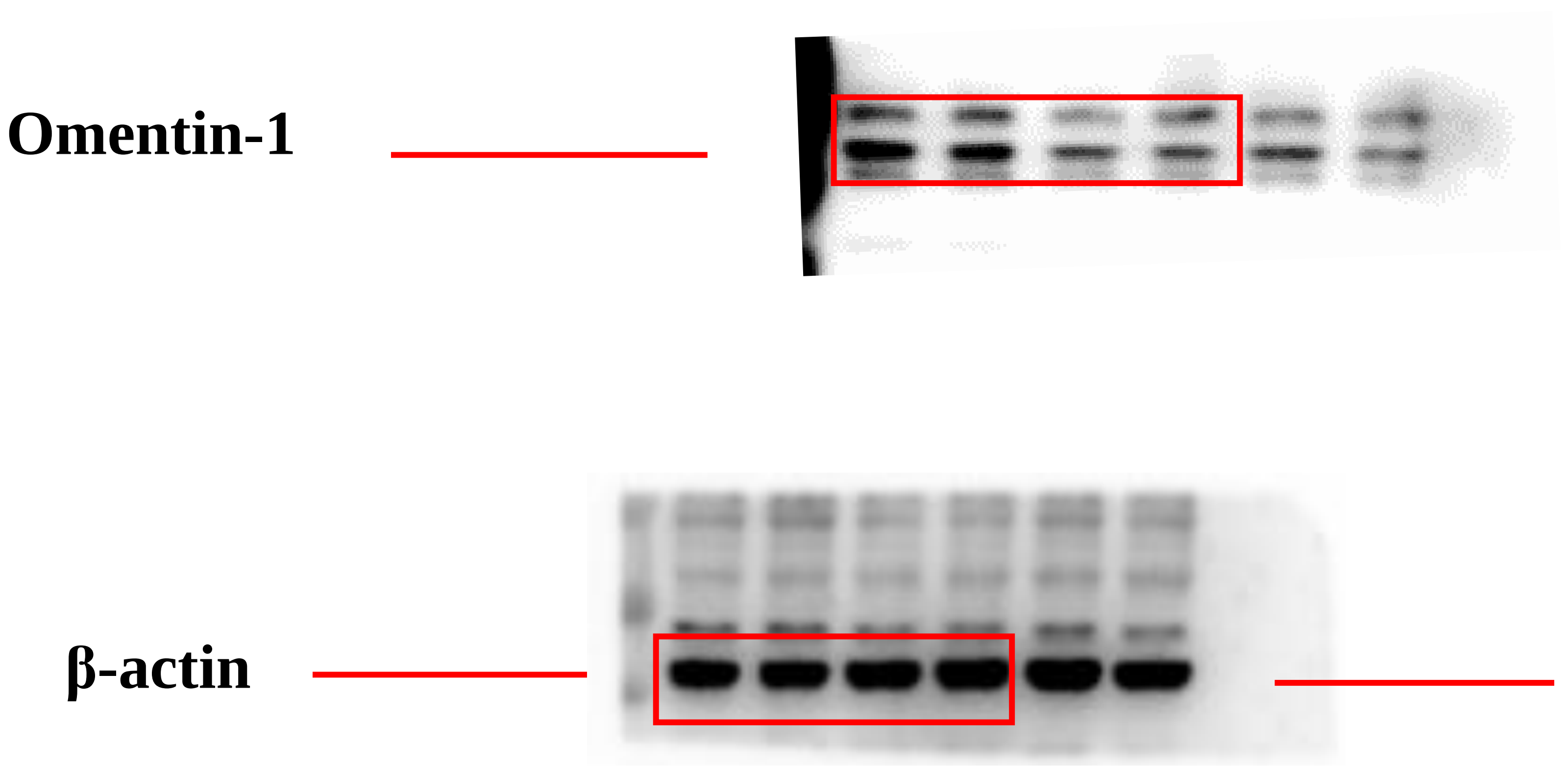

Figure 5A

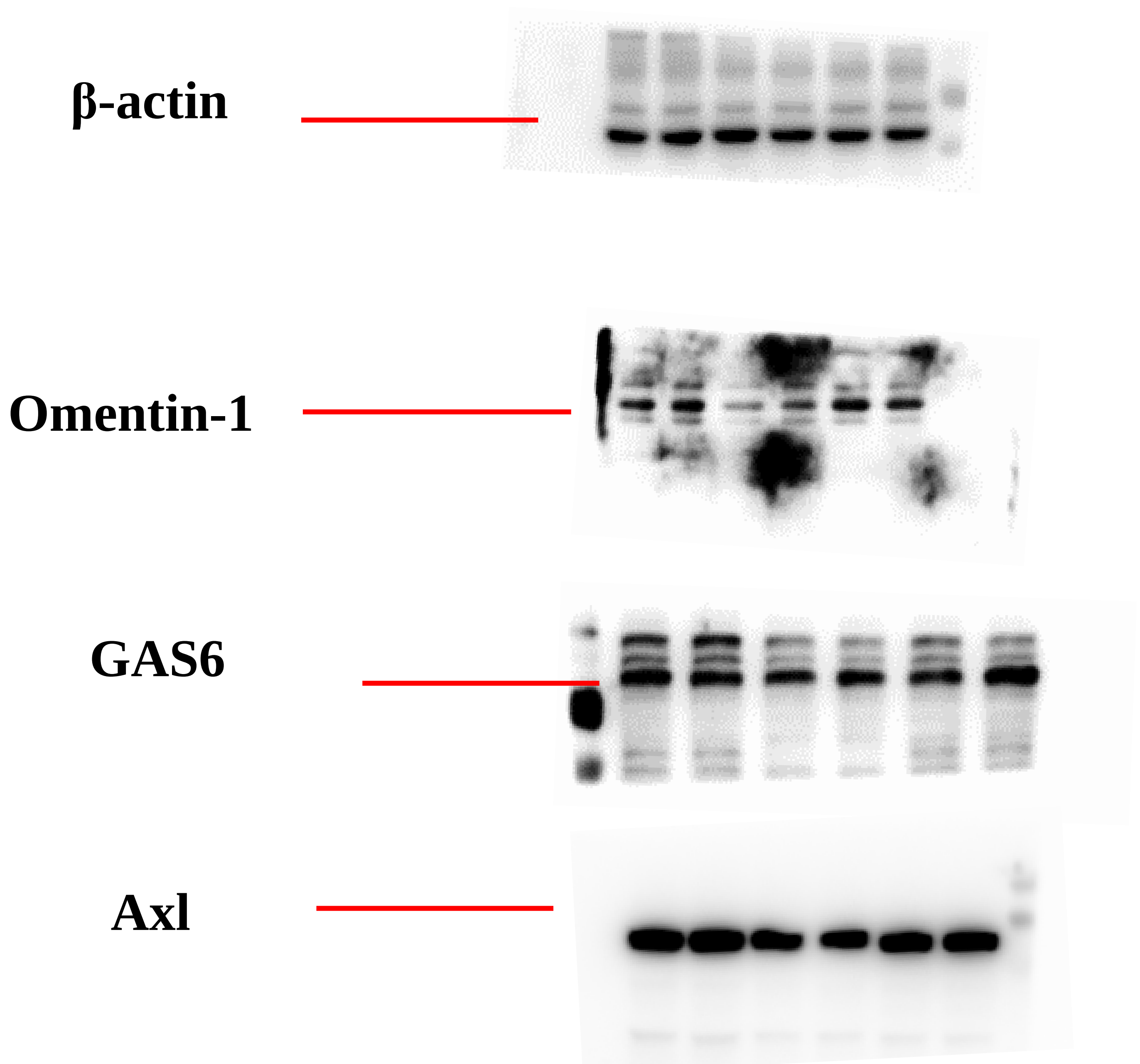

Figure 5A

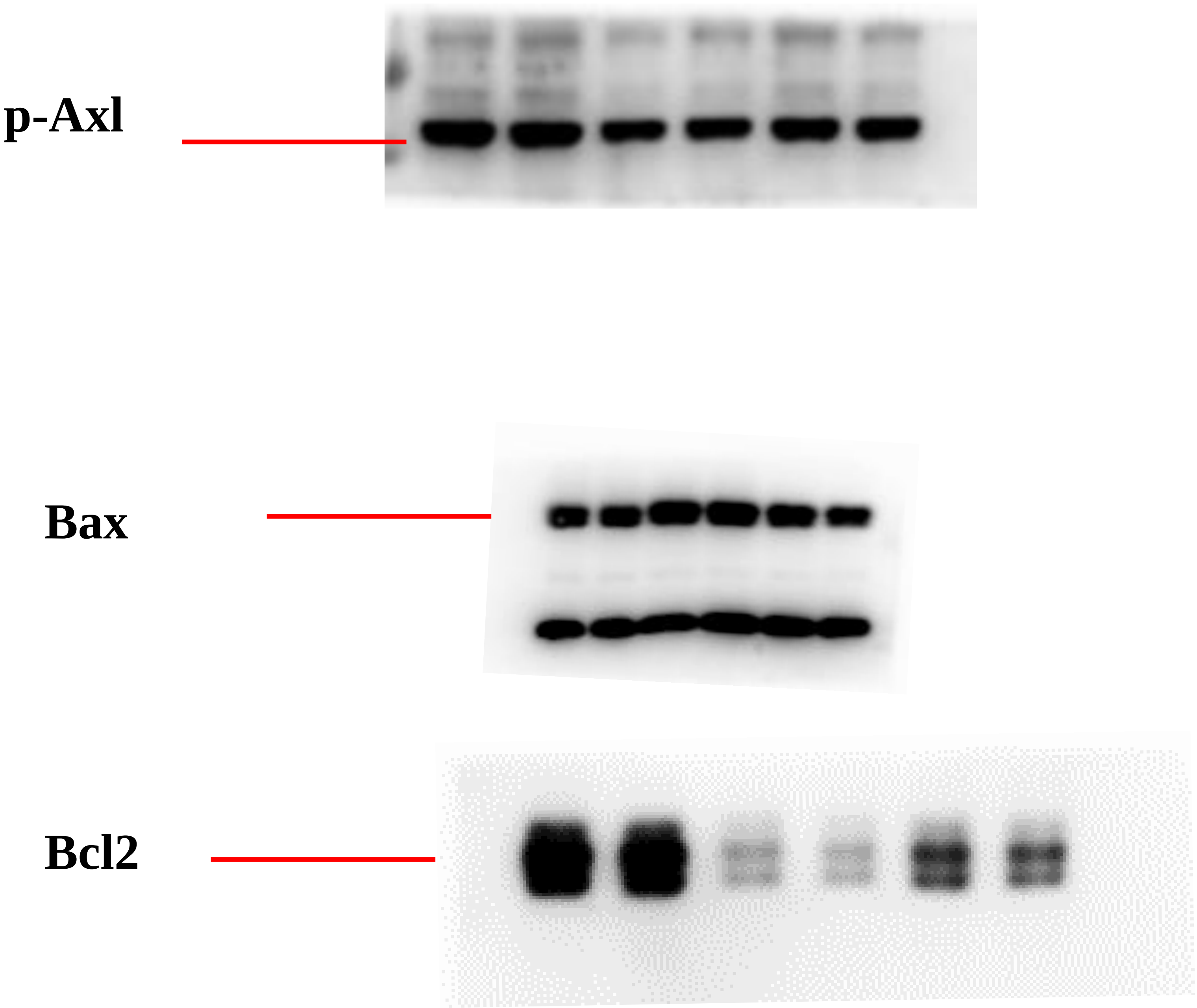

Figure 5A

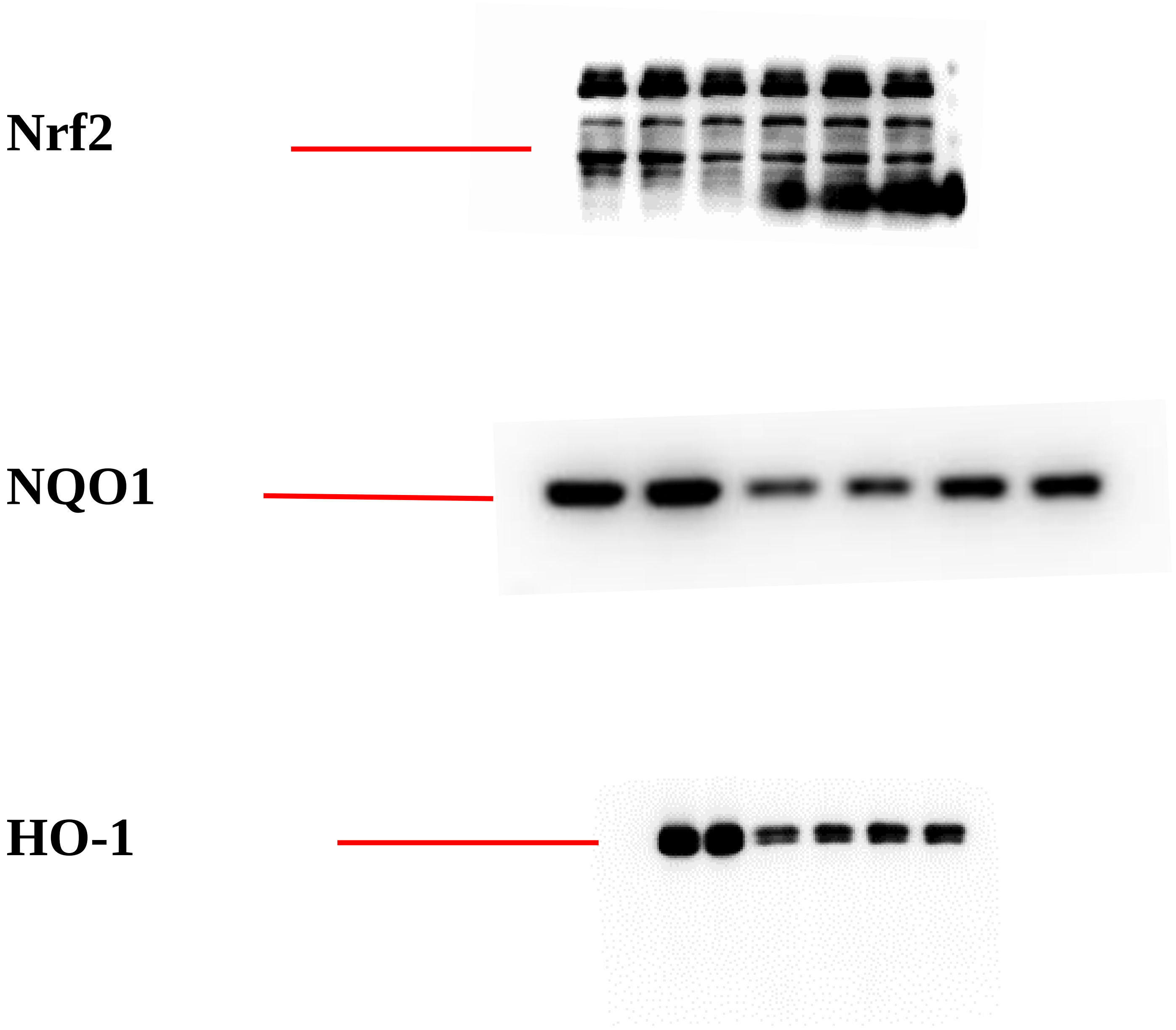

Figure 7A

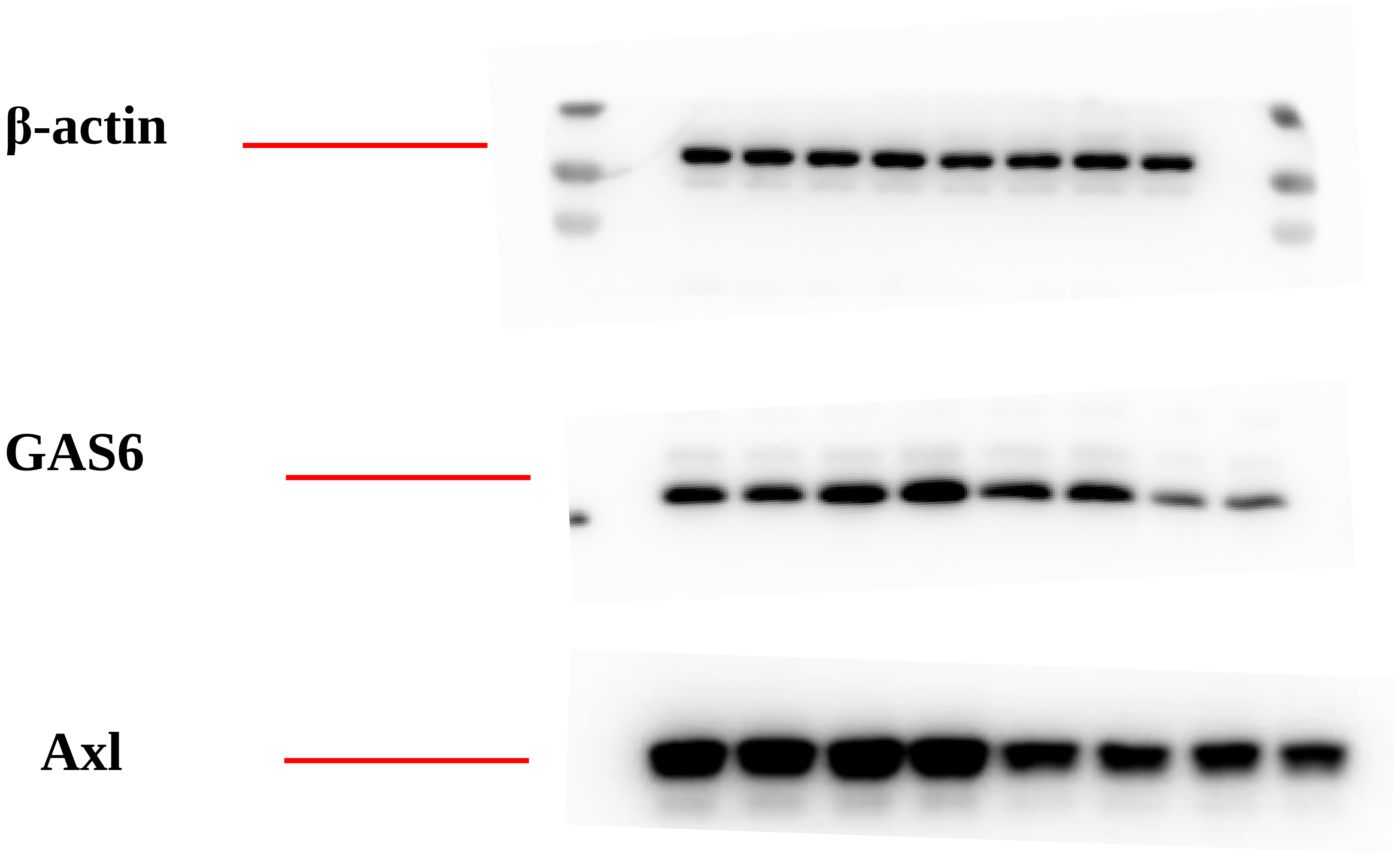

Figure 7A

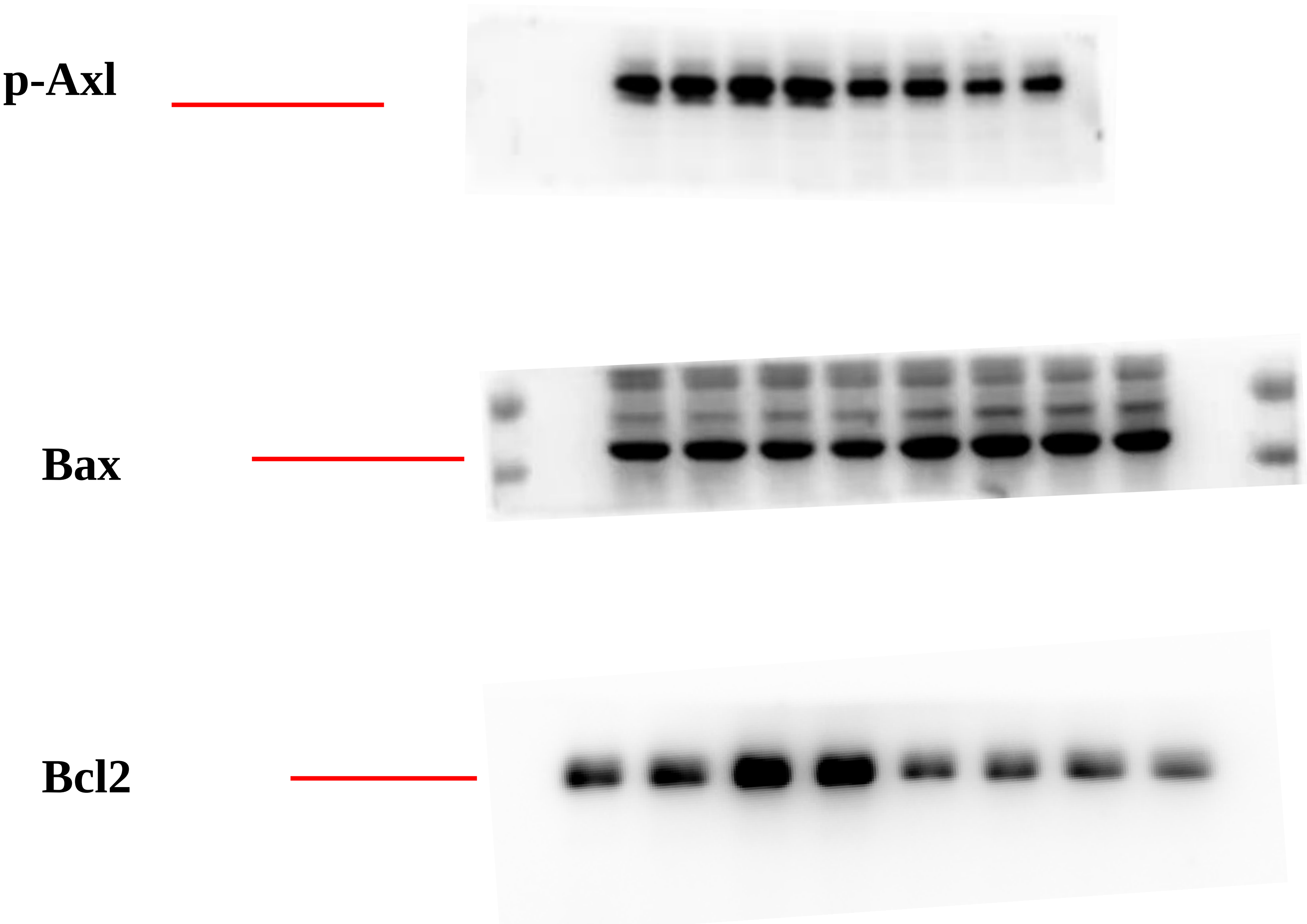

Figure 7A

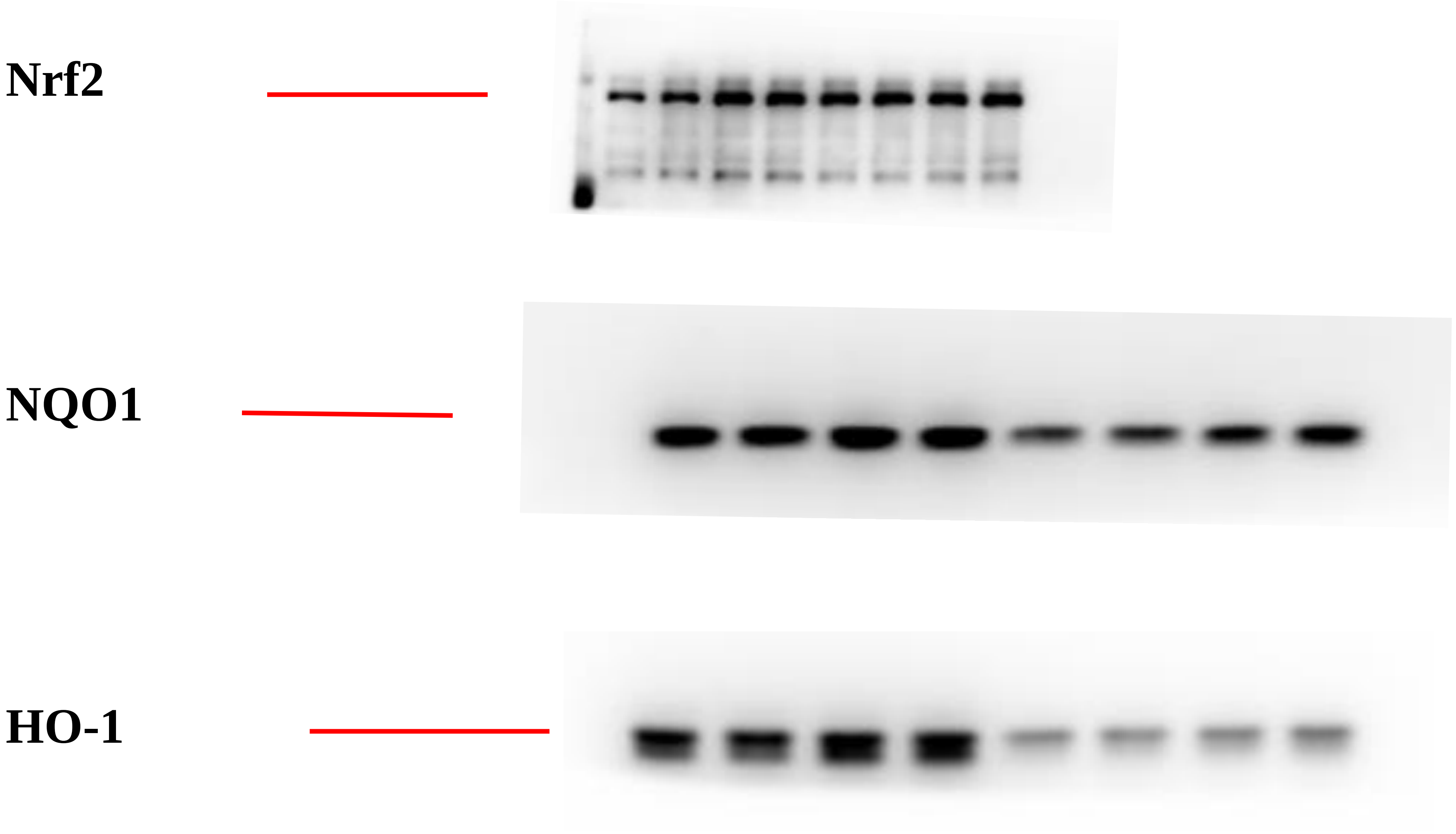

Figure 8A

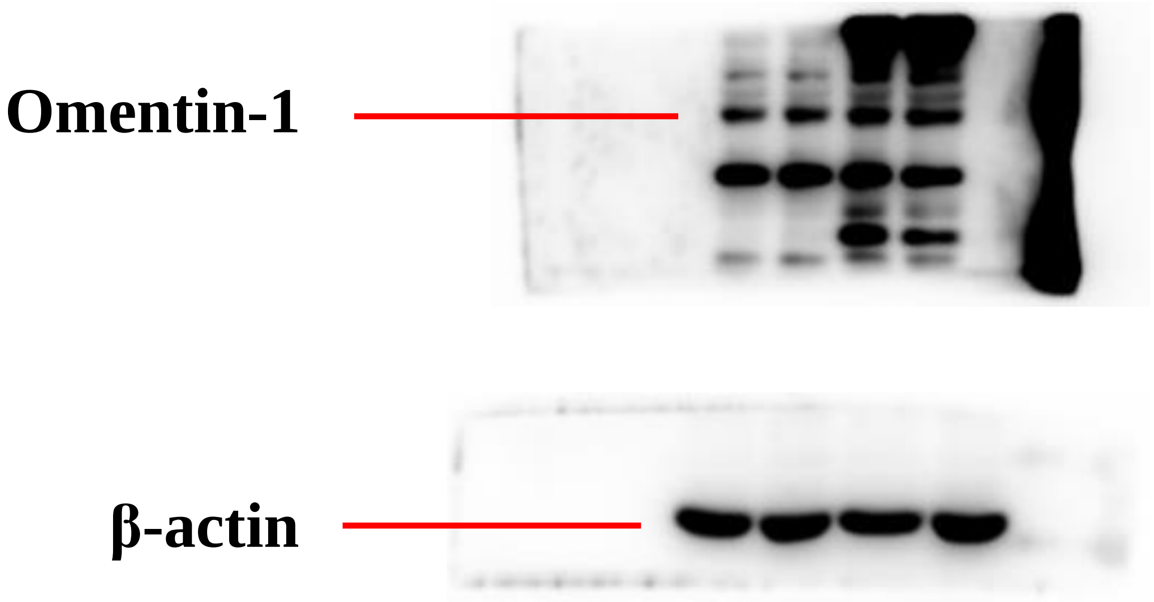

Supplementary Figure 2A

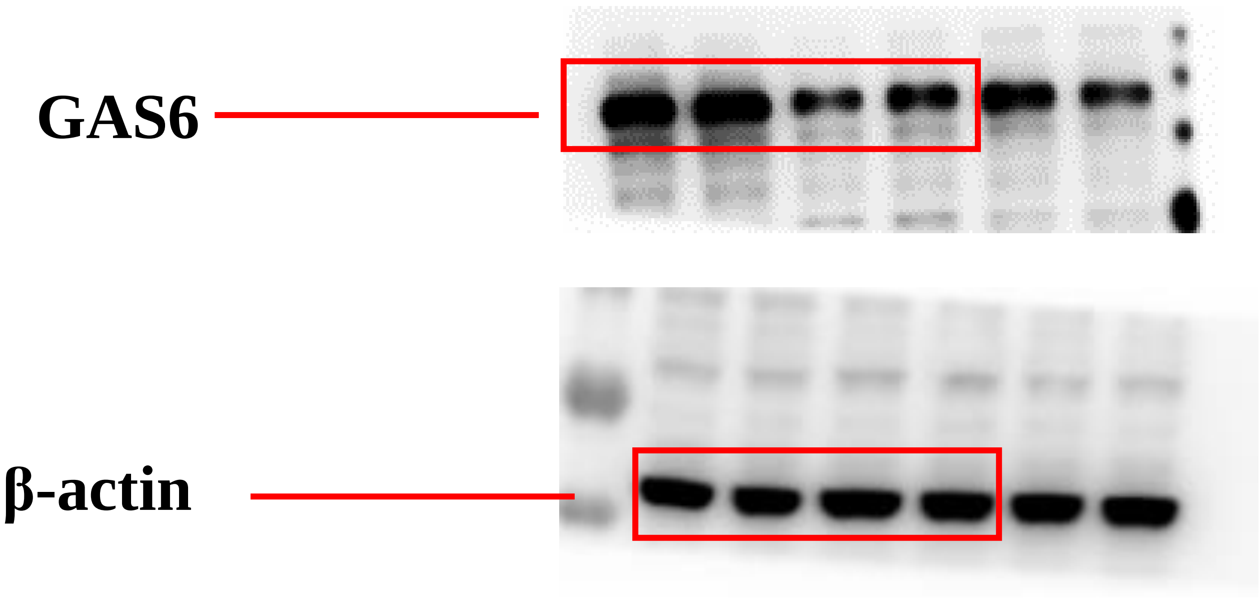

Supplement: Supplementary file 3 [file DataSheet3.PDF]
