# Supplementary material for: Neuroprotective Effects of Omentin-1 Against Cerebral Hypoxia/Reoxygenation Injury via Activating GAS6/Axl Signaling Pathway in Neuroblastoma Cells
Source: Front Cell Dev Biol. 2022 Jan 24;9:784035. doi: 10.3389/fcell.2021.784035 (PMC8818945; doi:10.3389/fcell.2021.784035)
Supplement: Supplementary file 4 [file DataSheet1.PDF]

Neuroprotective effects of omentin-1 against cerebral hypoxia/reoxygenation injury via activating GAS6/Axl signaling pathway in neuroblastoma cells

Figure 4A

Control

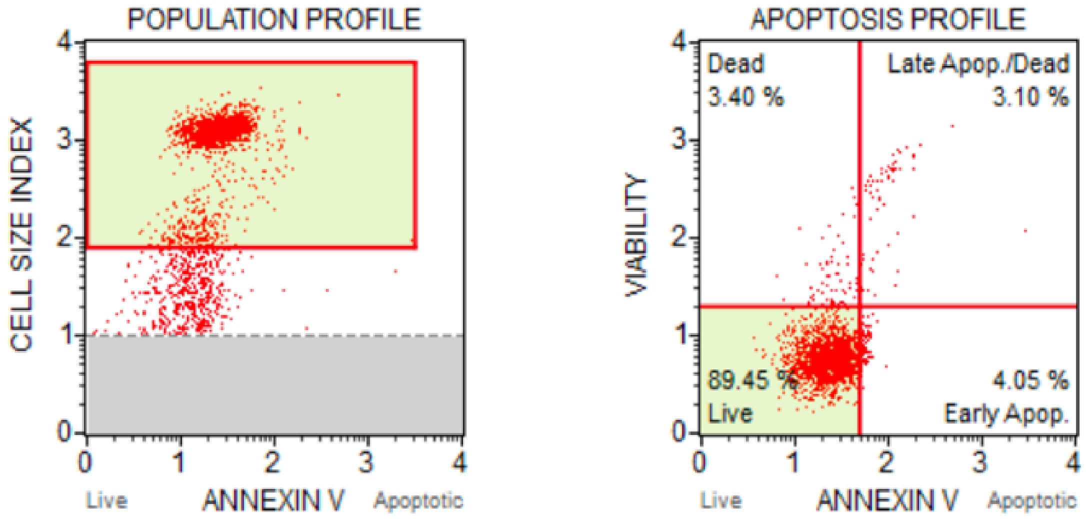

	Cell Conc. (Cells / mL)	% Gated
Live (LL) :	6.64E+05	89.45 %
Early Apoptotic (LR) :	3.01E+04	4.05 %
Late Apop./ Dead (UR) :	2.30E+04	3.10 %
Debris (UL) :	2.53E+04	3.40 %
Total Apoptotic :	5.31E+04	7.15 %

Figure 4A

H/R

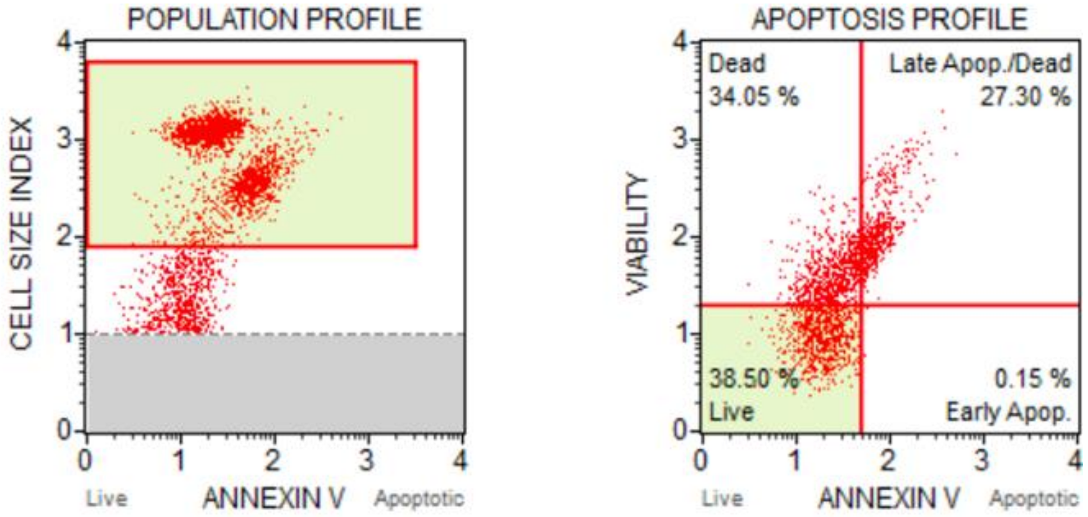

	Cell Conc. (Cells / mL)	% Gated
Live (LL) :	1.32E+05	38.50 %
Early Apoptotic (LR) :	5.14E+02	0.15 %
Late Apop./ Dead (UR) :	9.35E+04	27.30 %
Debris (UL) :	1.17E+05	34.05 %
Total Apoptotic :	9.40E+04	27.45 %

Figure 4A

750ng/ml rh-omentin +H/R

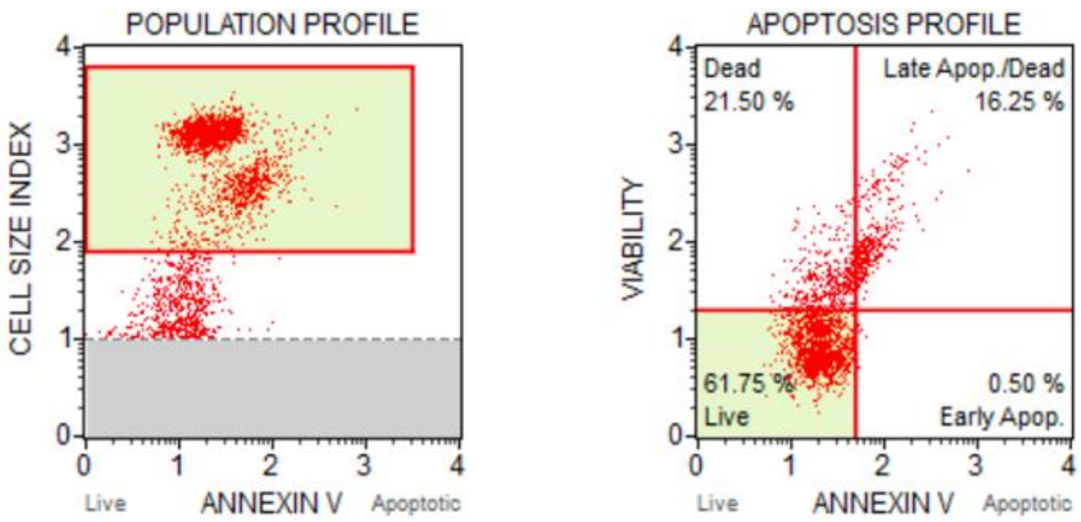

	Cell Conc. (Cells / mL)	% Gated
Live (LL) :	2.30E+05	61.75 %
Early Apoptotic (LR) :	1.86E+03	0.50 %
Late Apop./ Dead (UR) :	6.06E+04	16.25 %
Debris (UL) :	8.02E+04	21.50 %
Total Apoptotic :	6.24E+04	16.75 %

Figure 6E

NC siRNA H/R

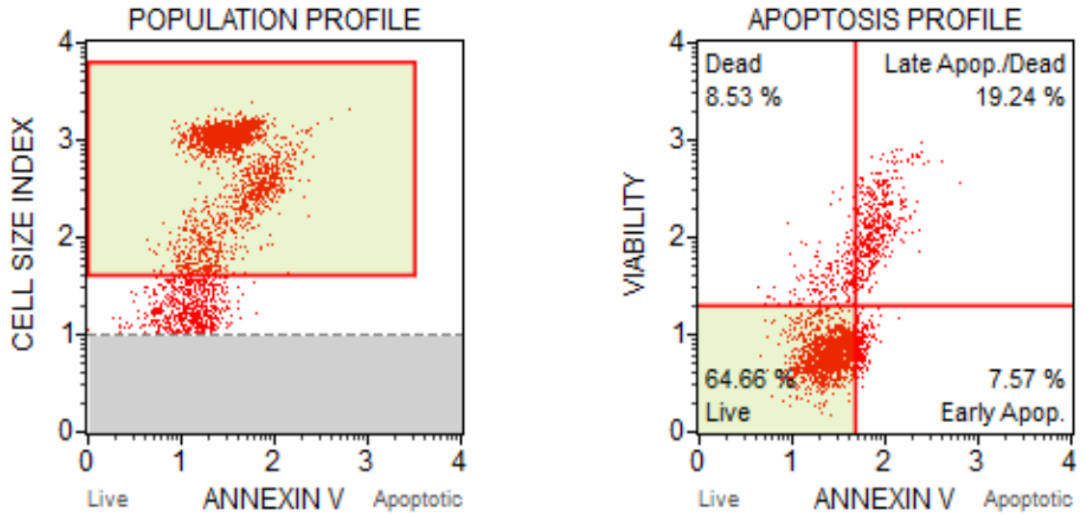

	Cell Conc. (Cells / mL)	% Gated
Live (LL) :	2.92E+05	64.66 %
Early Apoptotic (LR) :	3.42E+04	7.57 %
Late Apop./ Dead (UR) :	8.70E+04	19.24 %
Debris (UL) :	3.86E+04	8.53 %
Total Apoptotic :	1.21E+05	26.81 %

Figure 6E

NC siRNA H/R + rh-omentin

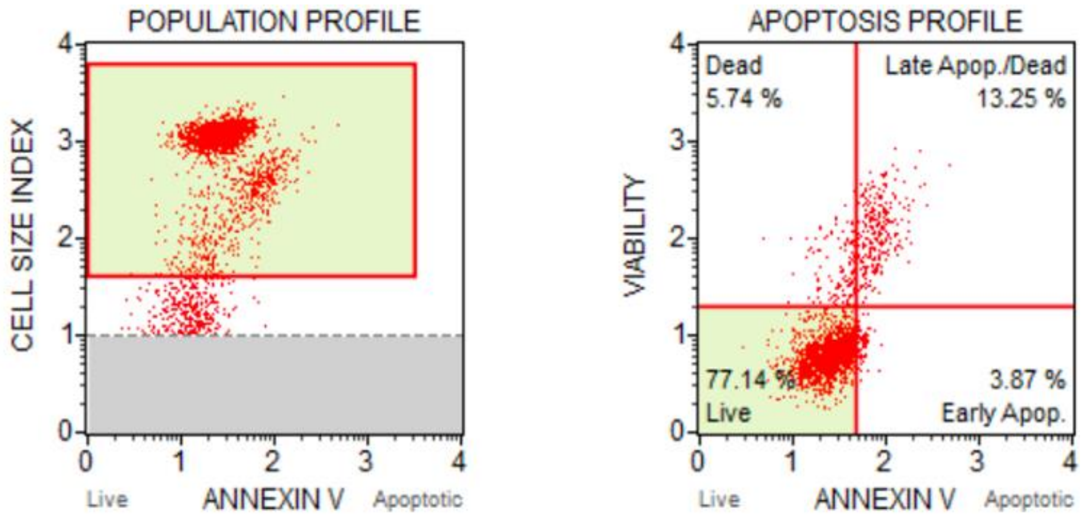

	Cell Conc. (Cells / mL)	% Gated
Live (LL) :	3.75E+05	77.14 %
Early Apoptotic (LR) :	1.88E+04	3.87 %
Late Apop./ Dead (UR) :	6.44E+04	13.25 %
Debris (UL) :	2.79E+04	5.74 %
Total Apoptotic :	8.33E+04	17.12 %

Figure 6E

GAS6 siRNA H/R + rh-omentin

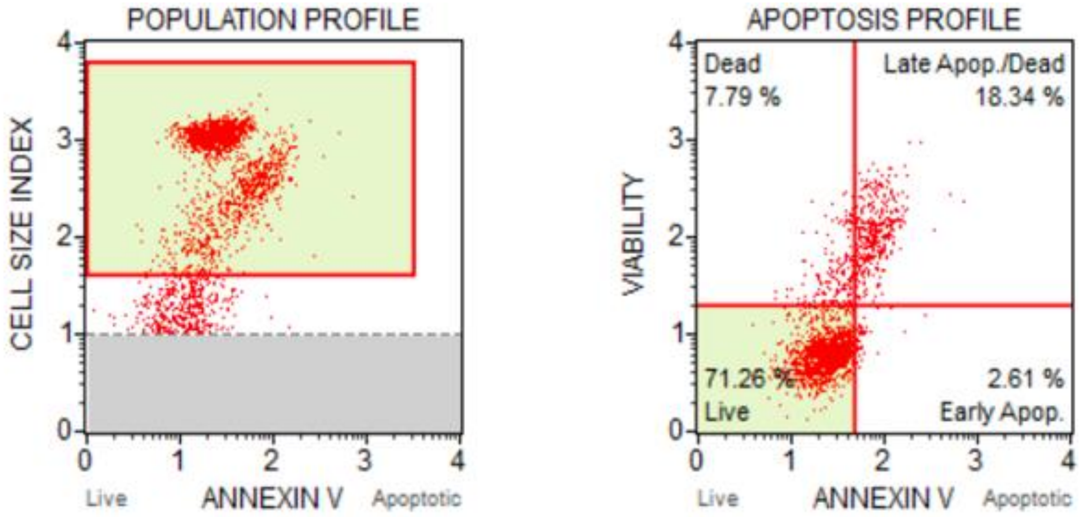

	Cell Conc. (Cells / mL)	% Gated
Live (LL) :	3.09E+05	71.26 %
Early Apoptotic (LR) :	1.13E+04	2.61 %
Late Apop./ Dead (UR) :	7.95E+04	18.34 %
Debris (UL) :	3.38E+04	7.79 %
Total Apoptotic :	9.08E+04	20.95 %

Figure 6E

GAS6 siRNA H/R

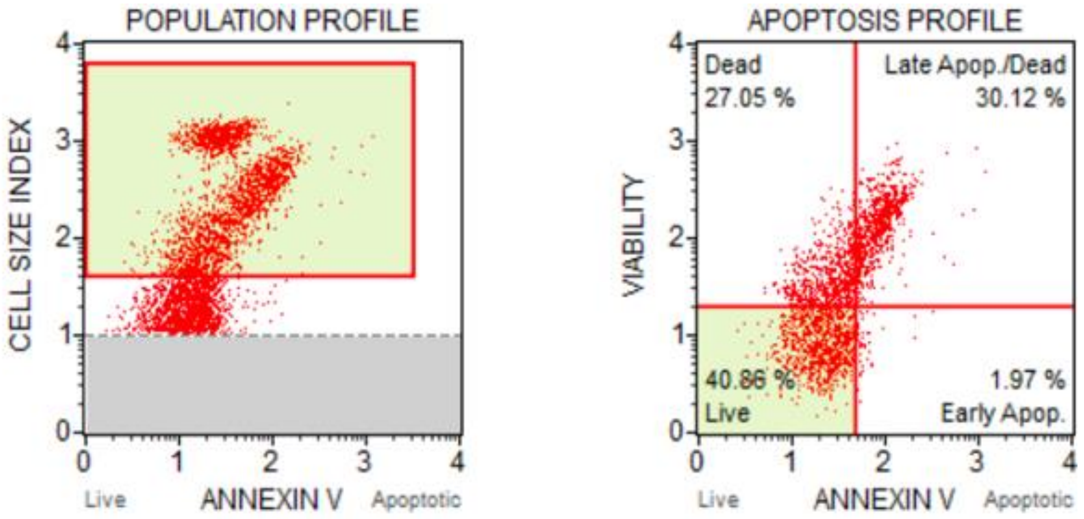

	Cell Conc. (Cells / mL)	% Gated
Live (LL) :	8.98E+04	40.86 %
Early Apoptotic (LR) :	4.32E+03	1.97 %
Late Apop./ Dead (UR) :	6.62E+04	30.12 %
Debris (UL) :	5.94E+04	27.05 %
Total Apoptotic :	7.05E+04	32.09 %

Figure 9C

Control N2a-Mock

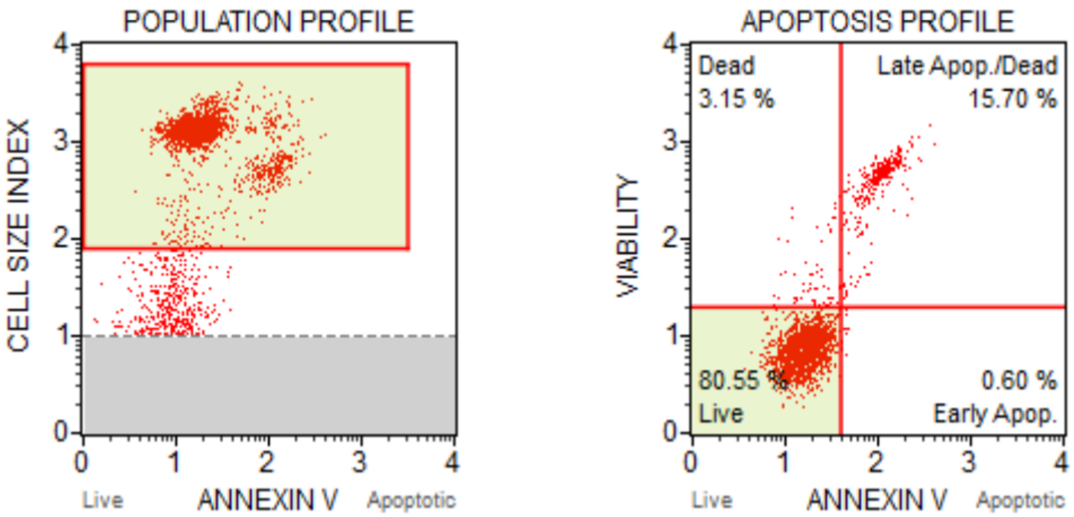

	Cell Conc. (Cells / mL)	% Gated
Live (LL) :	9.27E+05	80.55 %
Early Apoptotic (LR) :	6.91E+03	0.60 %
Late Apop./ Dead (UR) :	1.81E+05	15.70 %
Debris (UL) :	3.63E+04	3.15 %
Total Apoptotic :	1.88E+05	16.30 %

Figure 9C

Control N2a-ITLN

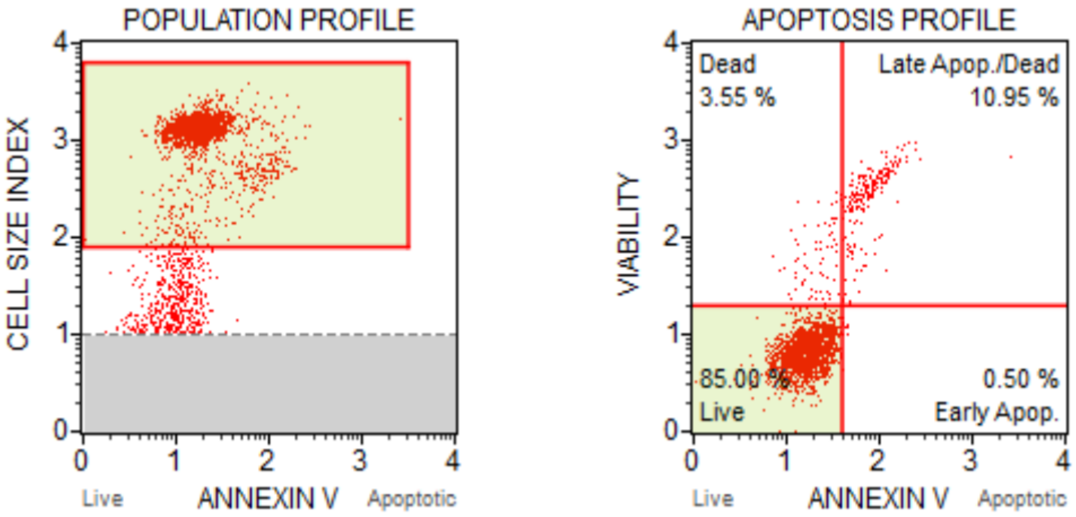

	Cell Conc. (Cells / mL)	% Gated
Live (LL) :	1.18E+06	85.00 %
Early Apoptotic (LR) :	6.91E+03	0.50 %
Late Apop./ Dead (UR) :	1.51E+05	10.95 %
Debris (UL) :	4.91E+04	3.55 %
Total Apoptotic :	1.58E+05	11.45 %

Figure 9C

H/R N2a-Mock

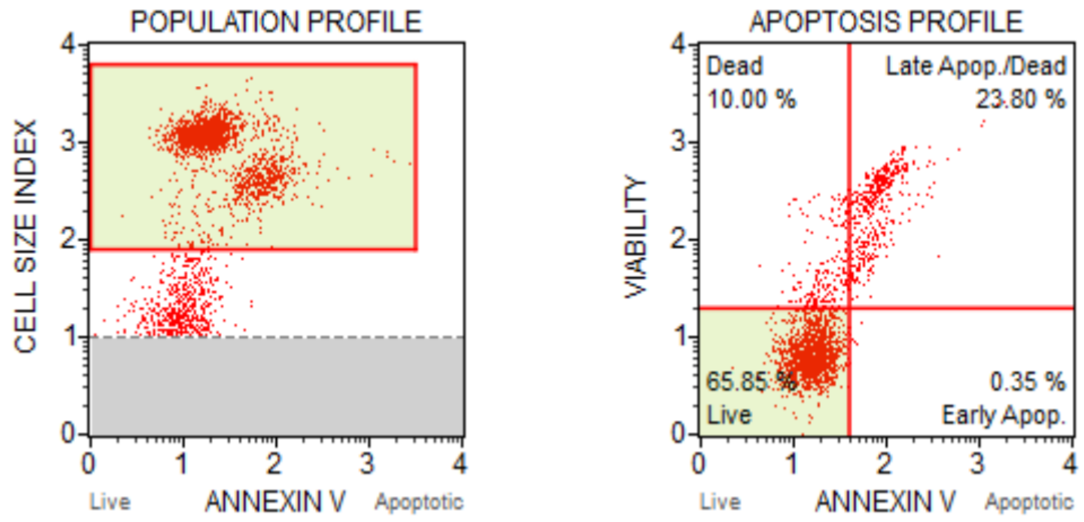

	Cell Conc. (Cells / mL)	% Gated
Live (LL) :	3.73E+05	65.85 %
Early Apoptotic (LR) :	1.98E+03	0.35 %
Late Apop./ Dead (UR) :	1.35E+05	23.80 %
Debris (UL) :	5.66E+04	10.00 %
Total Apoptotic :	1.37E+05	24.15 %

Figure 9C

H/R N2a-ITLN

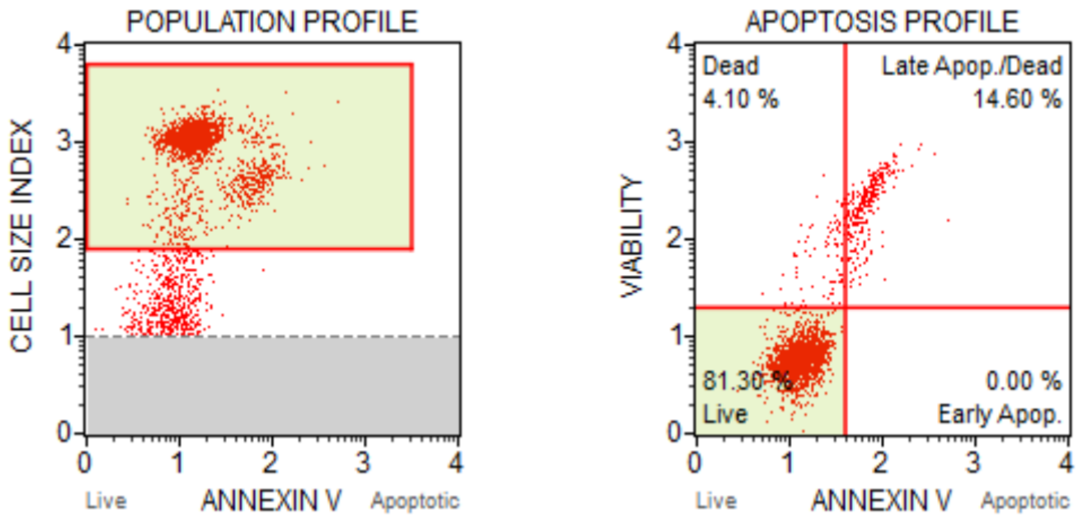

	Cell Conc. (Cells / mL)	% Gated
Live (LL) :	8.36E+05	81.30 %
Early Apoptotic (LR) :	0.00E+00	0.00 %
Late Apop./ Dead (UR) :	1.50E+05	14.60 %
Debris (UL) :	4.21E+04	4.10 %
Total Apoptotic :	1.50E+05	14.60 %
